# Phenolic Compounds From the Stems and Leaves of *Berchemia lineata* (L.) DC

**DOI:** 10.3389/fchem.2022.889441

**Published:** 2022-04-14

**Authors:** Yitong Li, Yu Chen, Wenli Xie, Xueni Li, Gui Mei, Jing Xu, Xiangpei Zhao, Hongli Teng, Guangzhong Yang

**Affiliations:** ^1^ School of Pharmaceutical Sciences, South-Central University for Nationalities, Wuhan, China; ^2^ College of Chemistry and Material Sciences, South-Central University for Nationalities, Wuhan, China; ^3^ Guangxi International Zhuang Medical Hospital, Nanning, China

**Keywords:** *Berchemia lineata*, *Berchemia*, phenolic compounds, antihyperglycemic effect, *α*-glucosidase inhibitory activity

## Abstract

Eight new phenolic compounds, named bercheminols A-H (**1–8**), and eleven known analogues were isolated from the stems and leaves of *Berchemia lineata* (L.) DC. Their structures including the absolute configurations were elucidated by extensive spectroscopic analysis, chemical method, and quantum chemical calculations. Compound **1** possesses an unprecedented 3,4-dihydro-11H-benzo[b]pyrano[4,3-e] oxepin-11-one skeleton. The other new compounds belong to three structural types of natural products, including naphthopyrones (**2–5**), flavonoids (**6–7**), and bibenzyl (**8**). The *α*-glucosidase inhibitory activities of the isolated compounds were assayed. As a result, vittarin-B (**9**), rubrofusarin-6-*O*-*β*-D-glucopyranoside (**11**), quercetin (**14**), kaempferol (**15**), and dihydrokaempferol (**17**) showed moderate inhibitory activities against *α*-glucosidase with IC_50_ values of 22.5, 28.0, 36.5, 32.7, and 31.9 μM, respectively.

## Introduction

**FIGURE 1:**
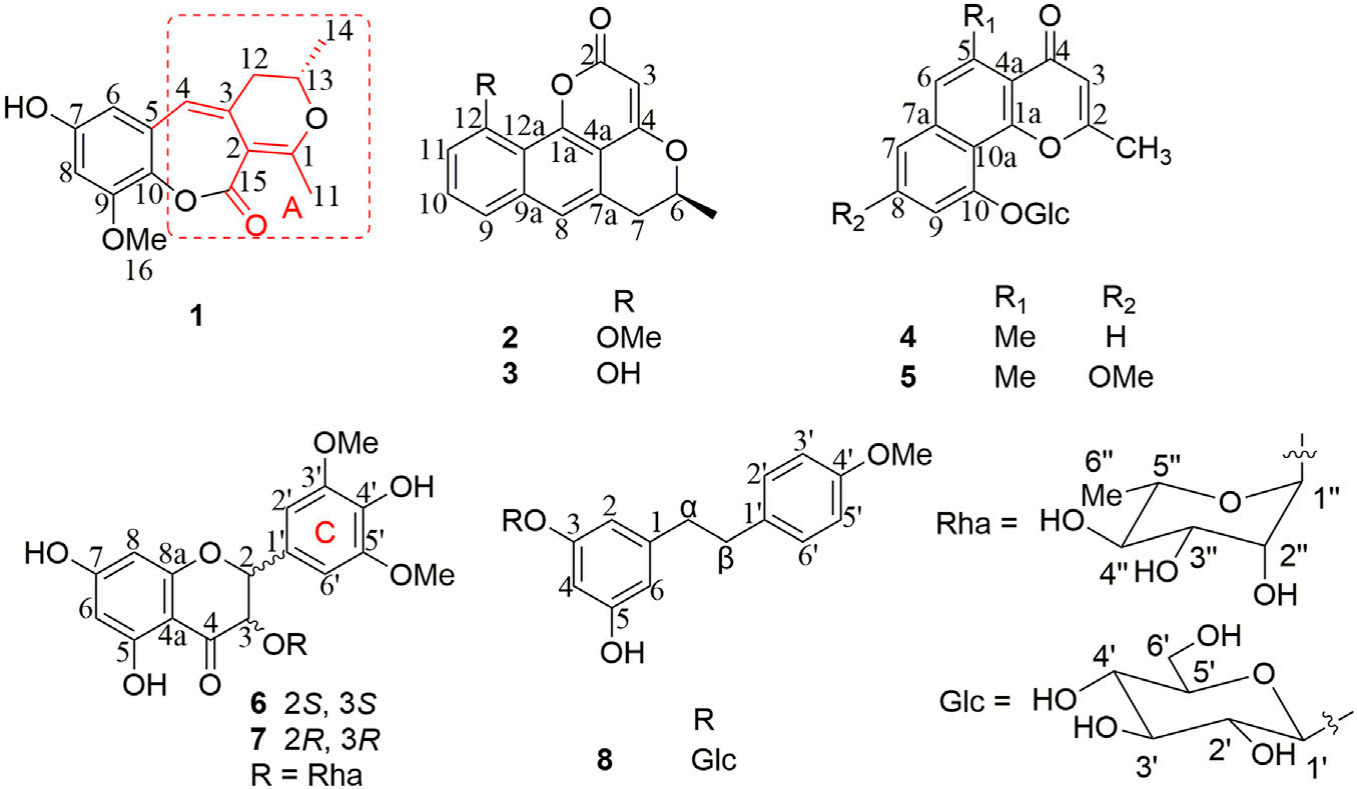
New phenolic compounds **1–8** from *Berchemia lineata* (L.) DC.

*Berchemia lineata* (L.) DC. belongs to the genus of *Berchemia*, defined as the plant origin of “Tiebaojin” in Guangxi Traditional Chinese Medicine Standard. “Tiebaojin” is an important ethnic medicine commonly used in Guangxi Zhuang nationality and southwest minority areas of China. It can be used for the treatment of pulmonary tuberculosis hemoptysis, icteric hepatitis, abdominal pain, traumatic injury, snake bite, etc. Through investigation, it is found that the actual source of the medicinal materials of “Tiebaojin” commonly used clinically in the Zhuang region of Guangxi mainly include *B. lineata*, *B. floribunda*, *B. polyphylla Wall. ex Laws.*, *B. polyphylla* var. *leioclada* (Zhang et al., 2011; Jing et al., 2017). Previous phytochemical studies on *B. floribunda* and *B. polyphylla* showed that they mainly include flavonoids, glycosides, lignans, quinones, and terpenoids (Chen et al., 2006). However, there are few studies on the chemical constituents of *B. lineata*, only some report chromones, flavonoids, and lignans (Shen et al., 2010a; Shen, et al., 2010b; Li et al., 2016; Jiang et al., 2019). To further search for new active compounds from *B. lineata*, phytochemical investigations of an extract of the stems and leaves of this plant afforded eight new phenolic compounds and eleven known analogues (Figure 1). This study reported the isolation, structure identification, and biological activity of these compounds.

## Materials and Methods

### General Experimental Procedures

The optical rotation was measured in MeOH by an Autopol IV polarimeter (Rudolph Research Analytical, Hackettstown, NJ, United States). UV spectra were obtained by a UH5300 UV–VIS double beam spectrophotometer (Hitachi Co., Tokyo, Japan). 1D and 2D NMR spectra were obtained by a Bruker AVANCE IIITM 500 and 600 MHz spectrometers (Bruker, Ettlingen, Germany) in methanol-*d*_*4*_, DMSO-*d*_*6*_ using TMS as internal standard. HR-ESI-MS data was obtained on a Thermo Scientific Q Exactive Orbitrap LC-MS/MS System (Thermo Scientific, Waltham, MA, United States). An Ultimate 3000 HPLC system (Dionex Co., Sunnyvale, CA, United States) with an UltiMate 3000 pump and UltiMate 3000 variable wavelength detector was employed to carry out semi-preparative HPLC, with a Nacalai Tesque 5C_18_-MS-II column (250 × 10 mm, 5 μm). Silica gel (200–300 mesh and 300–400 mesh) for open column chromatography (CC) was purchased from Qingdao Haiyang Chemical Group Co., Ltd. (Qingdao, China). The organic solvents were purchased from Sinopharm Chemical Reagent Co., Ltd. (Shanghai, China). Reagents used for *α*-glucosidase inhibitory assay (*α*-glucosidase, 4-nitrophenyl-*α*-D-glucopyranoside, and acarbose) were purchased from Shanghai Yuanye Biology Co., Ltd. (Shanghai, China), and the absorbance was measured by a full-wavelength microplate reader (Thermo Fisher Scientific Shier Technology Co., Ltd.).

### Plant Material

The stem and leaves of *Berchemia lineata* (L.) DC. were purchased from Nanning, Guangxi Zhuang autonomous Region, P. R. China, and were identified by Prof. Hongli Teng, Guangxi Zhuang Medicine International Hospital. The voucher specimen was deposited in the herbarium of the School of Pharmaceutical Sciences, South Central University for Nationalities.

### Extraction and Isolation

The stem and leaves of *Berchemia lineata* (L.) DC. (10 kg) were powdered and extracted using 70% EtOH three times for 24 h to obtain ethanol extract (700 g), which was then successively extracted with petroleum ether (PE), EtOAc, and *n*-butanol three times to obtain PE extract (57.31 g), EtOAc extract (100 g), and *n*-butanol extract (300 g). The *n*-butanol extract was chromatographed on a D-101 macroporous resin column, eluted successively with H_2_O-EtOH (7:3 to 1:9), and obtained 3 fractions (Fr.I-III). Fr.II (90 g) was chromatographed on a silica gel column, eluted successively with CH_2_Cl_2_-MeOH gradient (20:1, 9:1, 8:2, 7:3, 1:1) to obtain 12 fractions (Fr.II- A ∼ L). Fr.II-C was repeatedly prepared by semi-preparative HPLC to obtain **9** (CH_3_CN: H_2_O = 32: 68, *t*_*R*_ 50.1 min, 3 mg), and compound **12** (7 mg) was obtained from Fr.II-H by recrystallization. Fr.II-F was subjected to ODS column chromatography with a gradient of H_2_O-MeOH (7:3 to 0:1) to obtain 11 fractions (Fr.II-F.1∼F.11). Fr.II-F.2 was repeatedly prepared by semi-preparative HPLC (CH_3_CN: H_2_O = 19 : 81) to yield **6** (*t*_*R*_ 31.0 min, 1.5 mg) and **7** (*t*_*R*_ 34.9 min, 1.3 mg). Compounds **10** (4.5 mg) and **11** (4 mg) were obtained after recrystallization from Fr.II-F.3 and F.4, respectively. The mother liquid of Fr.II-F.3 was subjected to Sephadex LH-20 column chromatography eluting with methanol, and then repeatedly prepared using semi-preparative HPLC (CH_3_CN: H_2_O = 19 : 81) to obtain compounds **4** (1 mg, *t*_*R*_ 34.0 min), **5** (*t*_*R*_ 46.7 min, 1.1 mg), and **8** (*t*_*R*_ 25.6 min, 1 mg). Fr.II-J was subjected to ODS with a gradient of H_2_O-MeOH (7:3 to 0:1) and then repeatedly prepared by HPLC (CH_3_CN:H_2_O = 18:82) to obtain compound **13** (*t*_*R*_ 42.1 min, 3.89 mg). The EtOAc extract was chromatographed on a silica gel column chromatography, eluted successively with PE/EtOAc gradient (15:1 to 1:1) to obtain 16 fractions (Fr.1∼Fr.16), and compound 14 (4.5 mg) was obtained from Fr.15 by recrystallization. Fr.9 was subjected to a silica gel column chromatography, eluted successively with PE/EtOAc gradient (20:1 to 1:1), and then repeatedly prepared by semi-preparative HPLC to obtain **19** (CH_3_CN:H_2_O = 50:50, *t*_*R*_ 36.9 min, 4.9 mg). Fr.14 was subjected to ODS column chromatography with a gradient of H_2_O-MeOH (7:3 to 0:1) to obtain Fr.14.1∼Fr.14.14, and then repeatedly prepared by semi-preparative HPLC to obtain **1** (CH_3_CN:H_2_O = 30:70, *t*_*R*_ 67.2 min, 1 mg), **2** (CH_3_CN:H_2_O = 40:60, *t*_*R*_ 40.5 min, 4.5 mg), **3** (CH_3_CN:H_2_O = 40:60, *t*_*R*_ 33.5 min, 2 mg), **15** (CH_3_CN:H_2_O = 40:60, *t*_*R*_ 14.5 min, 12 mg), **16** (CH_3_CN:H_2_O = 35:65, *t*_*R*_ 21.7 min, 6 mg), **17** (CH_3_CN:H_2_O = 25:75, *t*_*R*_ 43.9 min, 3 mg), and **18** (CH_3_CN: H_2_O = 40:60, *t*_*R*_ 22.9 min, 3.2 mg).

### Spectroscopic Data

Bercheminol (**1**): brown amorphous powder; [*α*]^20^_D_ -27.3 (c 0.05, MeOH); UV (MeOH) *λ*_max_ (log *ε*) 210 (2.87), 295 (2.63) nm; ECD (MeOH) *λ* (θ) 227 (+14.58), 259 (+7.72), 307 (−11.78) nm; ^1^H NMR (500 MHz, CD_3_OD) *δ*_H_ 6.07 (1H, s, H-4), 6.15 (1H, d, *J* = 2.5 Hz, H-6), 6.40 (1H, d, *J* = 2.5 Hz, H-8), 2.31 (3H, s, CH_3_-11), 2.64 (1H, dd, *J* = 13.5, 3.5 Hz, H-12a), 2.35 (1H, dd, *J* = 13.5, 8.5, H-12b), 4.45 (1H, m, H-13), 1.34 (3H, d, *J* =6.5, CH_3_-14), 3.81 (3H, s, OMe); ^13^C NMR (125 MHz, CD_3_OD) *δ*_C_ 173.1 (C-1), 105.1 (C-2), 132.0 (C-3), 122.2 (C-4), 132.4 (C-5), 106.5 (C-6), 156.2 (C-7), 100.8 (C-8), 153.6 (C-9), 132.8 (C-10), 21.9 (C-11), 39.1 (C-12), 77.3 (C-13), 20.7 (C-14), 171.9 (C-15), 56.7 (OMe); HRESIMS *m/z* 289.10695 [M + H]^+^ (calcd for C_16_H_17_O_5_, 289.10705).

Bercheminol B (**2**): brown amorphous powder; [*α*]^20^_D_ +15.6 (c 0.05, MeOH); UV (MeOH) *λ*_max_ (log *ε*) 220 (2.87), 280 (2.61), 365 (2.10) nm; ECD (MeOH) *λ* (θ) 212 (+26.79), 228 (−14.00), 247 (+1.39), 286 (−5.44) nm; ^1^H and ^13^C NMR see Table 1; HRESIMS *m/z* 283.09594 [M + H]^+^ (calcd for C_17_H_15_O_4_, 283.09649).

**TABLE 1:** ^1^H NMR and^13^C NMR data of compounds **2-5** **(** *δ* in ppm, *J* in Hz).

No	^1^H-NMR			^13^C-NMR				
	2^a^	3^b^	4^a^	5^a^	2^a^	3^b^	4^a^	5^a^
1a					153.5	152.1	158.1	161.0
2					168.3	165.4	167.6	167.2
3	5.79 s	5.77 s	6.31 s	6.26 s	93.0	91.6	113.1	112.7
4					166.4	162.0	182.6	182.4
4a					108.6	106.0	120.7	118.9
5							136.5	137.1
6	4.59 m	4.61 m	7.50 s	7.40 s	76.7	74.6	127.7	127.0
7	3.03 dd (17.0, 12.0) 3.27 dd (17.0, 3.0)	3.00 dd (16.2, 10.8) 3.23 dd (16.2, 2.4)	7.50 d (7.8)	6.95 d (2.5)	35.2	33.4	122.2	101.7
7a					129.7	127.8	139.0	140.6
8	7.50 s	7.50 s	7.62 t (7.8)		123.0	121.1	131.4	162.7
9	7.44 d (8.2)	7.34 d (7.8)	7.34 d (7.8)	6.96 d (2.5)	121.3	118.2	112.1	103.9
9a					139.5	137.4		
10	7.58 t (8.2)	7.48 t (7.8)			131.3	130.2	156.4	157.8
10a							115.8	110.7
11	7.08 d (8.2)	7.00 d (7.8)			108.4	111.5		
12					159.2	155.7		
12a					114.9	112.2		
1′			5.26 d (7.5)	5.23 d (7.5)			102.3	102.2
2′			3.74 m	3.72 m			75.4	75.3
3′			3.53 m	3.53 m			78.5	78.5
4′			3.46 m	3.46 m			71.4	71.4
5′			3.55 m	3.53 m			78.7	78.7
6′			3.72 m 3.91 dd (2.5, 12.5)	3.72 m 3.91 m			62.6	62.6
6-Me	1.55 d (6.5)	1.47 d (6.6)			21.1	20.4		
12-OMe	4.07 s				56.6			
2-Me			2.55 s	2.53 s			20.1	20.1
5-Me			2.86 s	2.81 s			23.7	23.8
8-OMe				3.93 s				56.3

a NMR, data was obtained in CD_3_OD.

b NMR, data was obtained in DMSO-*d*_*6*_.

Bercheminol C (**3**): brown amorphous powder; [*α*]^20^_D_ +9.6 (c 0.05, MeOH); UV (MeOH) *λ*_max_ (log *ε*) 220 (2.64), 285 (2.35) nm; ECD (MeOH) *λ* (θ) 212 (+2.65), 231 (-1.96) nm; ^1^H and ^13^C NMR see Table 1; HRESIMS *m/z* 269.08072 [M + H]^+^ (calcd for C_16_H_13_O_4_, 269.08084).

Bercheminol D (**4**): white amorphous powder; [*α*]^20^_D_ -8.3 (c 0.02, MeOH); UV (MeOH) *λ*_max_ (log *ε*) 225 (3.11), 255 (2.98), 360 (2.37) nm; ^1^H and ^13^C NMR (MeOH) Table 1; HRESIMS *m/z* 403.13870 [M + H]^+^ (calcd for C_21_H_23_O_8_, 403.13874).

Bercheminol E (**5**): white amorphous powder; [*α*]^20^_D_ -1.1 (c 0.05, MeOH); UV (MeOH) *λ*_max_ (log *ε*) 235 (2.36), 270 (2.49), 350 (1.90) nm; ^1^H and ^13^C NMR see Table 1; HRESIMS *m/z* 433.14941 [M + H]^+^ (calcd for C_22_H_25_O_9_, 433.14931).

Bercheminol F (**6**): brown amorphous powder; [*α*]^20^_D_-65.0 (c 0.02, MeOH); UV (MeOH) *λ*_max_ (log *ε*) 230 (3.84), 290 (3.79) nm; ECD (MeOH) *λ* (θ) 207 (+8.93), 220 (-16.13), 245 (+0.41), 260 (-2.24), 294 (+10.27), 329 (-3.36) nm; ^1^H and ^13^C NMR see Table 2; HRESIMS *m/z* 493.13760 [M-H]^-^ (calcd for C_23_H_25_O_12_, 493.13515).

**TABLE 2:** ^1^H NMR,^13^C NMR, and HMBC data of compounds **6-7** in CD_3_OD (*δ* in ppm, *J* in Hz).

No	^1^H-NMR		^13^C-NMR		HMBC
	6	7	6	7	
2	5.05 d (11.5)	5.13 d (11.0)	84.2	84.5	C-3, C-2ʹ, C-1ʹ, C-4
3	4.72 d (11.5)	4.70 d (11.0)	77.1	78.5	C-2, C-1ʹʹ, C-1ʹ, C-4
4			197.8	196.4	
4a			102.2	102.2	
5			165.6	165.6	
6	5.92 s	5.91 s	96.6	96.7	
7			169.2	170.0	
8	5.91 s	5.90 s	96.6	96.7	
8a			164.4	164.2	
1′			129.4	128.6	
2′	6.83 s	6.85 s	106.5	106.2	C-2, C-1ʹ, C-4ʹ
3′			149.5	149.5	
4′			137.7	137.6	
5′			149.5	149.5	
6′	6.83 s	6.85 s	106.5	106.2	C-2, C-1ʹ, C-4ʹ
1″	5.18 s	3.99 s	103.0	102.3	C-3
2″	4.02 brs	3.52 m	72.1	72.1	
3″	3.40 dd (2.5, 9.5)	3.68 dd (3.5, 9.5)	72.0	72.3	
4″	3.19 t (9.5)	3.31 m	73.4	73.9	
5″	2.18 m	4.31 m	70.4	70.7	
6″	0.83 d (6.0)	1.20 d (6.5)	18.1	18.0	
3′,5′-OMe	3.90 s	3.88 s	57.1	57.1	C-3ʹ, 5ʹ

Bercheminol G (**7**): brown amorphous powder; [*α*]^20^_D_ +17.8 (c 0.02, MeOH); UV (MeOH) *λ*_max_ (log *ε*) 230 (3.81), 295 (3.70) nm; ECD (MeOH) *λ* (θ) 208 (-7.38), 229 (+9.17), 295 (-4.61), 321 (+2.54) nm; ^1^H and ^13^C NMR see Table 2; HRESIMS *m/z* 493.13589 [M-H]^-^ (calcd for C_23_H_25_O_12_, 493.13515).

Bercheminol H (**8**): yellow amorphous powder; [*α*]^20^_D_ +6.0 (c 0.05, MeOH); UV (MeOH) *λ*_max_ (log *ε*) 210 (3.04), 275 (2.19) nm; ^1^H NMR (500 MHz, CD_3_OD) *δ*_H_ 6.39 (1H, br s, H-2), 6.37 (1H, t, *J* = 2.0 Hz, H-4), 6.29 (1H, br s, H-6), 7.06 (2H, d, *J* = 8.5 Hz, H-2′, 6′), 6.79 d (2H, d, *J* = 8.5 Hz, H-3′, 5′), 4.78 (1H, d, *J* = 7.5 Hz, H-1″), 3.40 (2H, m, H-2″, 6″), 3.41 (2H, m, H-3″, 5″), 3.38 (1H, m, H-4″), 3.88 (1H, d, *J* = 12.5 Hz, H_2_-6″a), 3.69 (1H, dd, *J* =5.0, 12.0, H_2_-6″b), 2.76 (4H, m H_2_-*α*, H_2_-*β*); ^13^C NMR (125 MHz, CD_3_OD) *δ*_C_ 145.6 (C-1), 110.9 (C-2), 159.4 (C-3), 102.8 (C-4), 160.3 (C-5), 109.4 (C-6), 135.2 (C-1′), 130.6 (C-2′), 114.8 (C-3′), 159.4 (C-4′), 114.8 (C-5′), 130.6 (C-6′), 102.4 (C-1″), 75.0 (C-2″), 78.2 (C-3″), 71.5 (C-4″), 78.2 (C-5″), 62.6 (C-6″), 39.6 (C-*α*), 37.0 (C-*β*), 55.8 (OMe); HRESIMS *m/z* 407.17007 [M + H]^+^ (calcd for C_21_H_27_O_8_, 407.17004).

### *α*-Glucosidase Inhibitory Assay

According to the literature method (Özgünseven et al., 2021) with some modifications, 20 μL compounds at different concentrations reacted with 20 μL of 4-nitrophenyl-*α*-D-glucopyranoside (20 mM) and 20 μL of *α*-glucosidase (0.5 U/mL) in a 96-well plate at 37°C for 30 min. Na_2_CO_3_ (0.2%, 80 μL) was then added to terminate the reaction and the absorbance value was measured at 405 nm using a microplate reader. IC_50_ values were calculated from the graph of inhibition percentage against the logarithm of the concentrations of compounds.

### Determination of Sugar

Compounds **4, 5, 6, 7**, and **8** (0.5 mg)were refluxed with 2 ml of 4N HCl–dioxane (1 : 1) for 2 h, and then cooled to room temperature. The mixtures were extracted three times with 2 ml EtOAc. The aqueous layers were dried and refluxed with 0.5 ml pyridine and 1 mg L-cysteine methyl ester hydrochloride at 60 C for 1 h. 5 μL 2-methylphenyl isothiocyanate was added to the reaction mixtures and continued to reflux at 60 C for 1 h (Min et al., 2003; Tanaka, T., 2007). These dithiocarbamate derivatives of D-glucose and L-rhamnose were prepared in the same way. The reaction mixtures were analyzed by HPLC (column: YMC-Pack ODS-A 250 × 4.6 mm I.D.; CH_3_CN/H_2_O = 25: 75, wavelength: 250 nm; flow rate: 0.8 ml/min). The retention time of three sugar fractions of compounds **4**, **5**, and **8** were detected at 22.56, 22.40, and 22.43 min respectively, which were almost the same as that of D-glucose (22.61 min). The retention time of two sugar fractions of compounds **6** and **7** were detected at 25.52 and 25.49 min, respectively, which were almost the same as that of L-rhamnose (25.56 min). The results showed that compounds **4, 5,** and **8** contained D-glucose, and compounds 6 and 7 contained L-rhamnose.

## Results and Discussion

**FIGURE 2:**
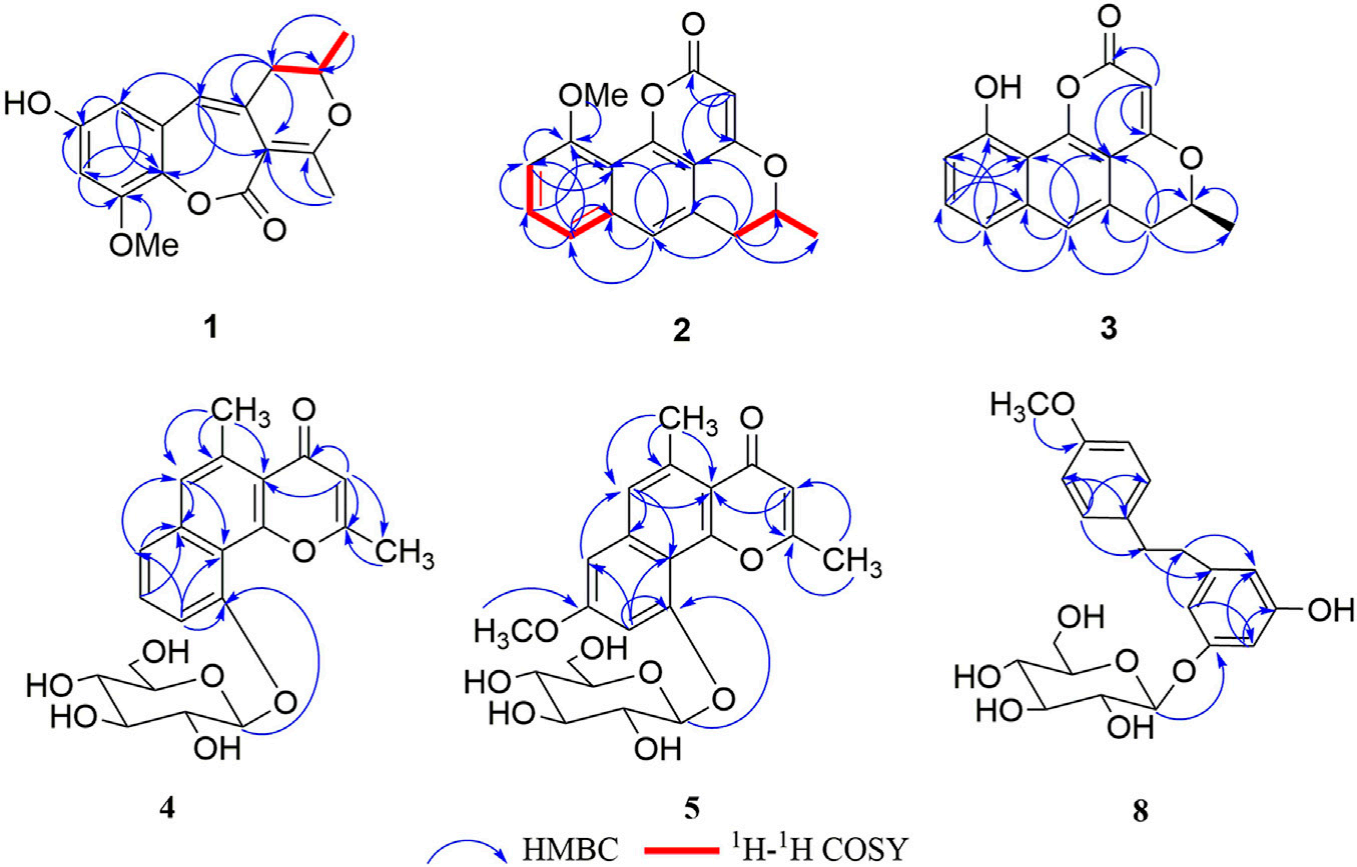
Key HMBC for compounds **1–5**, and **8**, and ^1^H–^1^H COSY correlations for **1** and **2**.

**FIGURE 3:**
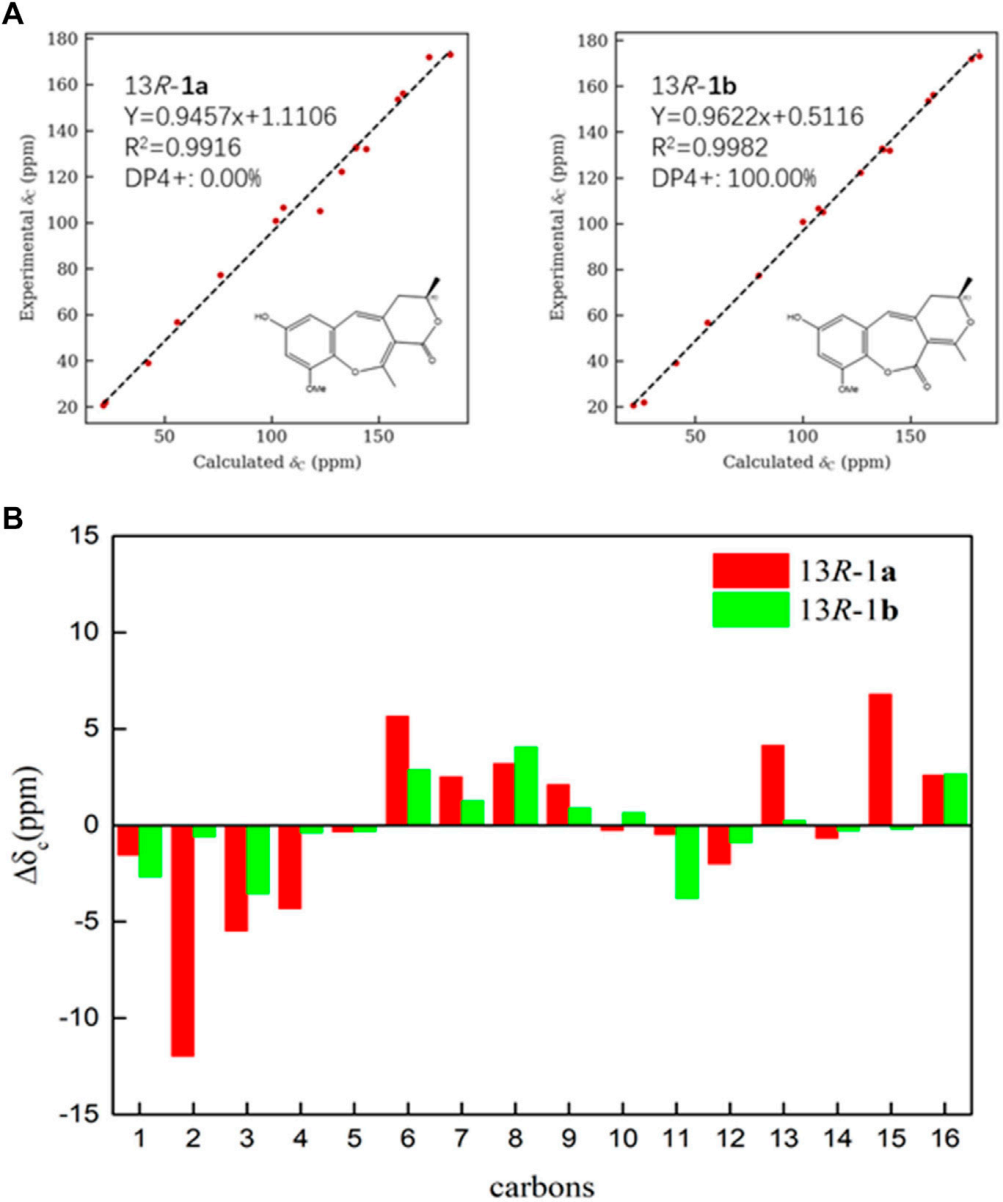
**(A)** Linear regression fitting of calculated ^13^C chemical shifts of two possible isomers of **1a** and **1b** with the experimental values **(B)** Deviation between calculated ^13^C chemical shifts of two possible isomers of **1a** and **1b** with the experimental values.

**FIGURE 4:**
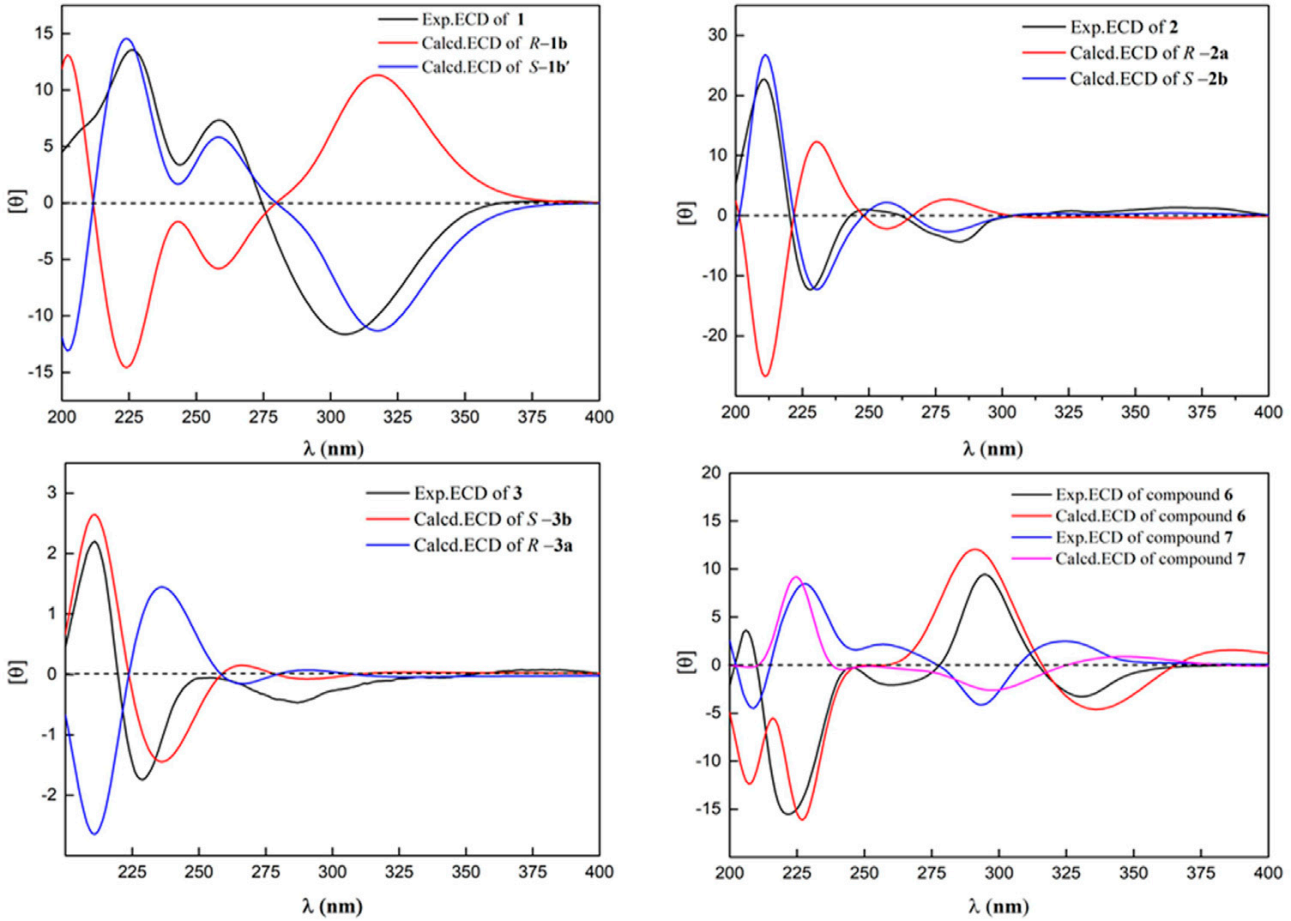
Calculated and experimental ECD spectra of **1–3** and **6–7**.

**SCHEME 1:**
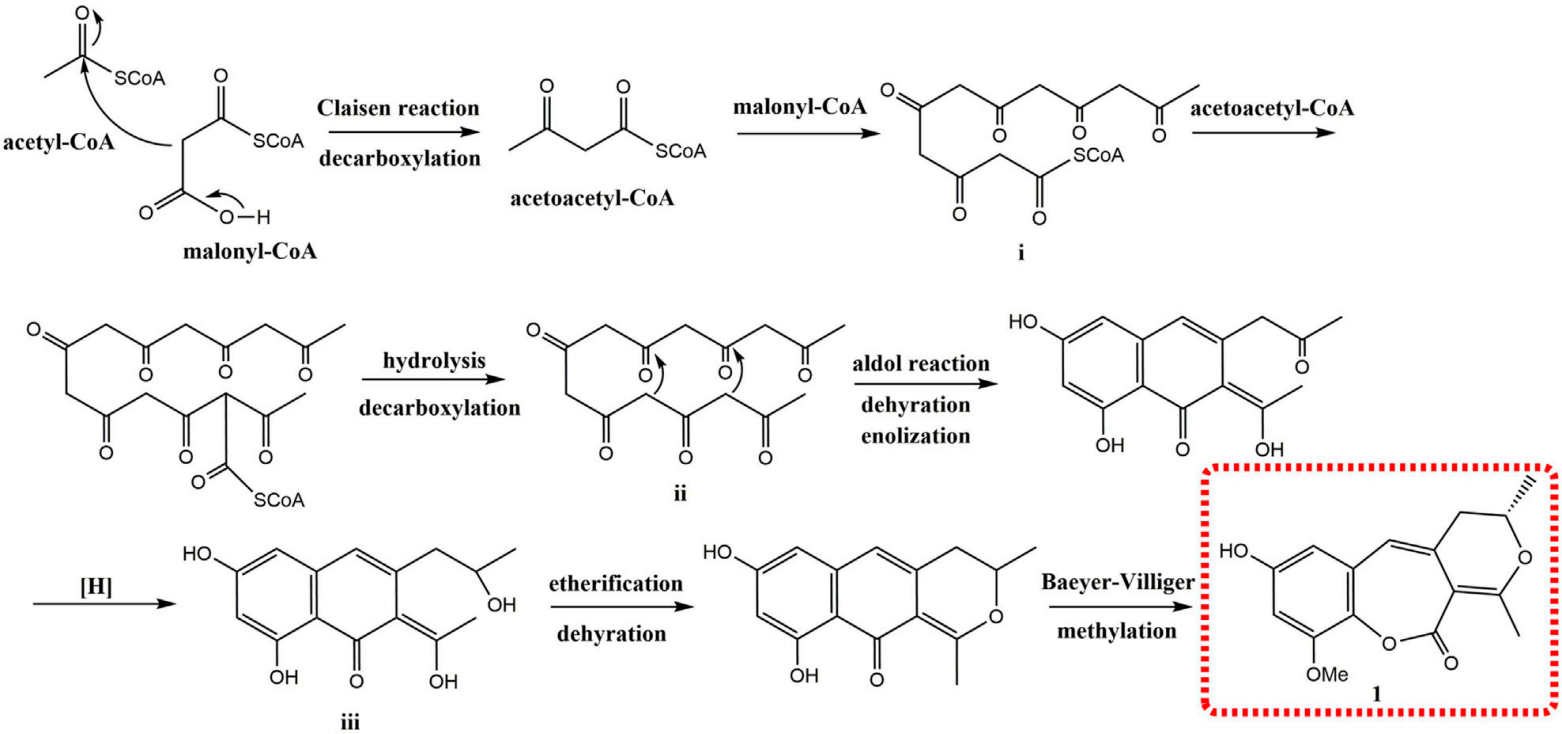
Plausible biosynthetic pathway for **1**.

Compound **1** was obtained as a brown amorphous powder. According to the *pseudo* molecular ion peak at *m/z* 289.10695 [M + H]^+^ (calcd 289.10705) of HR-ESI-MS, the molecular formula was determined as C_16_H_16_O_5_, suggesting nine degrees of unsaturation (DOUs). The ^1^H-NMR spectrum of **1** indicated that **1** contained a pair of *meta*-coupled aromatic proton signals [*δ*_H_ 6.15 (1H, d, *J* = 2.5 Hz, H-6), 6.40 (1H, d, *J* = 2.5 Hz, H-8)], an isolated olefin proton signal [*δ*_H_ 6.07 (1H, s, H-4)], a pair of methylene signals [*δ*_H_ 2.64 (1H, dd, *J* = 13.5, 3.5 Hz), 2.35 (1H, dd, *J* = 13.5, 8.5 Hz), an oxygenated methine [*δ*_H_ 4.45 (1H, m)], two methyls [*δ*_H_ 2.31 (3H, s), 1.34 (3H, d, *J* = 6.5 Hz)], and a methoxy [*δ*_H_ 3.81 (3H, s)]. The ^13^C-NMR spectra that combined with HSQC and HMBC spectrum indicated the presence of 16 carbon signals, including one 1, 2, 3, 5-tetrasubstituted phenyl group [*δ*_C_ 132.4 (s), 106.5 (d), 156.2 (s), 100.8 (d), 153.6 (s), 132.8 (s)], one trisubstituted double bond [*δ*_C_ 122.2 (d), 132.0 (s)], an enolized double bond [*δ*_C_ 173.1 (s), 105.1 (s)], one ester carbonyl carbon [*δ*_C_ 171.9 (s)], an oxygenated methine [*δ*_C_ 77.3 (d)], two methyls [*δ*_C_ 21.9 (q), 20.7 (q)], one methoxy [*δ*_C_ 56.7 (q)], and one methylene [*δ*_C_ 39.1 (t)]. According to ^1^H–^1^H COSY and HSQC spectra, the structural fragment CH_3_(14)CH(O) (13)CH_2_(12)- was deduced. HMBC correlations (Figure 2) from H_2_-12 to *δ*_*C*_ 105.1 (s, C-2), 132.0 (s, C-3) and 122.2 (s, C-4), from H-4 to *δ*_*C*_ 105.1 (s, C-2), from CH_3_-11 [*δ*_*H*_ 2.31 (s)] to *δ*_*C*_ 105.1 (s, C-2), and *δ*_*C*_ 173.1 (s, C-1) suggested that the trisubstituted double bond was connected to C-12, and the trisubstituted and enolized double bonds were connected through C-3 to C-2. The remaining ester carbonyl carbon should be connected to C-2. Therefore, substructure A was established as depicted in Figure 1. Furthermore, HMBC correlations from H-4 to *δ*_*C*_ 106.5 (d, C-6) and 132.8 (s, C-10) suggested that substructure A was connected to C-5. ROESY correlation of MeO to H-8 and HMBC correlation of MeO to *δ*_*C*_ 153.6 (s, C-9) indicated that the methoxy group was connected to C-9. Except for the presence of two double bonds, one carbonyl and one phenyl, two DOUs were needed to satisfy the molecular formula of **1**. Therefore, compound **1** still had two more rings. Owing to insufficient HMBC correlations, the connection positions of the two rings could not be determined, which ring was formed through ether bond, and which ring was formed by an ester bond. Therefore, there were two possible structures for **1**. The ether bond was formed between C-1 and C-10, and C-2 was connected to C-13 through the carbonyl C-15 to form the ester bond in the candidate structure **1a**. In contrast, C-2 was connected to C-10 through the carbonyl C-15 to form the ester bond, and the ether bond was formed between C-1 and C-13 in the candidate structure **1b**. To further assign the connection positions of the two rings, NMR calculations with DP4+ analysis for two possible isomers were carried out. As a result, **1b** was the most likely structure based on DP4+ probability with 100% (Figure 3). Ultimately, its absolute configuration was confirmed as (13*S*) by ECD calculations (Figure 4). Therefore, the structure of **1** was defined and named bercheminol A, which possessed an unprecedented 3, 4-Dihydro-11H-benzo [b]pyrano [4,3-e] oxepin-11-one skeleton. From the biogenic analysis, compound **1** comes from the acetate-malonate (AA-MA) pathway. The reasonable biosynthetic pathway is shown in Scheme 1. First, one acetyl coenzyme A (CoA) and five malonyl-CoA undergo Claisen condensation and decarboxylation to obtain polyketone i. Then, i react with acetoacetyl-CoA through a series of reactions, including Claisen condensation, hydrolysis and decarboxylation to obtain the key intermediate ii. Starting from ii, phenolic compound iii is obtained through aldol condensation, dehydration, enolization, and reduction. Finally, iii is etherified, oxidized, and methylated to give compound **1**.

Compound **2** was obtained as a brown amorphous powder. The molecular formula was determined as C_17_H_14_O_4_ based on the HR-ESI-MS of the protonated molecular ion peak at *m/z* 283.09594 [M + H]^+^ (calcd 283.09649), suggesting 11 DOU. The ^1^H-NMR spectrum of **2** indicated the presence of a set of 1, 2, 3-trisubstituted benzene ring [*δ*_H_ 7.44 (1H, d, *J* =8.2 Hz, H-9), 7.58 (1H, t, *J* =8.2 Hz, H-10), 7.08(1H, d, *J* =8.2 Hz, H-11)], an isolated aromatic proton signal [*δ*_H_ 7.50 (1H, s, H-8)], an olefin proton signal [*δ*_H_ 5.79 (1H, s, H-3)], a pair of double doublets [*δ*_H_ 3.03 (1H, dd, *J* =12.0, 17.0 Hz, H-7), 3.27 (1H, dd, *J* =3.0, 17.0 Hz, H-7), and an oxygenated methine [*δ*_H_ 4.59 (1H, m, H-6)]. The ^13^C-NMR and DEPT spectra exhibited 17 carbon signals, including 5 *sp^2^* methines, 1 methyl, 1 methylene, 1 methoxy, and 4 *sp^2^* quaternary carbons, 3 oxygenated *sp^2^* tertiary carbons, one oxygenated methine, and one ester carbonyl carbon. The above NMR data suggested that **2** might belong to naphthopyranone. By comparison of the NMR data of **2** with those of pannorin B (Kaur et al., 2015) suggested that their structures closely resembled except for two major differences. First, the signal of 1, 2, 3-trisubstituted benzene ring in **2** replaced a pair of *meta*-coupled aromatic protons in pannorin B, suggesting that the methoxy and hydroxyl groups at C-10 and C-12 in the latter were replaced by the hydrogen and methoxy group, respectively, which was further supported by HMBC spectrum. Second, the chemical shifts of C-6 have shifted upfield 24.7 ppm, respectively, compared to pannorin B, suggesting that the hemiacetal carbon at C-6 in pannorin B was replaced by an oxygenated methine which was further confirmed by ^1^H–^1^H COSY correlation of CH_3_CH(O)CH_2_- and HMBC spectrum. Ultimately, its absolute configuration was established as (6*S*) by ECD calculations (Figure 4). Therefore, the structure of **2** was defined and named bercheminol B.

Compound **3** was obtained as a brown amorphous powder. The molecular formula was determined as C_16_H_12_O_4_ by the HR-ESI-MS of the protonated molecular ion peak at *m/z*269.08072 [M + H]^+^ (calcd 269.08084), suggesting 14 mass units less than **2**. Compared with NMR data of **2, 3** had one less methoxy signal, suggesting that **3** was demethylated of **2** which was further confirmed by HMBC spectrum and ECD calculations. Thus, the structure of **3** was defined and named bercheminol C.

Compound **4** was obtained as a white amorphous powder. The molecular formula was determined as C_21_H_22_O_8_ by the HR-ESI-MS of the protonated molecular ion peak at *m/z* 403.13870 [M + H]^+^ (calcd 403.13874). Acid hydrolysis of **4** and HPLC analysis of dithiocarbamate derivative of sugar provided D-glucose. The configuration of the D-glucose was determined *β* by the coupling constant of 7.5 Hz of an anomeric proton (Cai et al., 2021). In addition to the NMR signal of glucose, the ^1^H-NMR spectrum of the aglycone of **4** contained an isolated aromatic proton signal [*δ*_H_ 7.50(1H, s, H-6)], a set of signals of 1, 2, 3-trisubstituted benzene ring [*δ*_H_ 7.62 (1H, t, *J* = 7.8 Hz, H-8), 7.34 (1H, d, *J* = 7.8 Hz, H-9), 7.50 (1H, d, *J* = 7.8 Hz, H-7)], an olefinic proton [*δ*_H_ 6.31 (1H, s, H-3)], and two tertiary methyls [*δ*_H_ 2.55, 2.86 (each 3H, s)]. The ^13^C-NMR and DEPT spectra of the aglycone of **4** revealed the presence of five *sp^2^* methines, two methyls, and eight non-protonated carbons, including four *sp^2^* quaternary carbons, a conjugated carbonyl carbon [*δ*_C_ 182.6 (C-4)], three oxygenated tertiary carbons [*δ*_C_ 167.6 (C-2), 158.1(C-1a), 156.4 (C-10)]. The above NMR data suggested that **4** might belong to angular naphthopyrone (Shen et al., 2007; Chovolou et al., 2011). By careful comparison of NMR data of **4** with those of pleuropyrone A, their structures resembled each other. The major difference between them was the presence of signals of a 1, 2, 3-trisubstituted benzene ring in **4**, instead of a pair of *meta*-coupled aromatic proton signals in pleuropyrone A (Min et al., 2003). This deduction was further confirmed by HMBC correlations and H-7/H-8/H-9 of ^1^H–^1^H COSY. Therefore, the structure of **4** was defined and named bercheminol D.

Compound **5** was obtained as a white amorphous powder. The molecular formula was determined as C_22_H_24_O_9_ by the HR-ESI-MS of the protonated molecular ion peak at *m/z* 433.14941 [M + H]^+^ (calcd 433.14931) suggesting 14 mass units more than pleuropyrone A. By careful comparison of NMR data of **5** with those of pleuropyrone A, it was found that the only difference was the presence of an additional methoxy signal. It was suggested that **5** was an 8-*O*-methylated derivative of pleuropyrone A, which was further confirmed by the HMBC correlation from the methoxy group to C-8. Therefore, the structure of **5** was defined and named bercheminol E.

Compounds **6** and **7** were obtained as brown amorphous powders. The negative mode of HR-ESI-MS of **6** and **7** showed their quasimolecular ions at *m/z* 493.13760 and 493.13589 [M-H]^-^ respectively, suggesting the molecular formula C_23_H_26_O_12_. The detailed analyses of their NMR data indicated that they also possessed identical planar structures and belonged to dihydroflavonol glycoside containing L-rhamnopyranosyl moiety, which was further supported by acid hydrolysis of **6** and **7**. By comparison of the NMR data of **6** and **7** with those of neoastilbin (De Britto et al., 1995), it was found that they are different from neoastilbin in the substituent pattern of the C ring. The ring C of 3,4-dihydroxyphenyl in neoastilbin was replaced by 3, 5-dimethoxy-4-hydroxyphenyl in **6** and **7**. HMBC correlations from H-2′ and 6′ to C-2, C-1′ and C-4′, and MeO to C-3′ and C-5′ confirmed these findings. Careful analyses of the NMR data of **6** and **7** indicated that the difference between **6** and **7** were the absolute configurations of C-2 and C-3, which were the same as those of neoastilbin and astilbin. Compared to a (2*R*, 3*R*) configuration, the chemical shifts of H-1″ and H-2″ of the *O*-rhamnopyranosyl at C-3 in the (2*S*, 3*S*) configuration were located downfield, the chemical shifts of H-5″ and CH_3_-6″ were located upfield (De Britto et al., 1995). According to this rule, the absolute configurations of C-2 and C-3 of **6** and **7** were determined as (2*S*, 3*S*) and (2*R*, 3*R*)*,* respectively. The presence of negative and positive cotton effect at about 330 nm in the CD spectrum of **6** and **7**, respectively, and ECD calculations confirmed these findings (Figure 4). Therefore, the structures of **6** and **7** were defined and named as bercheminols F and G.

Compound **8** was obtained as a yellow amorphous powder. The molecular formula was determined as C_21_H_26_O_8_ by the HR-ESI-MS of the protonated molecular ion peak at *m/z* 407.17007 (calcd 407.17004). Acid hydrolysis of **8** and HPLC analysis of dithiocarbamate derivative of sugar provided D-glucose. The configuration of D-glucose was determined as *β* based on the coupling constant of 7.5 Hz of an anomeric proton. Detailed analyses of its NMR data indicated that the aglycone of **8** was determined as vittarin A (Wu et al., 2005). Therefore, **8** was 3-*O*-glucosylated derivative of vittarin A, which was further confirmed by the HMBC correlation from the anomeric proton of glycopyranosyl to C-3. Therefore, the structure of **8** was defined and named bercheminol H.

By comparing the spectral data of these compounds with those reported in the literature, the structures of 11 known compounds are identified as vittarin-B (**9**) (Wu et al., 2005), demethylflavasperone-10-*O*-*β*-D-glucopyranoside (**10**) (Xiong et al., 2019), rubrofusarin-6-*O*-*β*-D-glucopyranoside (**11**) (Messana et al., 1991), rubrofusarin-6-*O*-*α*-L-rhamnosyl-(1→6)-*O*-*β*-D-glucopyranside (**12**) (Shen et al., 2007), kaempferol-3-*O*-*α*-L-rhamnopyranosyl-(1→6)-*β*-D-glucopyranoside (**13**) (Thissera et al., 2020), quercetin (**14**) (Xiong et al., 2019), kaempferol (**15**) (Li et al., 2016), naringenin (**16**) (Gao et al., 2005), dihydrokaempferol (**17**) (Lu et al., 2015), 2-hydroxyemodin 1-methyl ether (**18**) (Lin et al., 2001), emodin (**19**) (Cohen et al., 1995).

The study of the chemical constituents of this plant led to the isolation of five types of phenolic compounds. Compound **1** possesses an unprecedented carbon skeleton with 3, 4-dihydro-11H-benzo [b]pyrano [4,3-e]oxepin-11-one. In addition to this new skeleton, four known types of phenolic compounds were also isolated, including naphthopyrones (**2-5, 10-12**), flavonoids (**6-7, 13-17**), bibenzyls (**8-9**), and anthraquinones (**18–19**). Furthermore, naphthopyrones may be categorized into two types: naphtho-α-pyrones, such as **2-3**, and naphtho-γ-pyrones, such as **4-5**, **10** which belonged to angular ones, and **11-12** which belonged to linear ones. Because of the insufficient amount of compounds **1** and **4–8**, their biological activities were not tested. The *α*-glucosidase inhibitory activities of the remaining compounds were assayed (Table 3). As a result, vittarin-B (**9**), rubrofusarin-6-*O*-*β*-D-glucopyranoside (**11**), quercetin (**14**), kaempferol (**15**), and dihydrokaempferol (**17**) showed moderateα-glucosidase inhibitory activities with IC_50_ values of 22.5, 28.0, 36.5, 32.7, and 31.9 μM, respectively.

**TABLE 3:** *α*-Glucosidase inhibitory activities of compounds **1–19**.

No	IC_50_ (μM)	No	IC_50_ (μM)
1	NT	11	28.0
2	NA	12	NA
3	NA	13	NA
4	NT	14	36.5
5	NT	15	32.7
6	NT	16	NA
7	NT	17	31.9
8	NT	18	NA
9	22.5	19	NA
10	NA	Acarbose	0.04

NA: no activity (IC_50_ > 50 μM); NT: not tested.

## Conclusion

In conclusion, 19 structurally diverse phenolic compounds were isolated from the stem and leaves of *Berchemia lineata* (L.) DC., among which compounds **1–8** were previously undescribed. Most of isolated compounds were evaluated for their *α*-glucosidase inhibitory activities. As a result, bibenzyl (**9**), linear naphtho-γ-pyrone (**11**), and flavonoids (**14–15, 17**) displayed moderate inhibitory activities against *α*-glucosidase. Therefore, those compounds might be accountable for the antihyperglycemic effect of this herb.

## Data Availability

The original contributions presented in the study are included in the article/Supplementary Material, further inquiries can be directed to the corresponding authors.
