# Supplementary material for: Phenolic Compounds From the Stems and Leaves of Berchemia lineata (L.) DC
Source: Front Chem. 2022 Apr 14;10:889441. doi: 10.3389/fchem.2022.889441 (PMC9047694; doi:10.3389/fchem.2022.889441)
Supplement: Supplementary file 1 [file DataSheet1.PDF]

Phenolic Compounds from the Stems and Leaves of *Berchemia lineata* (L.) DC.

Yitong Li[a,1], *Yu Chen*[b,1], *Wenli Xie*[a], *Xueni Li*[a], *Gui Mei*[a], *Jing Xu*[a], *Xiangpei Zhao*[c], *Hongli Teng*[c*], *Guangzhong Yang*[a*]

[a] *School of Pharmaceutical Sciences, South-Central University for Nationalities, Wuhan 430074, P. R. China*

[b] *College of Chemistry and Material Sciences, South-Central University for Nationalities, Wuhan 430074, P. R. China*

[c] *Guangxi International Zhuang Medical Hospital, Nanning 530201, P. R. China*

*Corresponding authors. Tel./fax: +86 27 6784 1196

E-mail address: 564988177@qq.com (H. Teng); yanggz888@126.com (G. Yang)

[1] These authors contributed equally to this work.

Contents

Figure S1: ^{1}H-NMR (500 MHz, CD_3OD) spectrum of compound 1 5

Figure S2: ^{13}C-NMR (125 MHz, CD_3OD) spectrum of compound 1 5

Figure S3: ^{13}C-NMR-DEPT (θ=135°) spectrum of compound 1 6

Figure S4: HSQC spectrum of compound 1 6

Figure S5: HMBC spectrum of compound 1 7

Figure S6: ^{1}H-^{1}H COSY spectrum of compound 1 7

Figure S7: ROESY spectrum of compound 1 8

Figure S8: UV spectrum of compound 1 8

Figure S9: HR-ESI-MS of compound 1 9

Figure S10: CD spectrum of compound 1 9

Figure S11: ^{1}H-NMR (500 MHz, CD_3OD) spectrum of compound 2 10

Figure S12: ^{13}C-NMR (125 MHz, CD_3OD) spectrum of compound 2 10

Figure S13: 13C-NMR-DEPT (θ=135°) spectrum of compound 2 11

Figure S14: HSQC spectrum of compound 2 11

Figure S15: HMBC spectrum of compound 2 12

Figure S16: ^{1}H-^{1}H COSY spectrum of compound 2 12

Figure S17: ROESY spectrum of compound 2 13

Figure S18: UV spectrum of compound 2 13

Figure S19: HR-ESI-MS of compound 2 14

Figure S20: CD spectrum of compound 2 14

Figure S21: ^{1}H-NMR (600 MHz, DMSO) spectrum of compound 3 15

Figure S22: The ^{13}C-NMR (150 MHz, DMSO) spectrum of compound 3 15

Figure S23: ^{13}C-NMR-DEPT (θ=135°) spectrum of compound 3 16

Figure S24: HSQC spectrum of compound 3 16

Figure S25: HMBC spectrum of compound 3 17

Figure S26: UV spectrum of compound 3 17

Figure S27: HR-ESI-MS of compound 3 18
Figure S28: CD spectrum of compound 3..18
Figure S29: ^{1}H-NMR (500 MHz, CD_3OD) spectrum of compound 4..19
Figure S30: The ^{13}C-NMR (125 MHz, CD_3OD) spectrum of compound 4..19
Figure S31: ^{13}C-NMR-DEPT (θ=135°) spectrum of compound 4..20
Figure S32: HSQC spectrum of compound 4..20
Figure S33: HMBC spectrum of compound 4..21
Figure S34: UV spectrum of compound 4..21
Figure S35: HR-ESI-MS of compound 4..22
Figure S36: ^{1}H-NMR (500 MHz, CD_3OD) spectrum of compound 5..22
Figure S37: ^{13}C-NMR (125 MHz, CD_3OD) spectrum of compound 5..23
Figure S38: ^{13}C-NMR-DEPT (θ=135°) spectrum of compound 5..23
Figure S39: HSQC spectrum of compound 5..24
Figure S40: HMBC spectrum of compound 5..24
Figure S41: UV spectrum of compound 5..25
Figure S42: HR-ESI-MS of compound 5..25
Figure S43: ^{1}H-NMR (500 MHz, CD_3OD) spectrum of compound 6..26
Figure S44: ^{13}C-NMR (125 MHz, CD_3OD) spectrum of compound 6..26
Figure S45: ^{13}C-NMR-DEPT (θ=135°) spectrum of compound 6..27
Figure S46: HSQC spectrum of compound 6..27
Figure S47: HMBC spectrum of compound 6..28
Figure S48: UV spectrum of compound 6..28
Figure S49: HR-ESI-MS of compound 6..29
Figure S50: CD spectrum of compound 6..29
Figure S51: ^{1}H-NMR (500 MHz, CD_3OD) spectrum of compound 7..30
Figure S52: The ^{13}C-NMR (125 MHz, CD_3OD) spectrum of compound 7..30
Figure S53: ^{13}C-NMR-DEPT (θ=135°) spectrum of compound 7..31

Figure S54: HSQC spectrum of compound 7 31

Figure S55: HMBC spectrum of compound 7 32

Figure S56: UV spectrum of compound 7...32

Figure S57: HR-ESI-MS of compound 7 33

Figure S58: CD spectrum of compound 7...33

Figure S59: ^{1}H-NMR (500 MHz, CD_3OD) spectrum of compound 8 34

Figure S60: ^{13}C-NMR (125 MHz, CD_3OD) spectrum of compound 8......................................34

Figure S61: ^{13}C-NMR-DEPT (θ=135°) spectrum of compound 8...35

Figure S62: HSQC spectrum of compound 8 35

Figure S63: HMBC spectrum of compound 8 36

Figure S64: UV spectrum of compound 8...36

Figure S65: HR-ESI-MS of compound 8 37

Figure S66 Phenolic compounds from *Berchemia lineata* (L.) DC. 38

Figure S67 Chemical structure of compound 1..39

Figure S68 Chemical structure of compound 2..40

Figure S69 Chemical structure of compound 3..40

Figure S70 Chemical structure of compound 6 and 7...40

1.Energies and Coordinates 42

2.Experimental and Computed NMR Chemical Shifts...69

Figure S71 DP4+ probability for compounds 1a and 1b based on its ^{1}H and ^{13}C-NMR date. 71

Figure S72 Calculated ECD spectra of compounds 1 was compared with the experimental.72

Figure S73 Calculated ECD spectra of compounds 2 was compared with the experimental.72

Figure S74Calculated ECD spectra of compounds 3 was compared with the experimental..73

Figure S75 Calculated ECD spectra of compounds 6 and 7 was compared with the experimental. 73

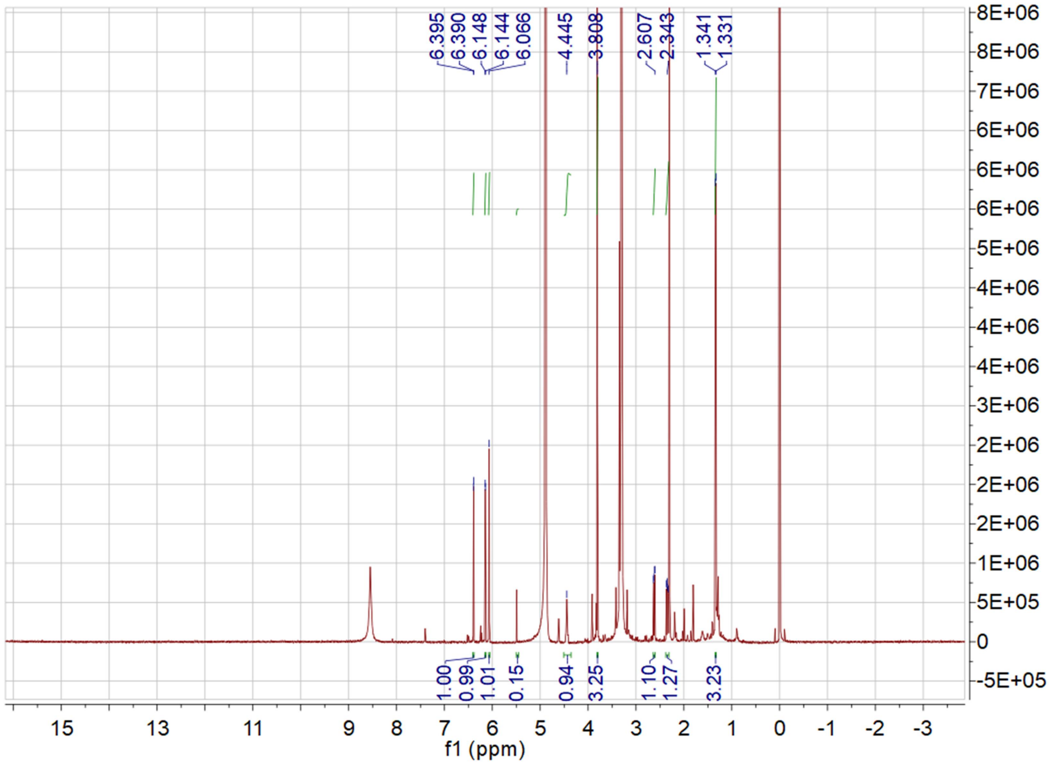

Figure S1: ^{1}H-NMR (500 MHz, CD_3OD) spectrum of compound 1

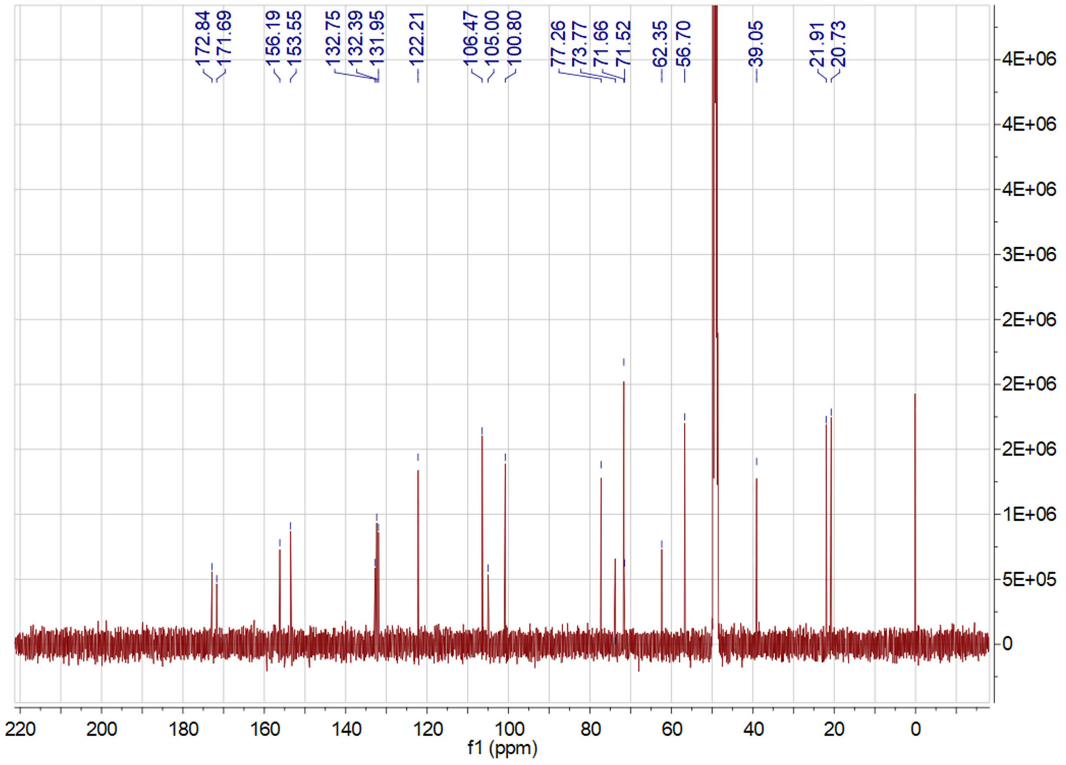

Figure S2: ^{13}C-NMR (125 MHz, CD_3OD) spectrum of compound 1

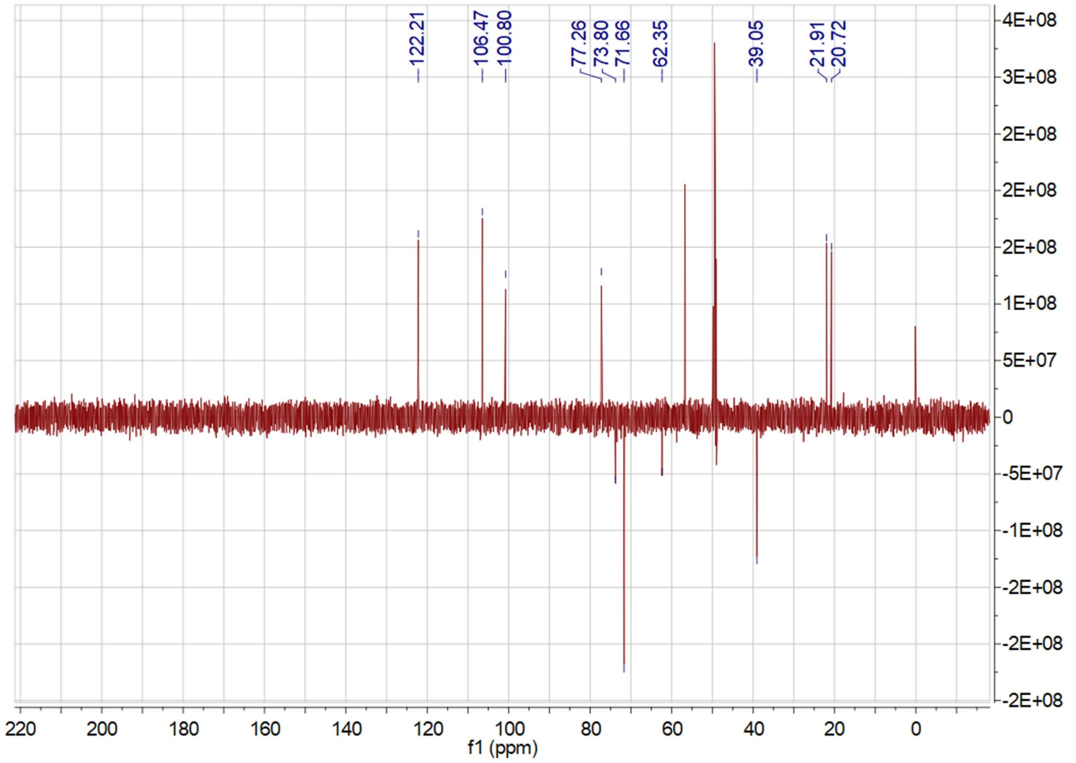

Figure S3: ^{13}C-NMR-DEPT (θ=135°) spectrum of compound 1

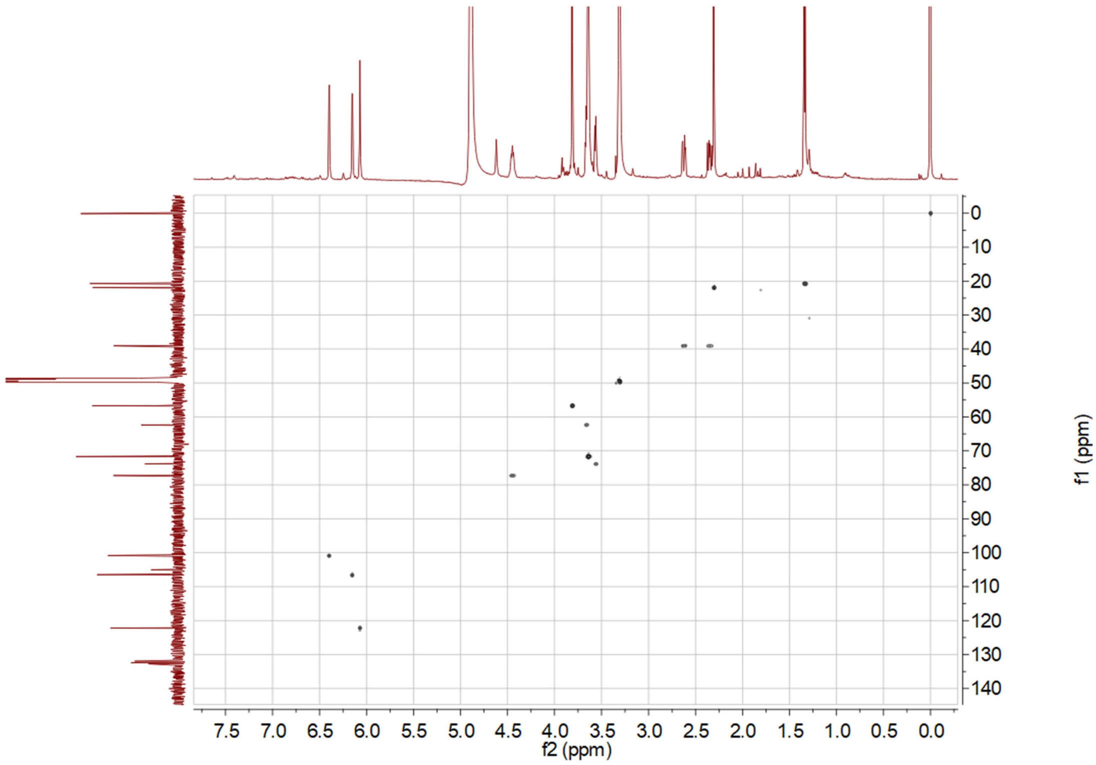

Figure S4: HSQC spectrum of compound 1

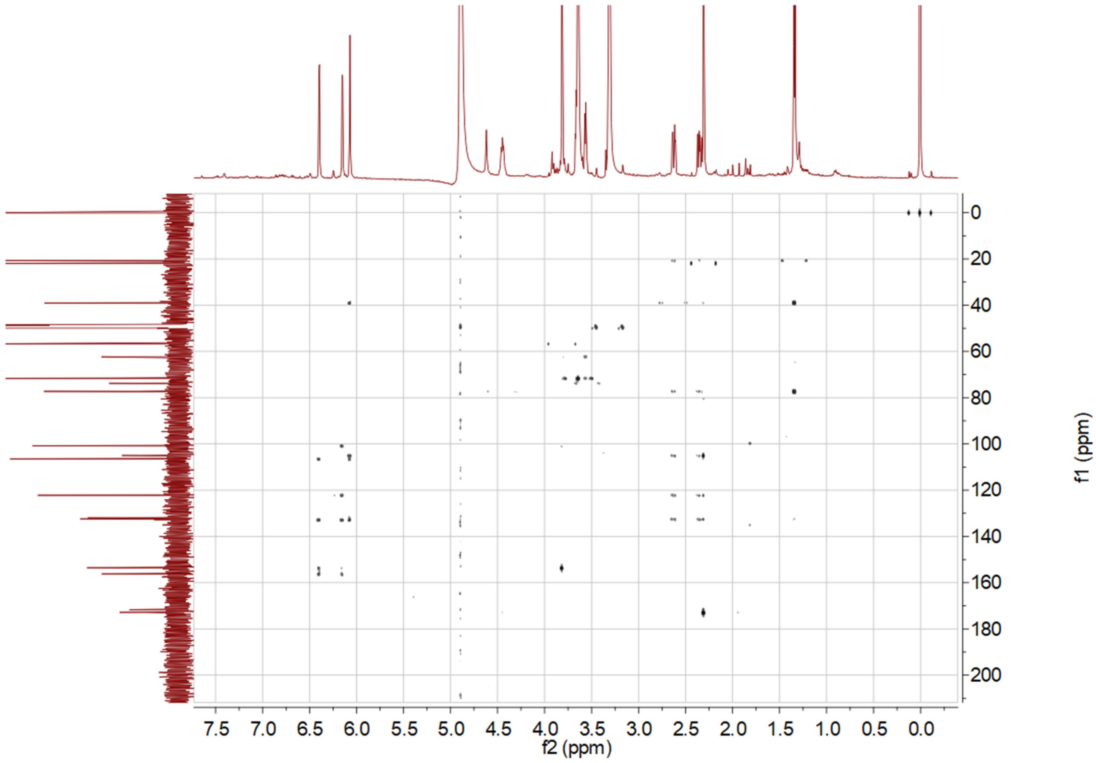

Figure S5: HMBC spectrum of compound 1

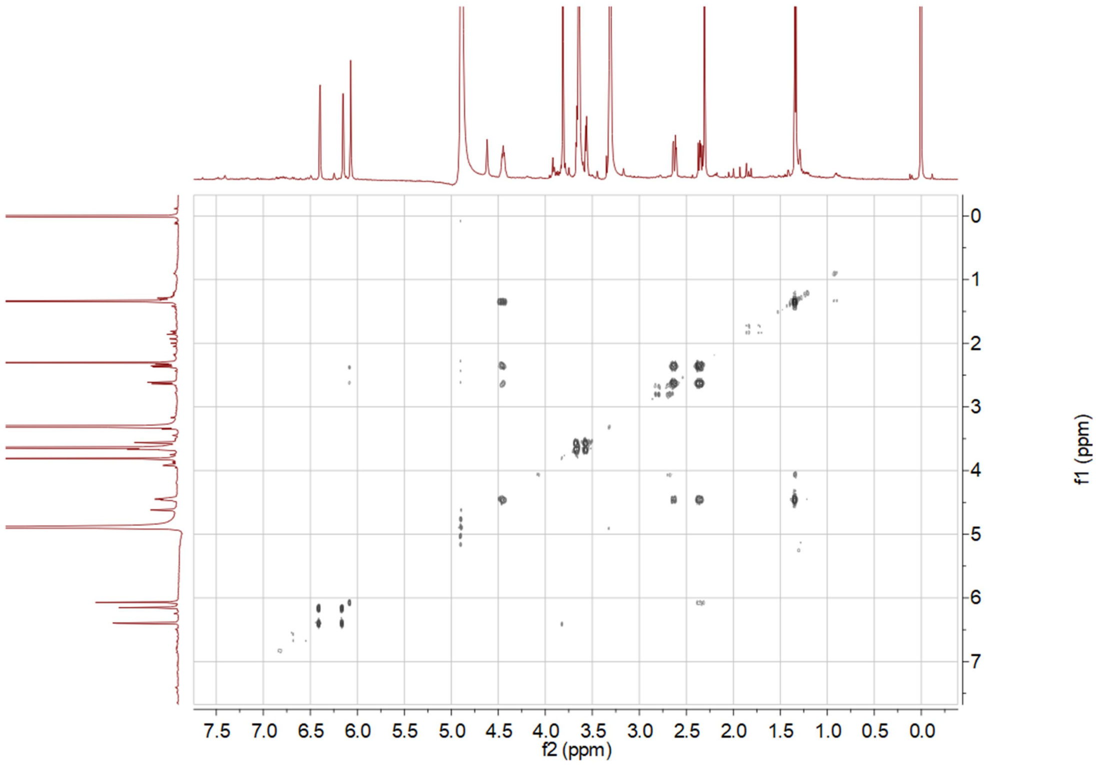

Figure S6: ^{1}H-^{1}H COSY spectrum of compound 1

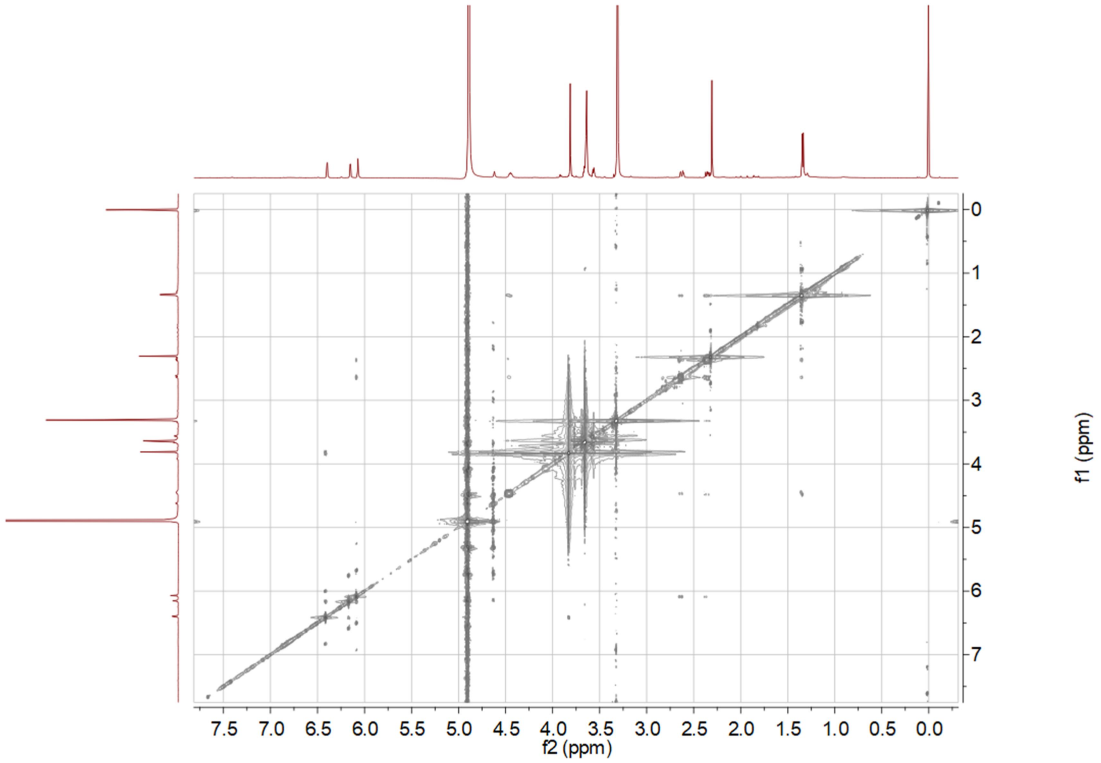

Figure S7: ROESY spectrum of compound 1

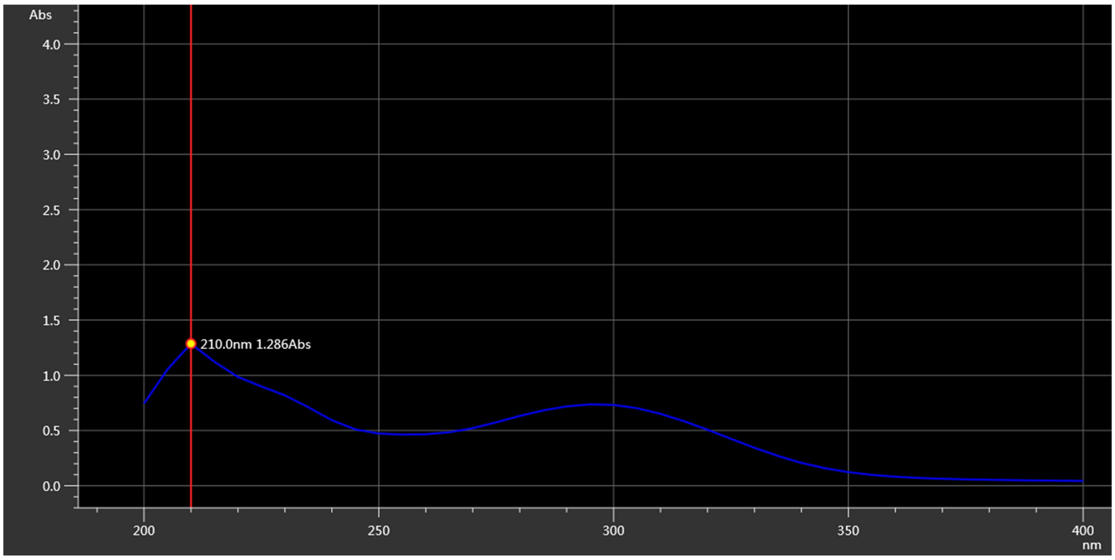

Figure S8: UV spectrum of compound 1

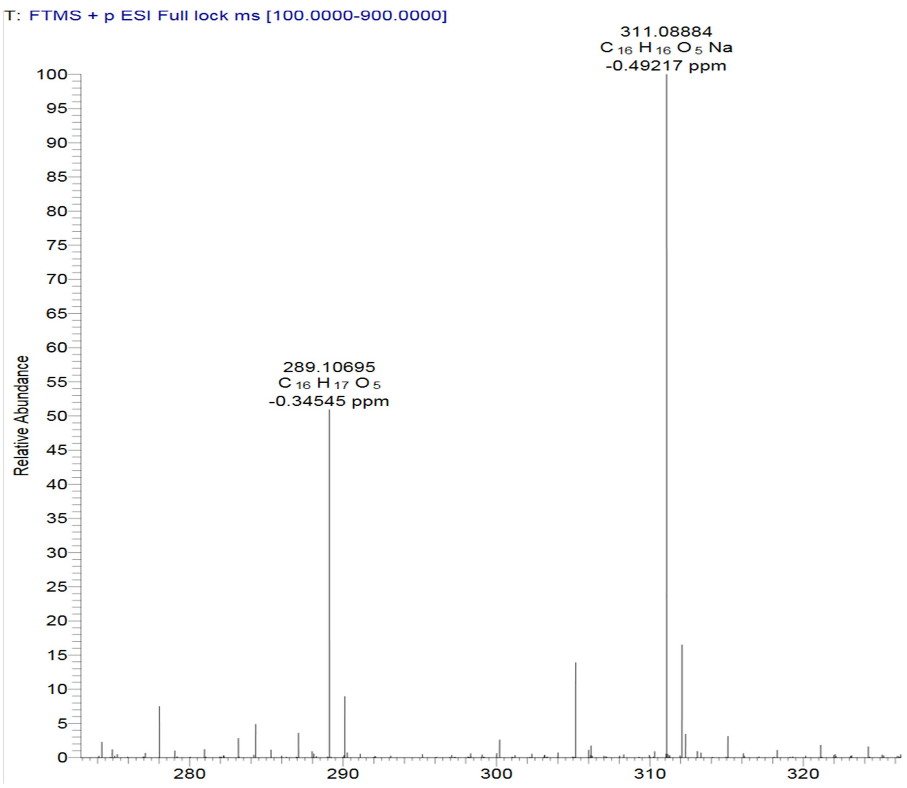

Figure S9: HR-ESI-MS of compound 1

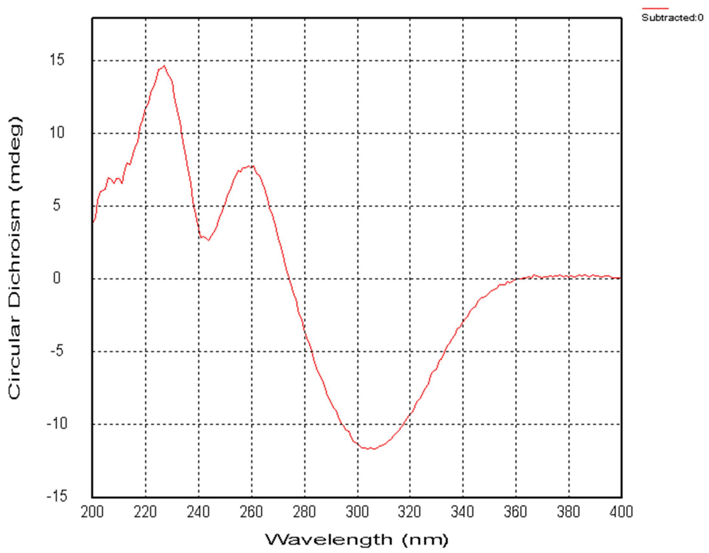

Figure S10: CD spectrum of compound 1

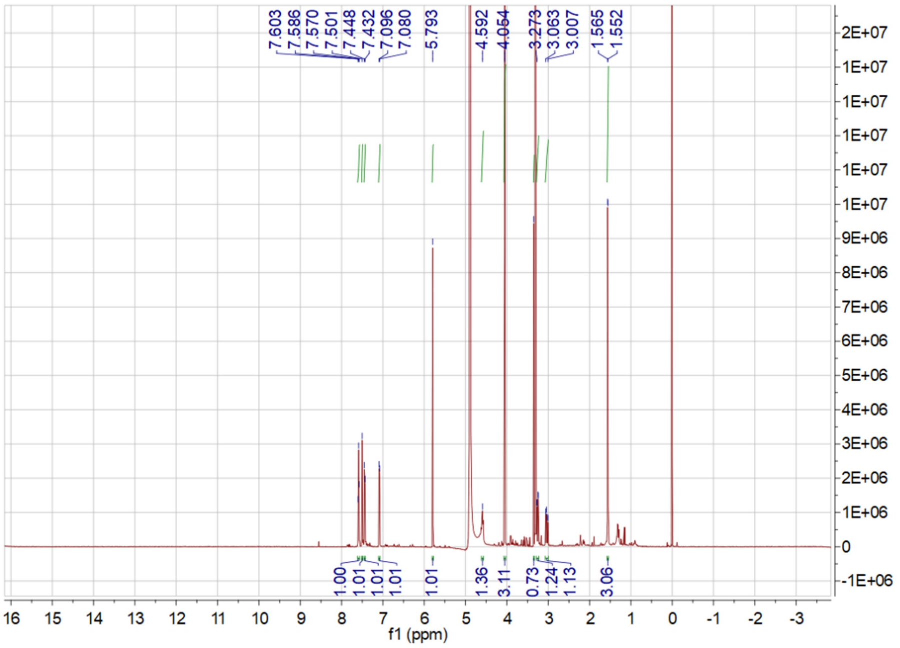

Figure S11: ^{1}H-NMR (500 MHz, CD_3OD) spectrum of compound 2

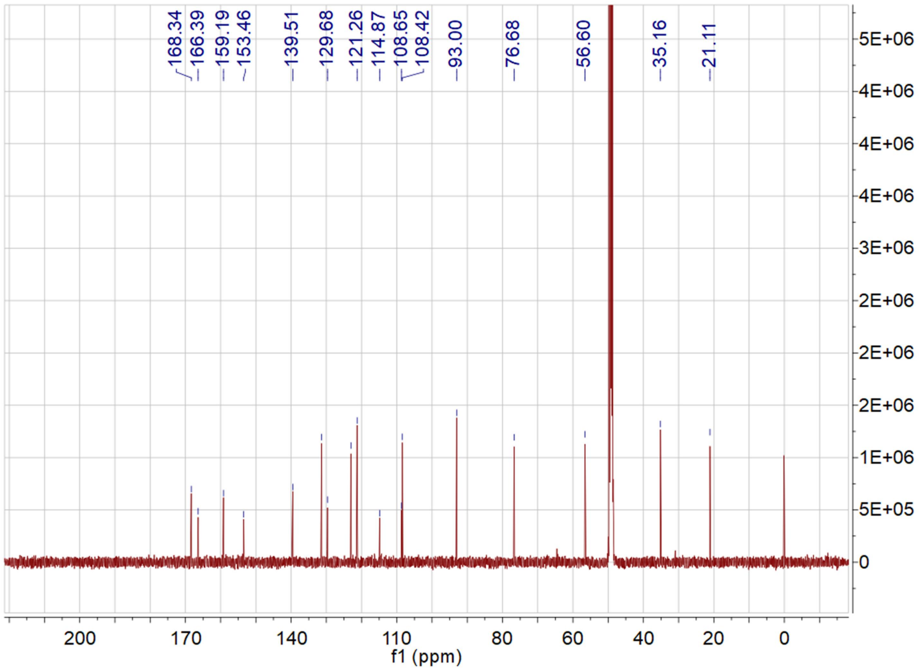

Figure S12: ^{13}C-NMR (125 MHz, CD_3OD) spectrum of compound 2

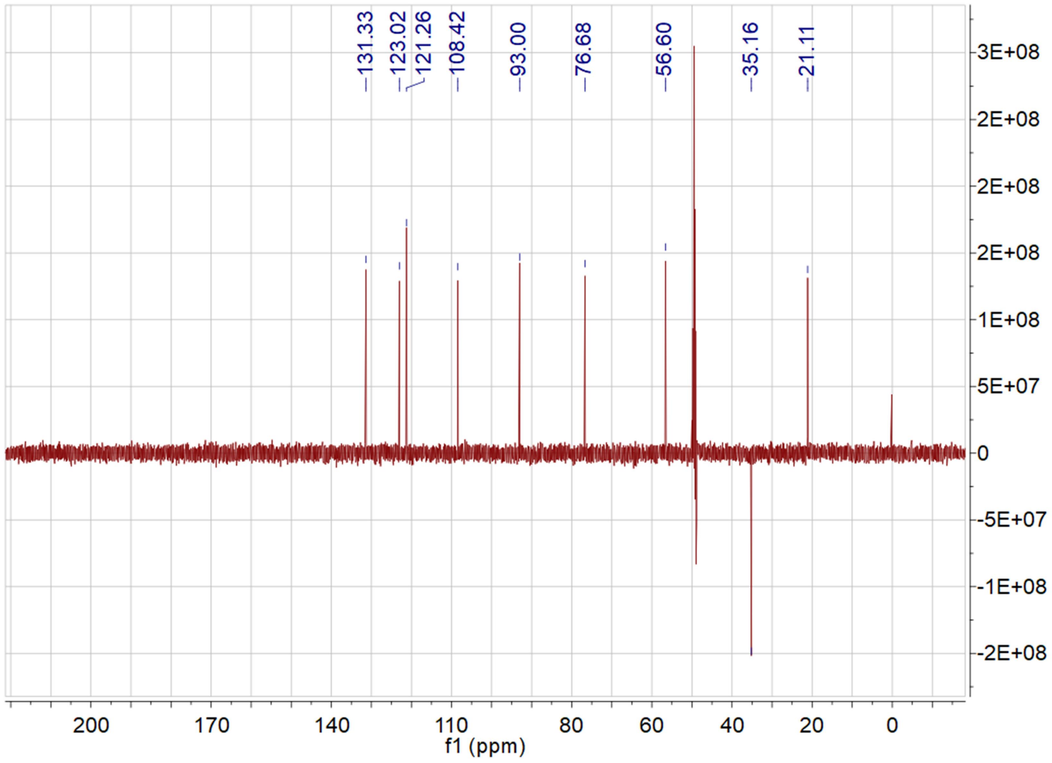

Figure S13: 13C-NMR-DEPT (θ=135°) spectrum of compound 2

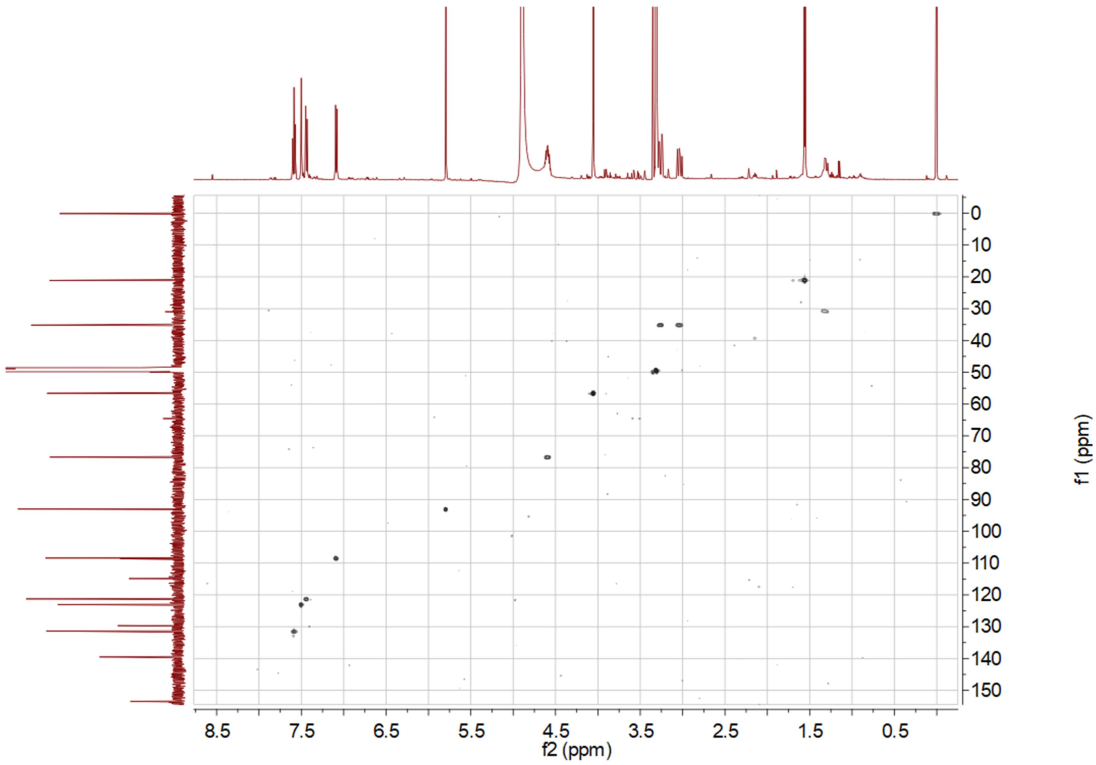

Figure S14: HSQC spectrum of compound 2

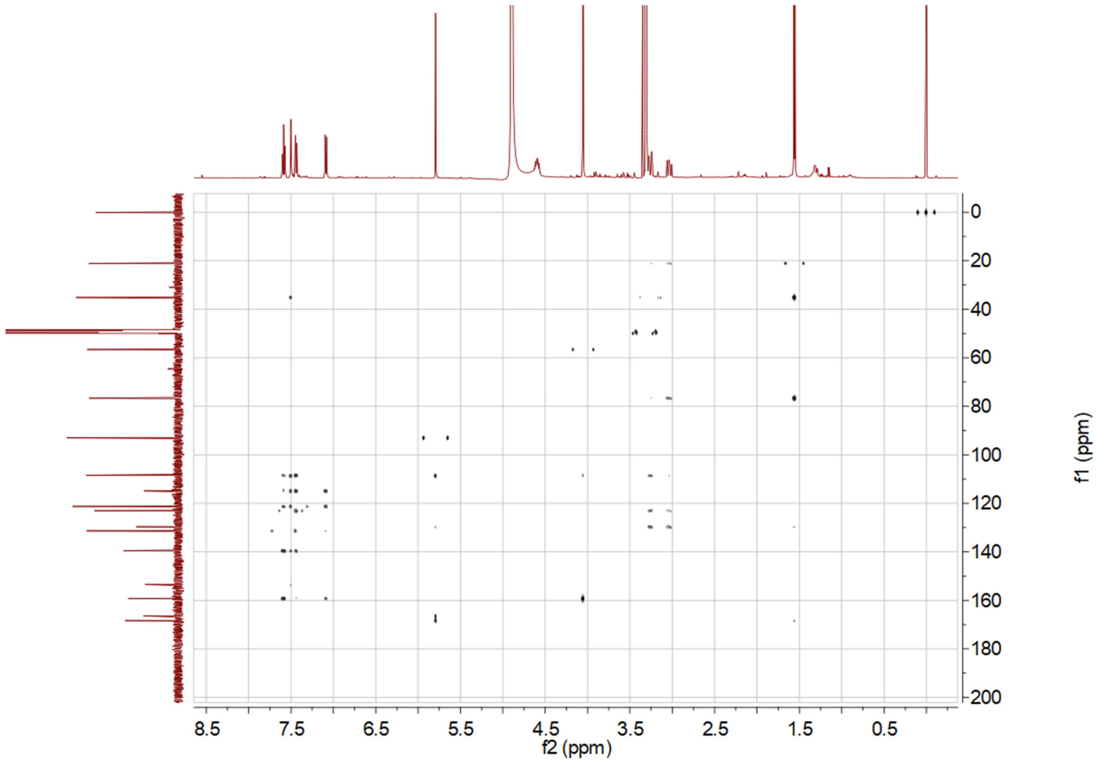

Figure S15: HMBC spectrum of compound 2

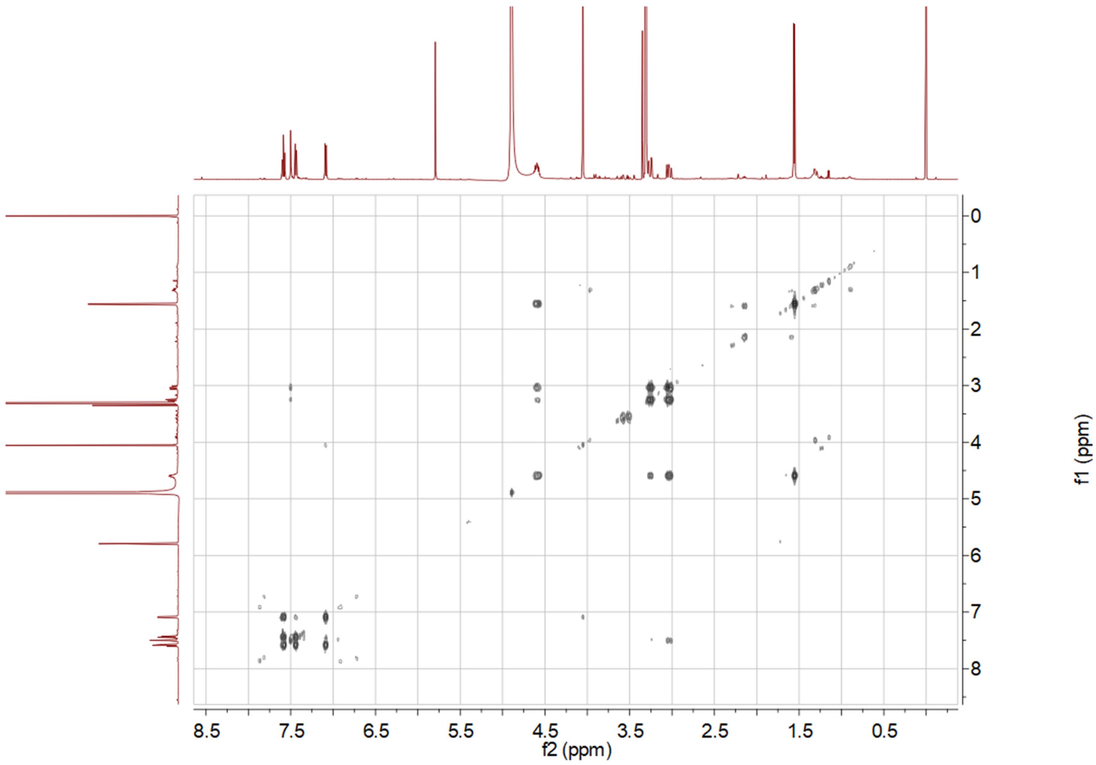

Figure S16: ^{1}H-^{1}H COSY spectrum of compound 2

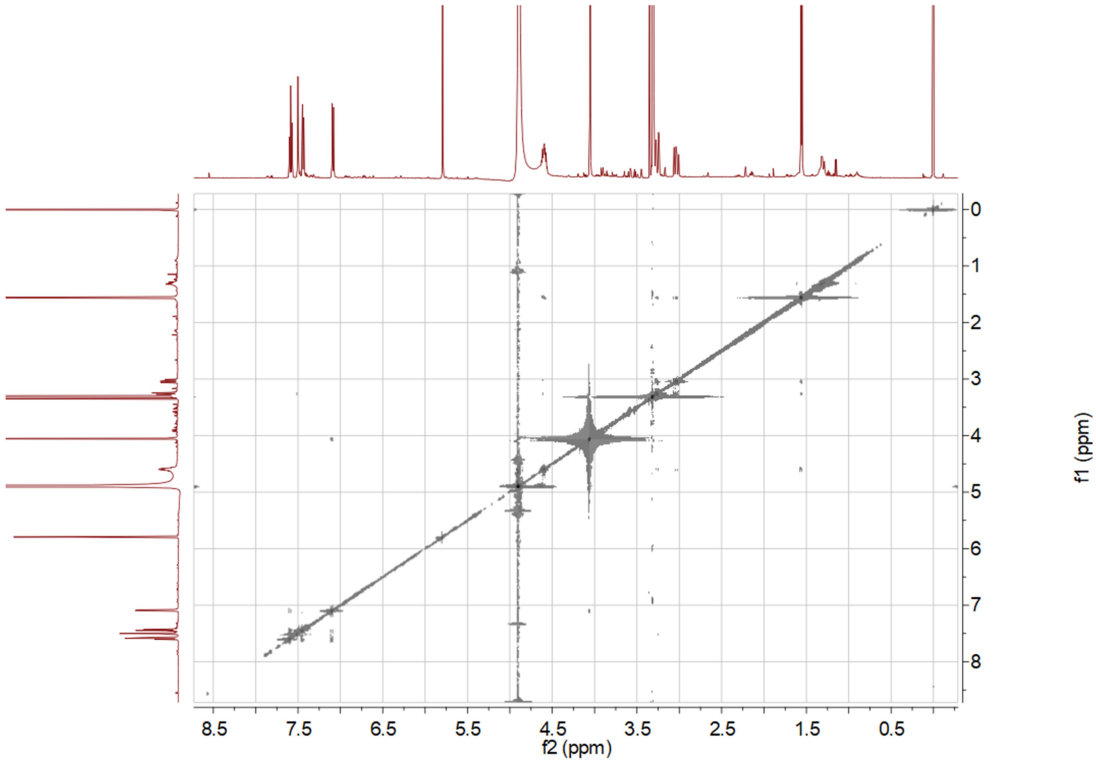

Figure S17: ROESY spectrum of compound 2

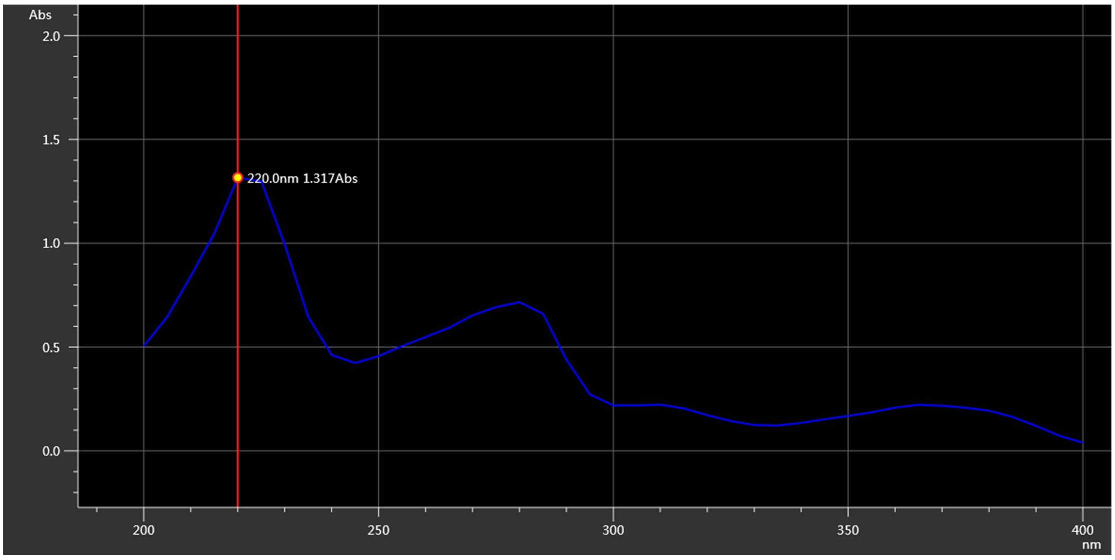

Figure S18: UV spectrum of compound 2

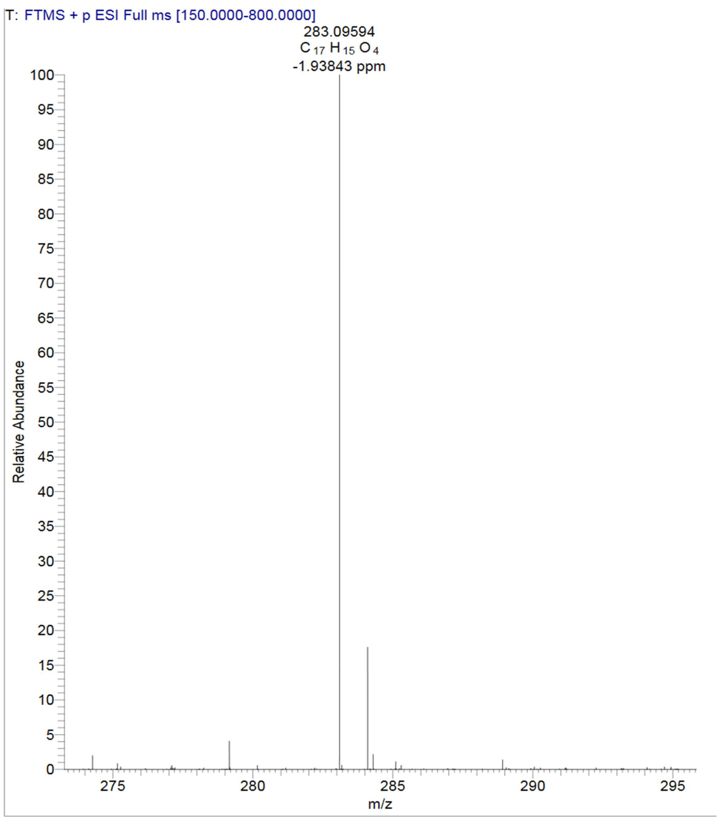

Figure S19: HR-ESI-MS of compound 2

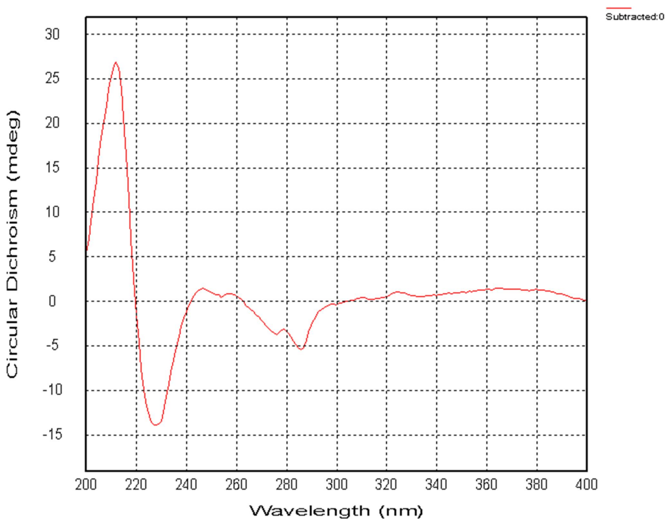

Figure S20: CD spectrum of compound 2

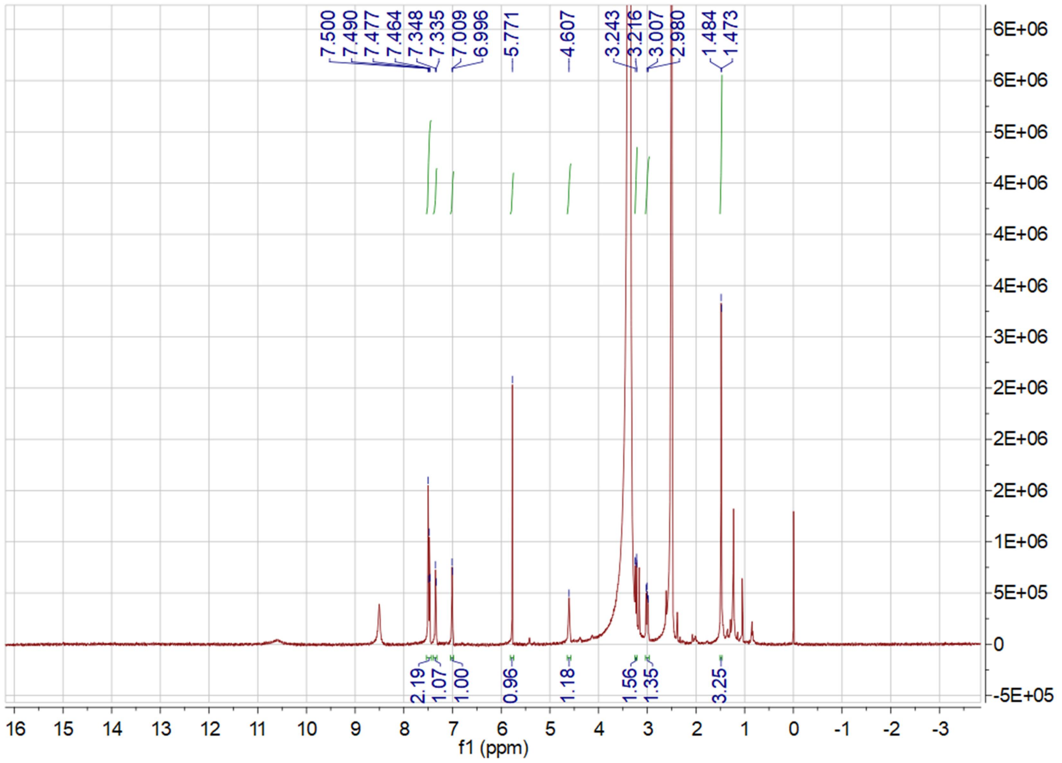

Figure S21: ^{1}H-NMR (600 MHz, DMSO) spectrum of compound 3

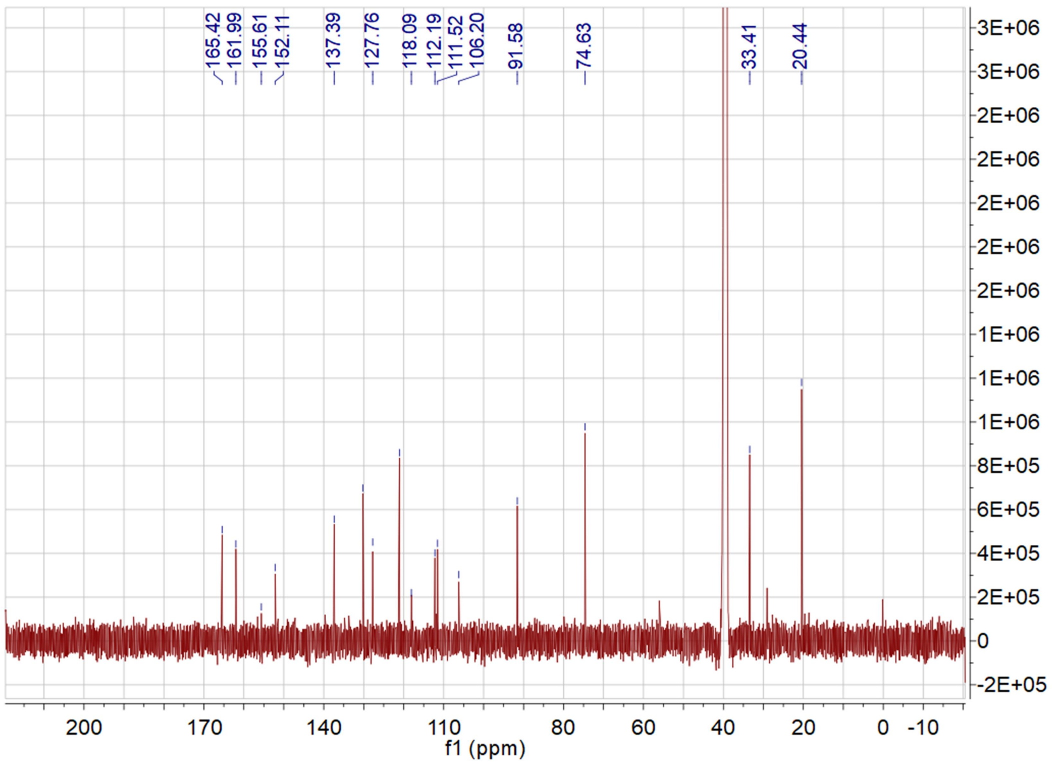

Figure S22: The ^{13}C-NMR (150 MHz, DMSO) spectrum of compound 3

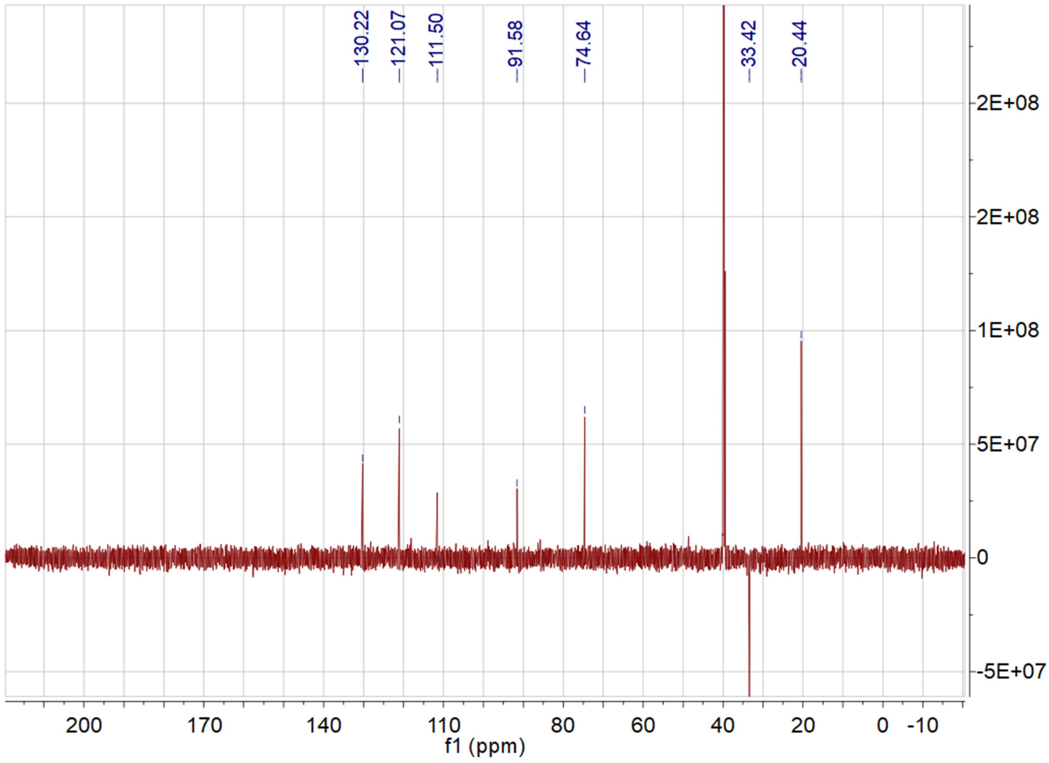

Figure S23: ^{13}C-NMR-DEPT (θ=135°) spectrum of compound 3

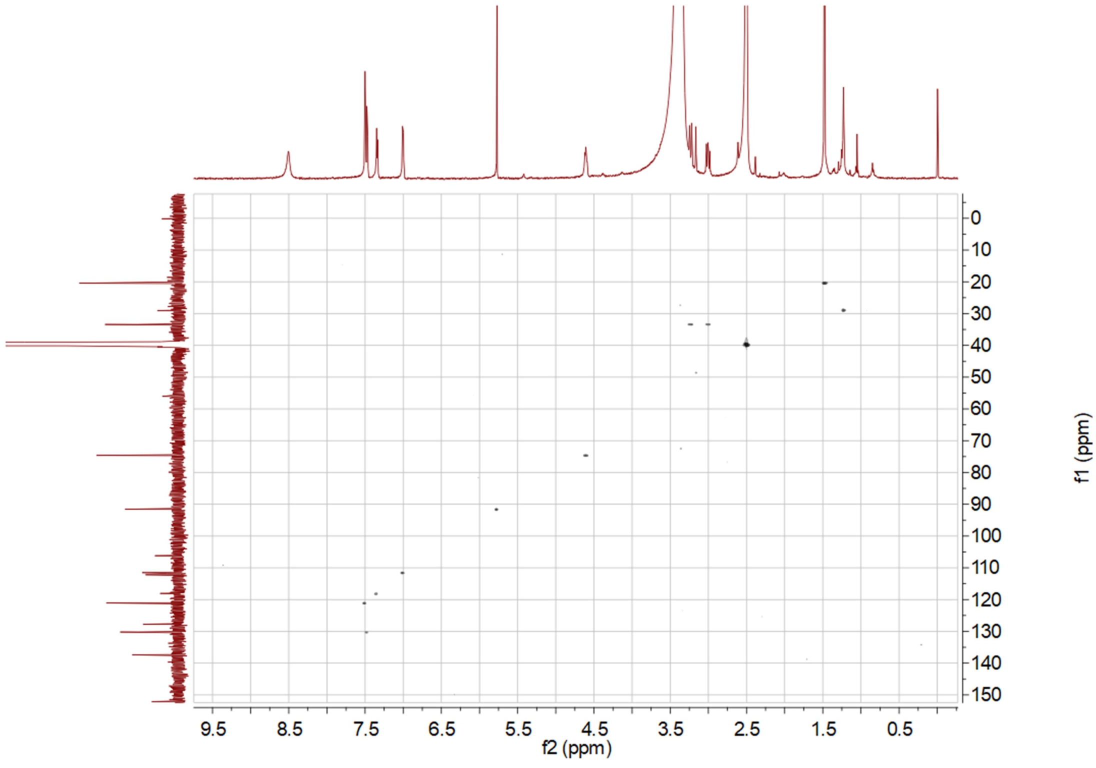

Figure S24: HSQC spectrum of compound 3

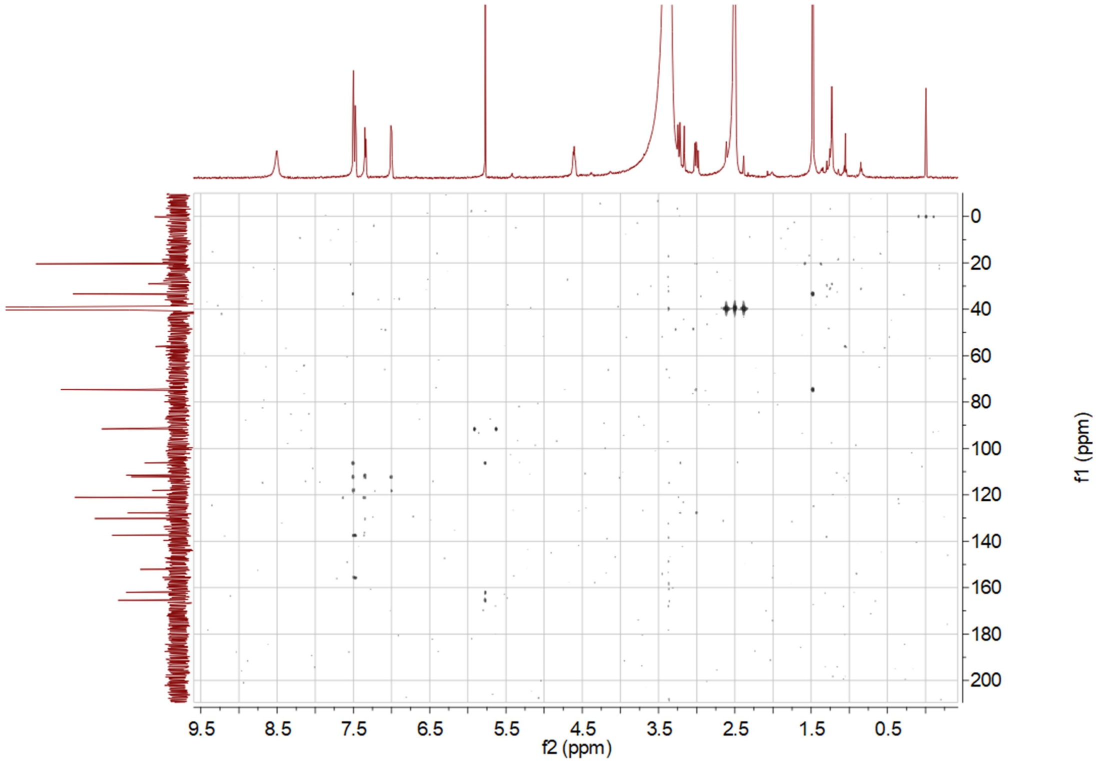

Figure S25: HMBC spectrum of compound 3

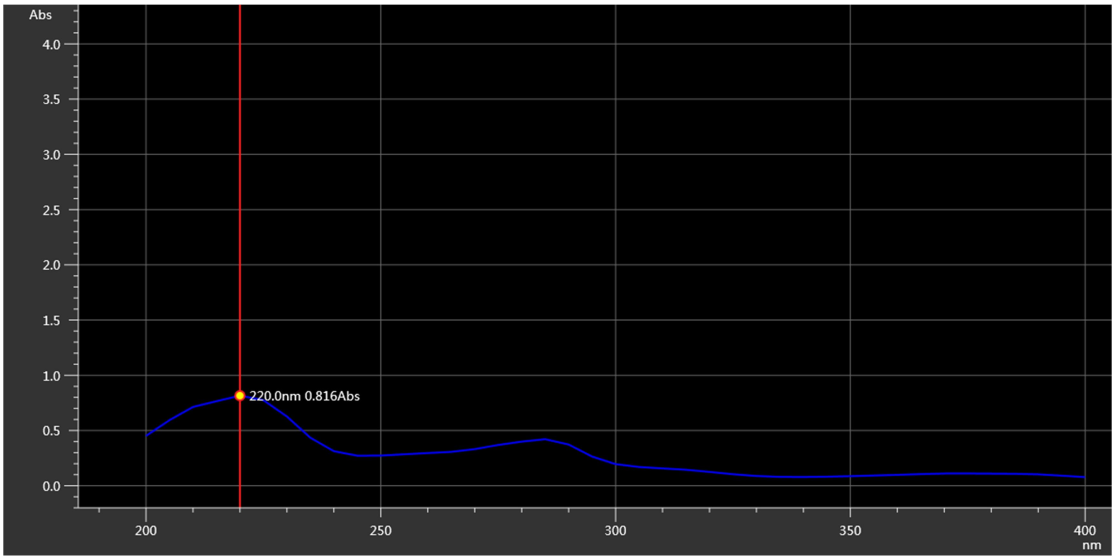

Figure S26: UV spectrum of compound 3

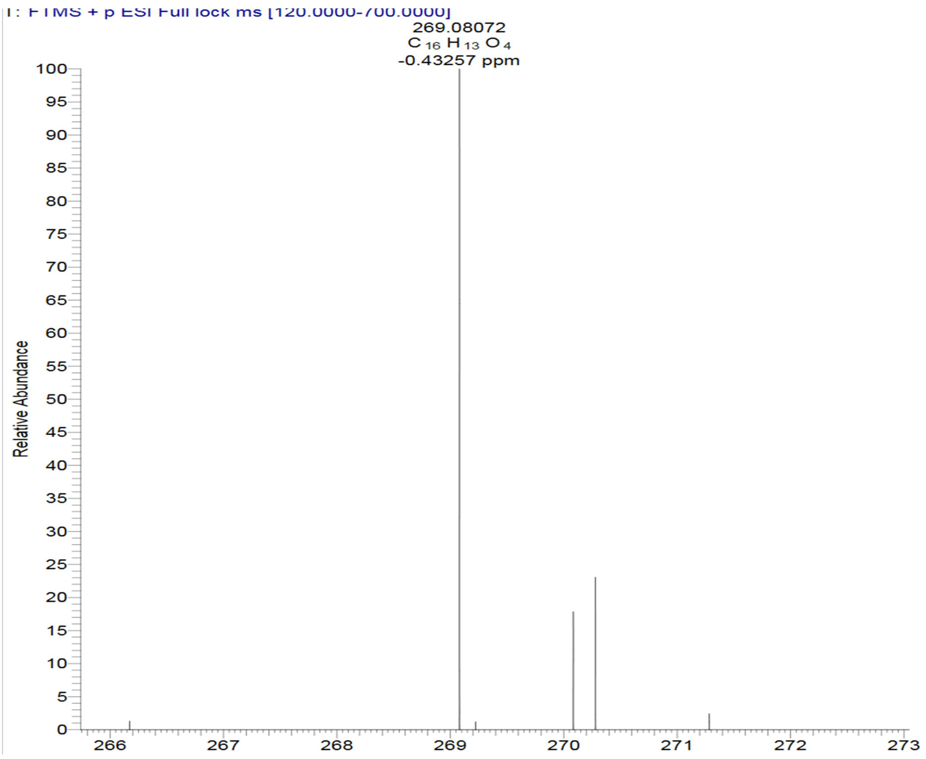

Figure S27: HR-ESI-MS of compound 3

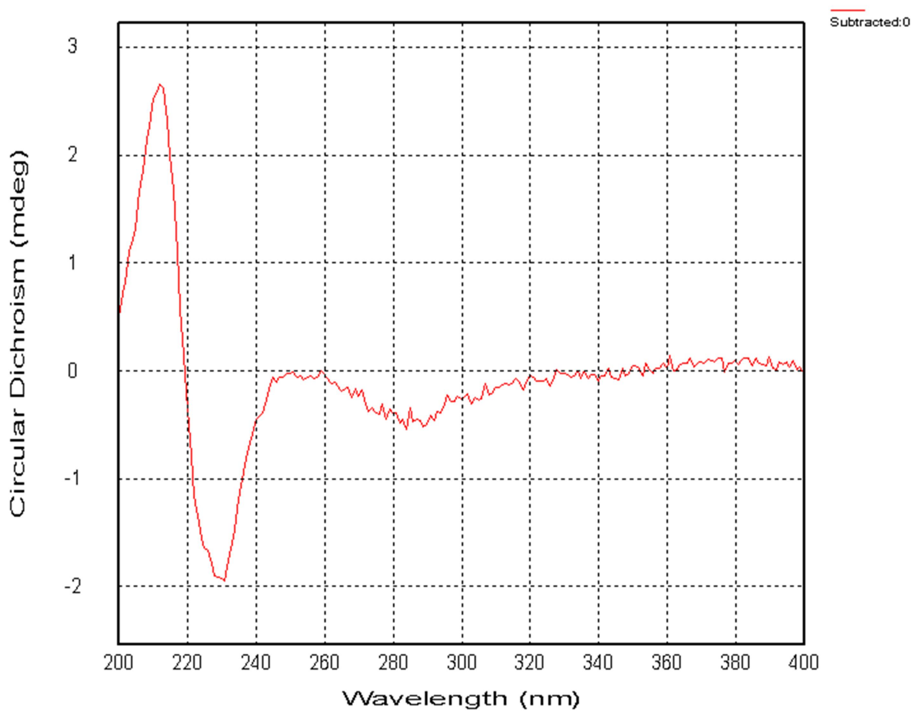

Figure S28: CD spectrum of compound 3

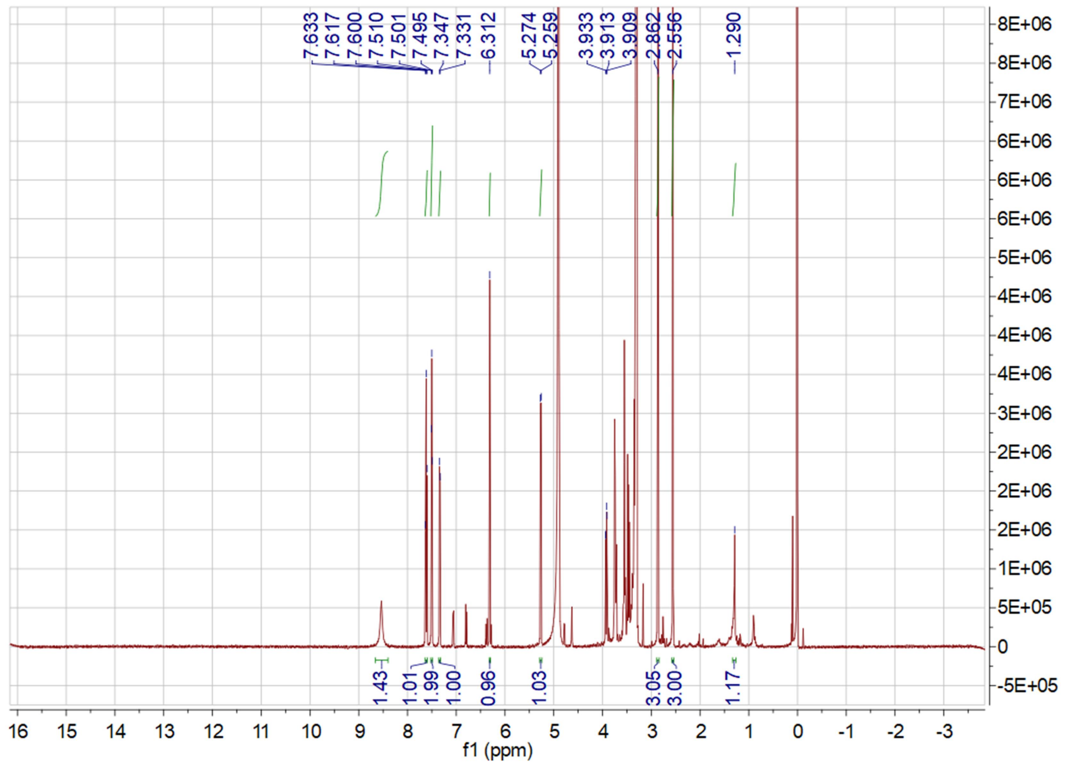

Figure S29: ^{1}H-NMR (500 MHz, CD_3OD) spectrum of compound 4

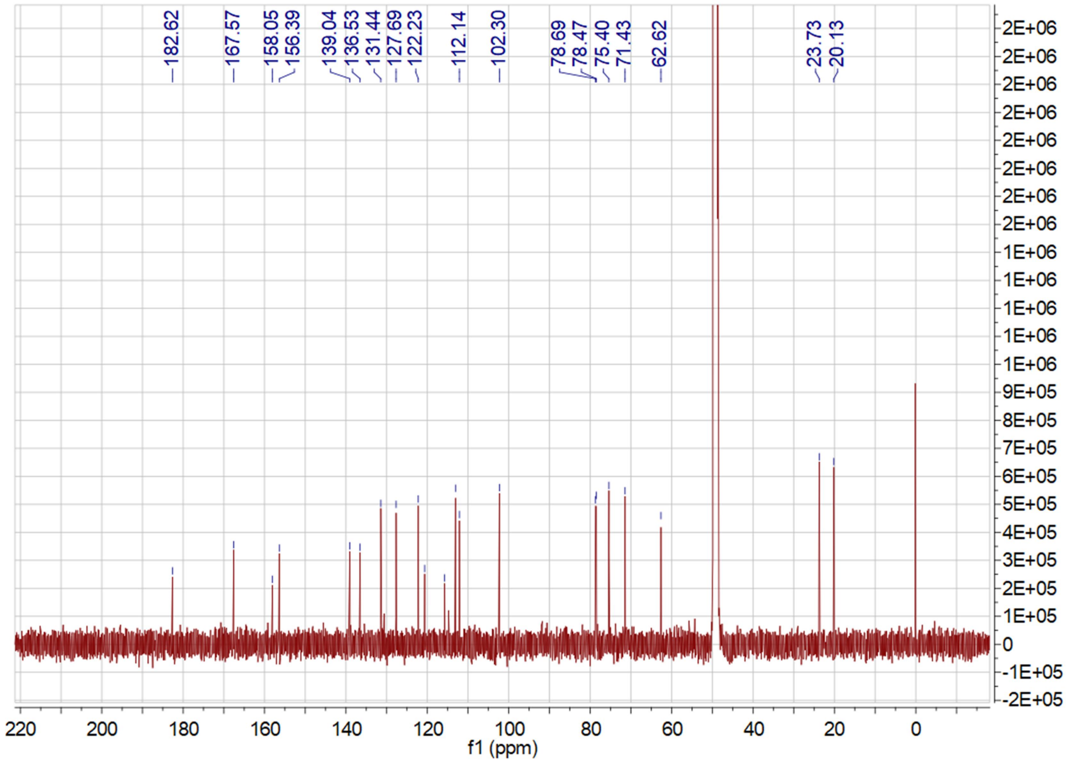

Figure S30: The ^{13}C-NMR (125 MHz, CD_3OD) spectrum of compound 4

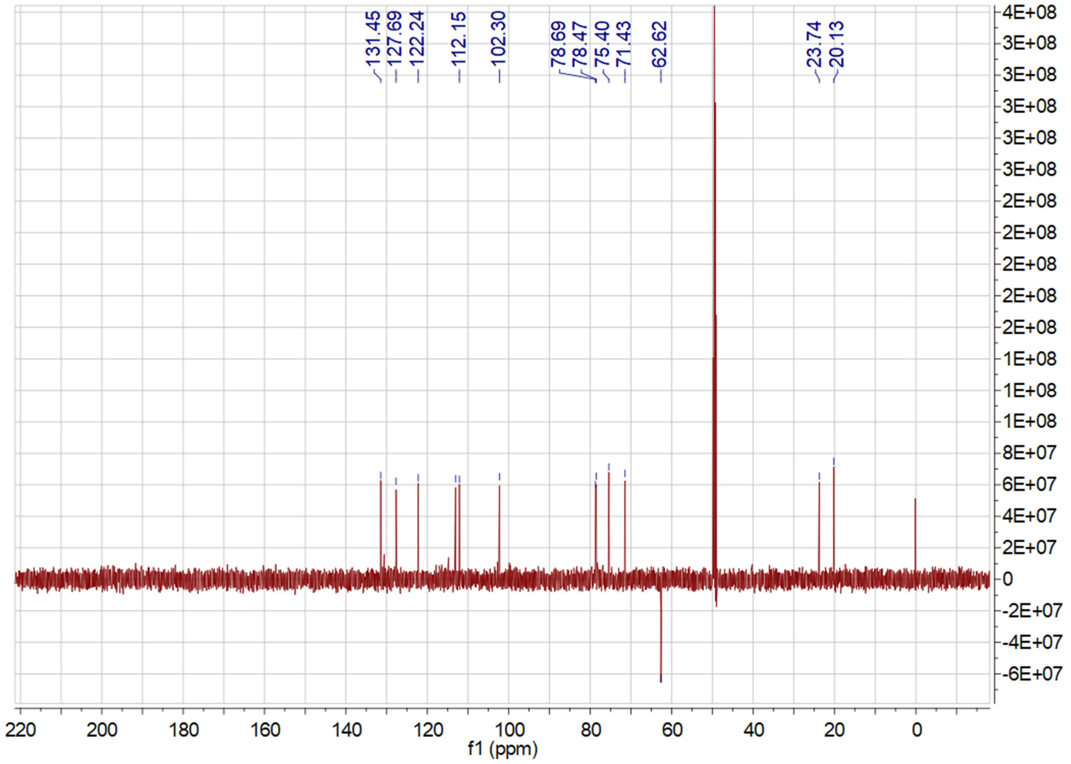

Figure S31: ^{13}C-NMR-DEPT (θ=135°) spectrum of compound 4

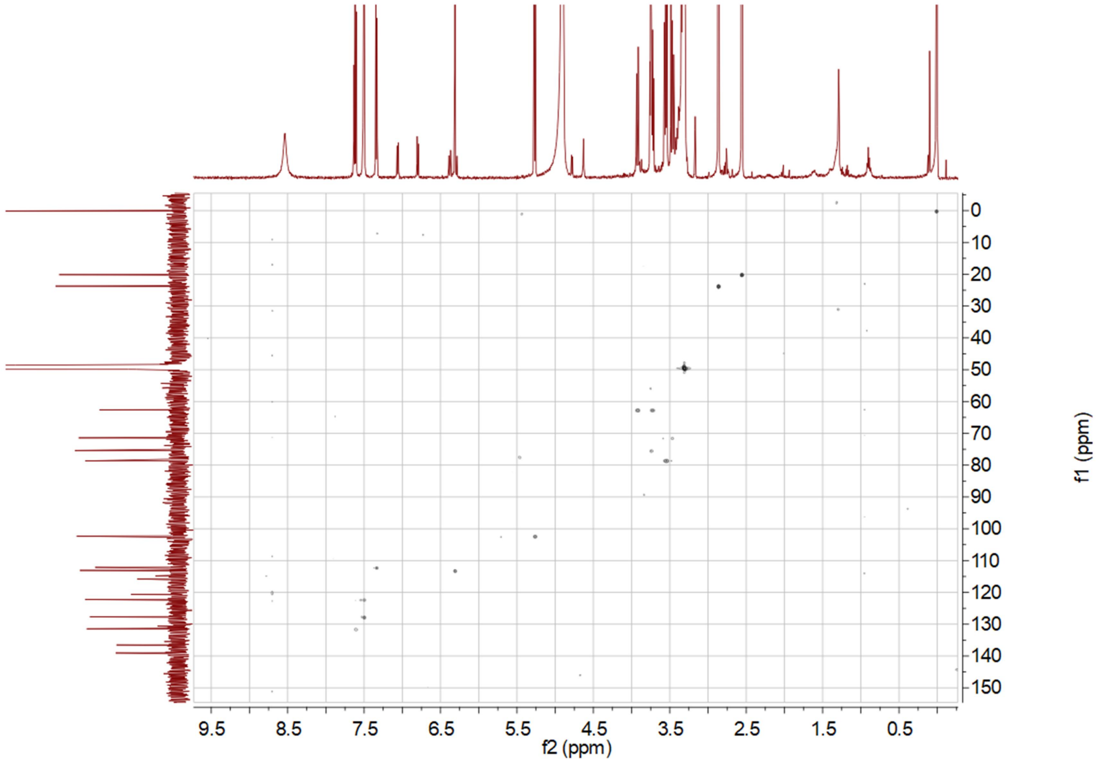

Figure S32: HSQC spectrum of compound 4

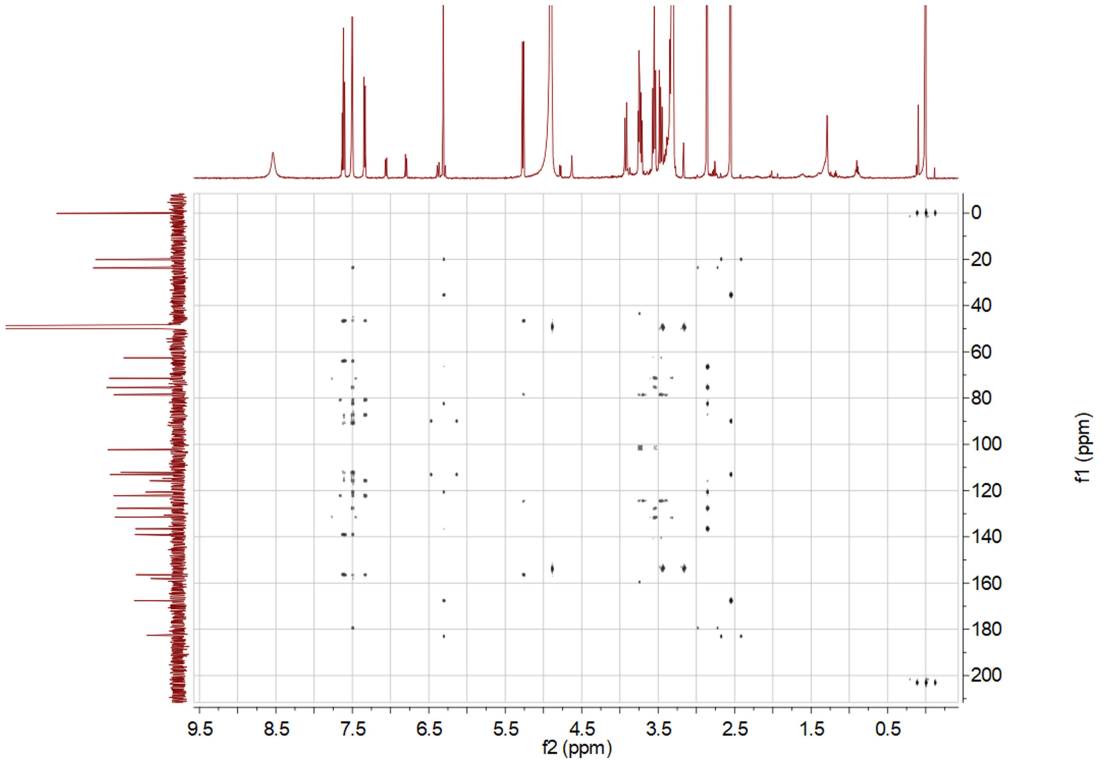

Figure S33: HMBC spectrum of compound 4

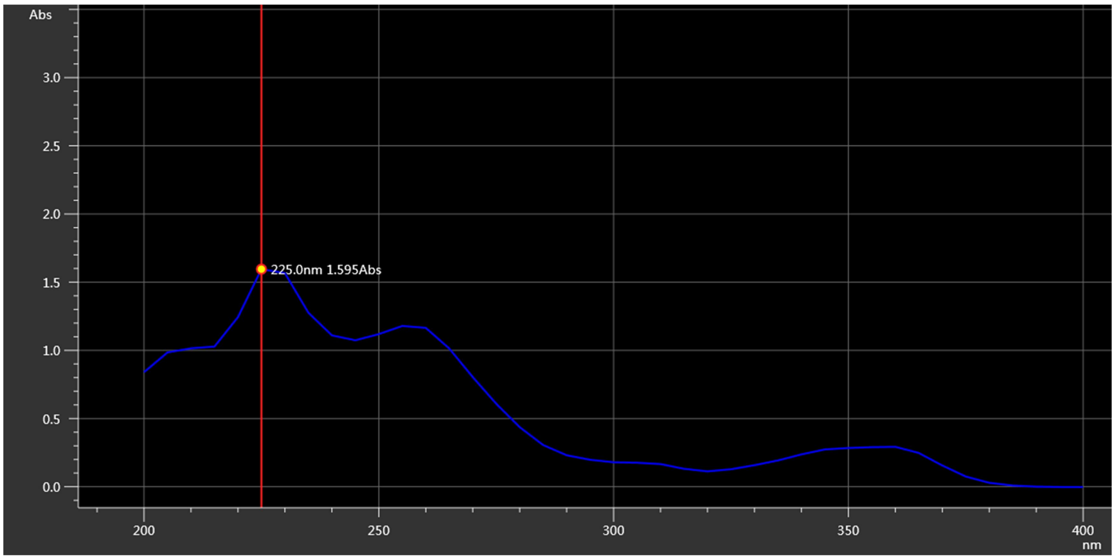

Figure S34: UV spectrum of compound 4

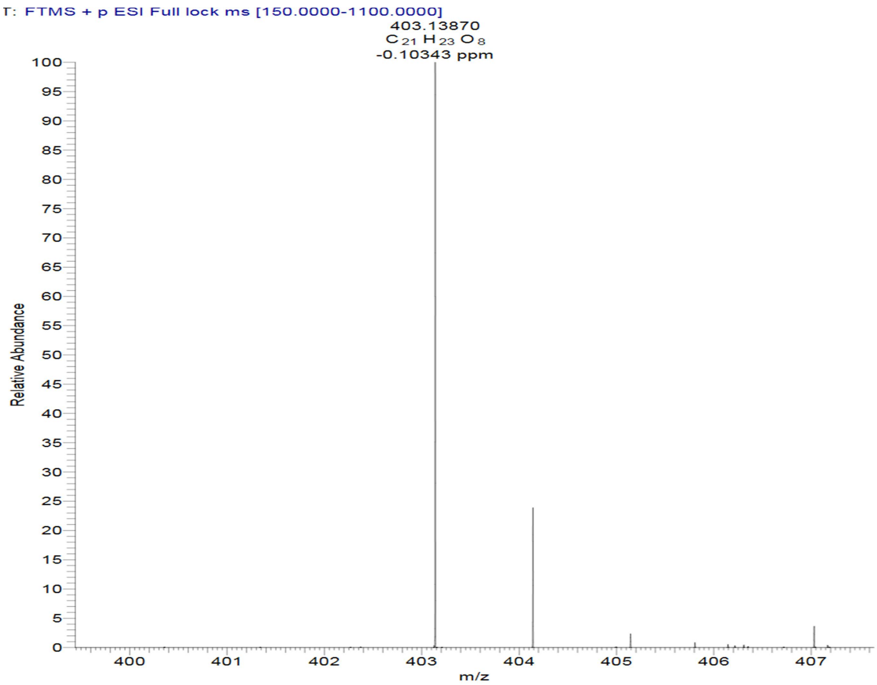

Figure S35: HR-ESI-MS of compound 4

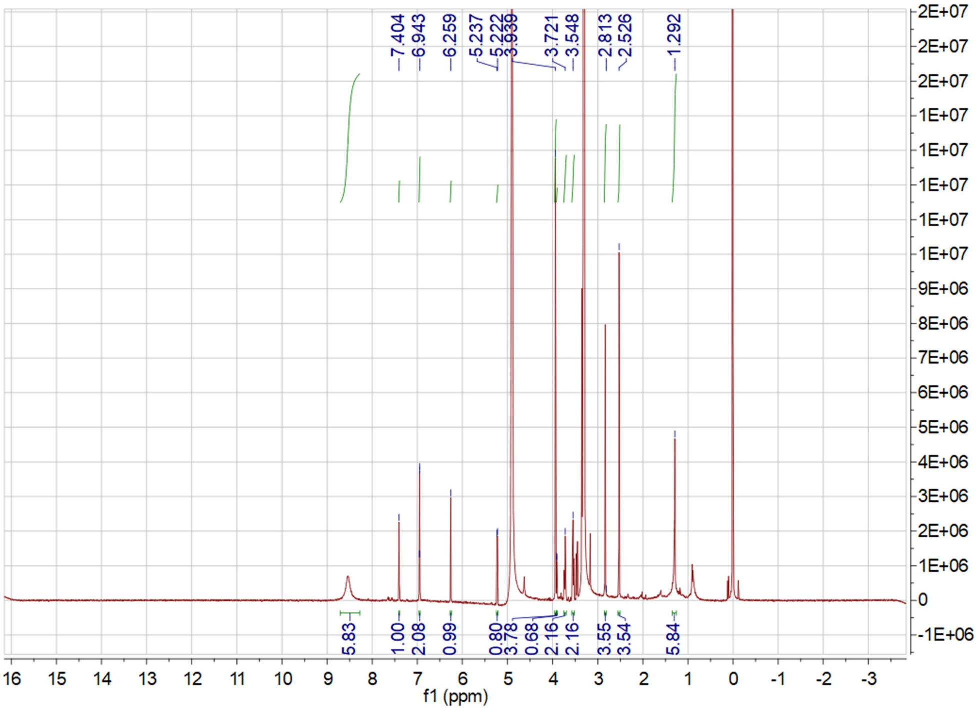

Figure S36: ^{1}H-NMR (500 MHz, CD_3OD) spectrum of compound 5

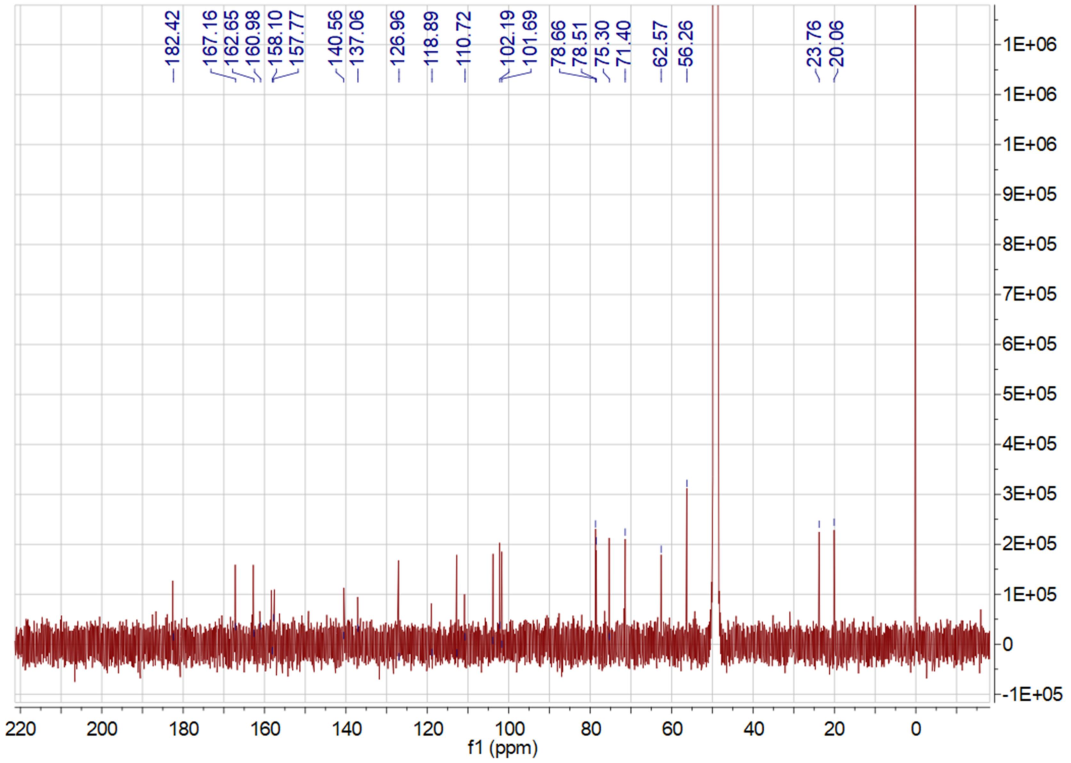

Figure S37: ^{13}C-NMR (125 MHz, CD_3OD) spectrum of compound 5

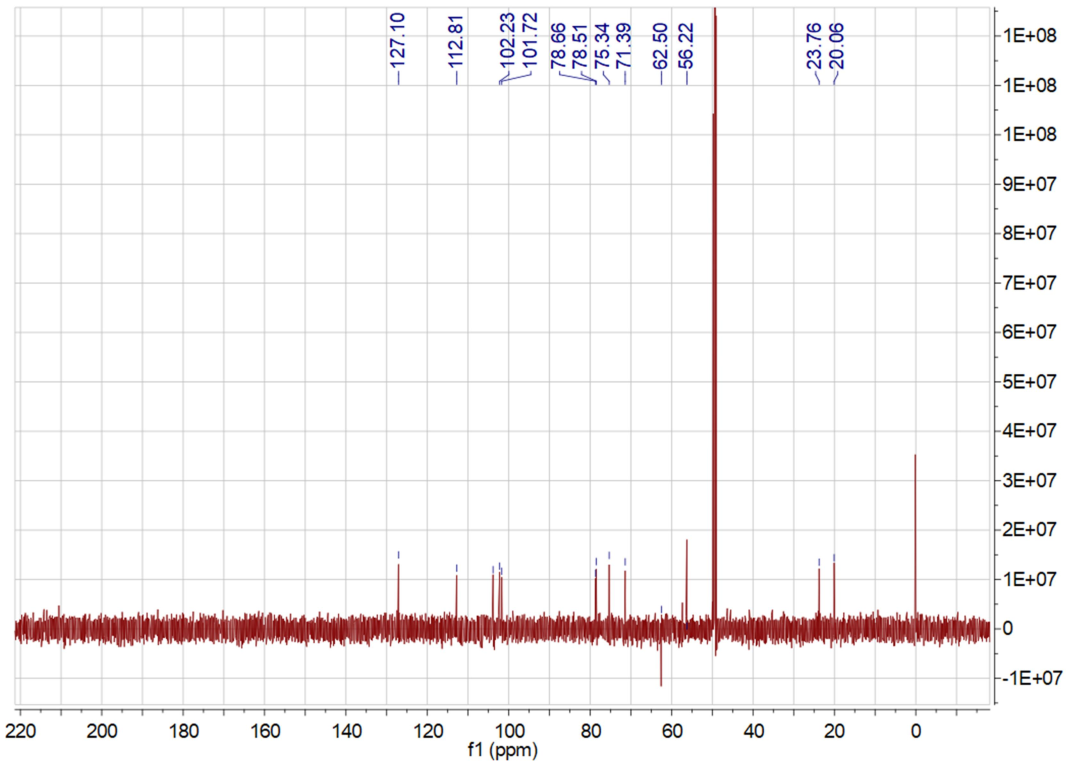

Figure S38: ^{13}C-NMR-DEPT (θ=135°) spectrum of compound 5

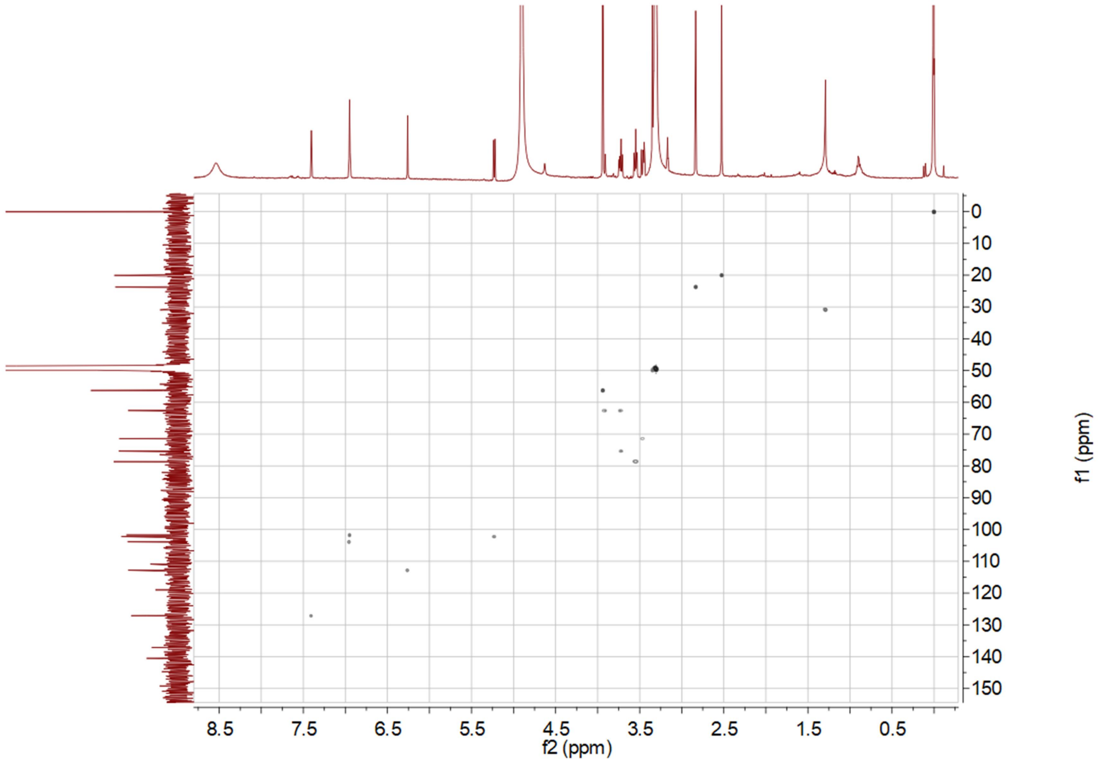

Figure S39: HSQC spectrum of compound 5

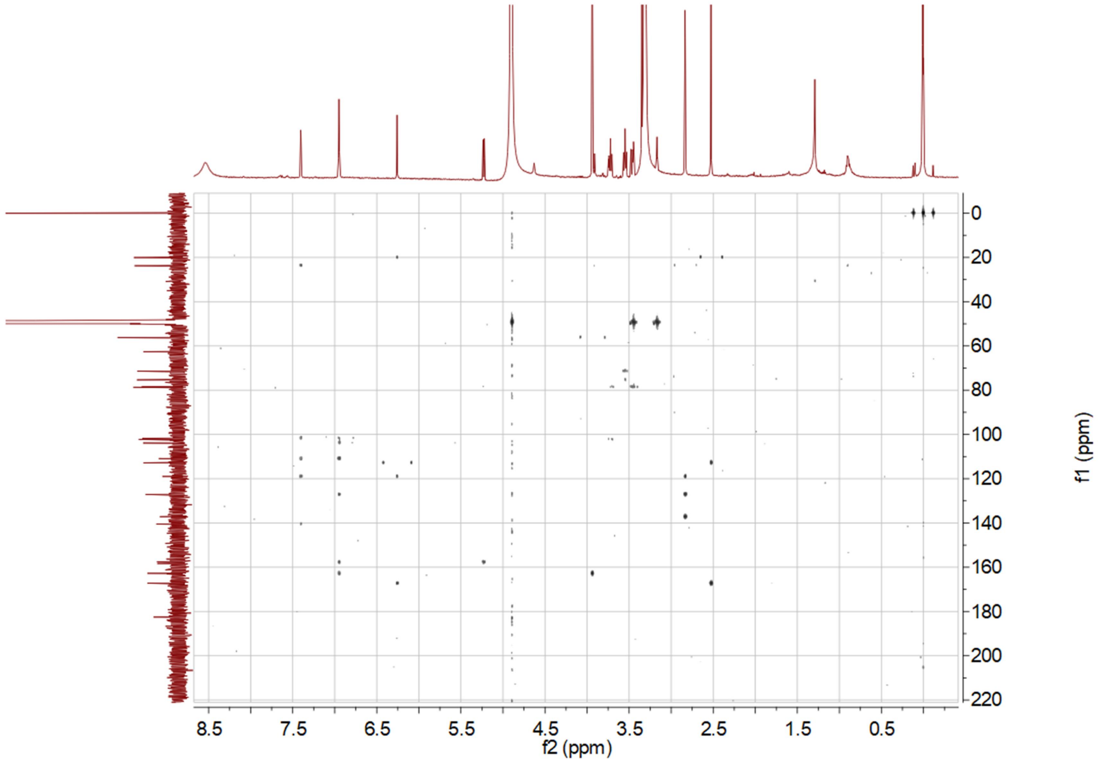

Figure S40: HMBC spectrum of compound 5

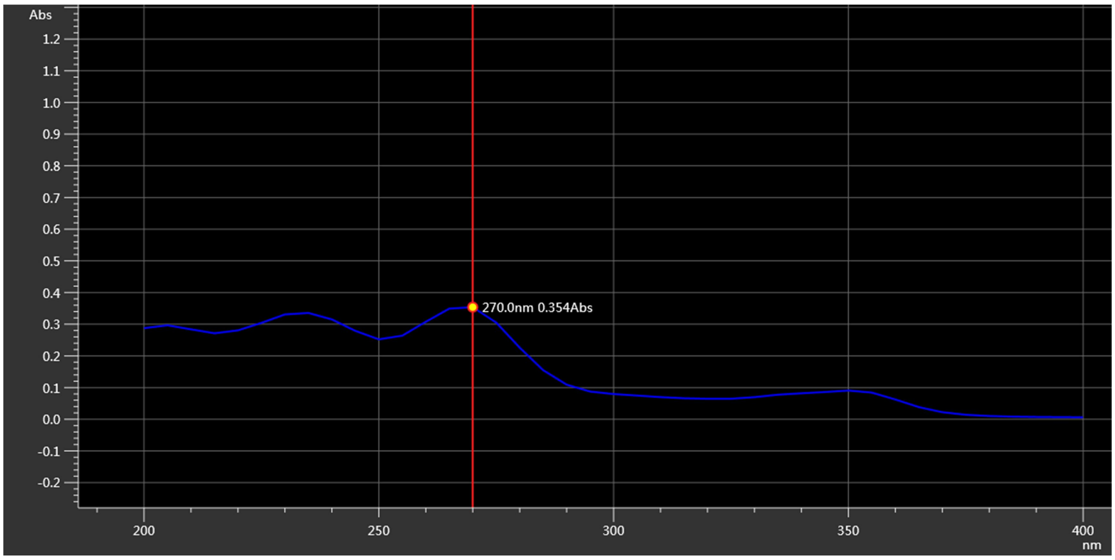

Figure S41: UV spectrum of compound 5

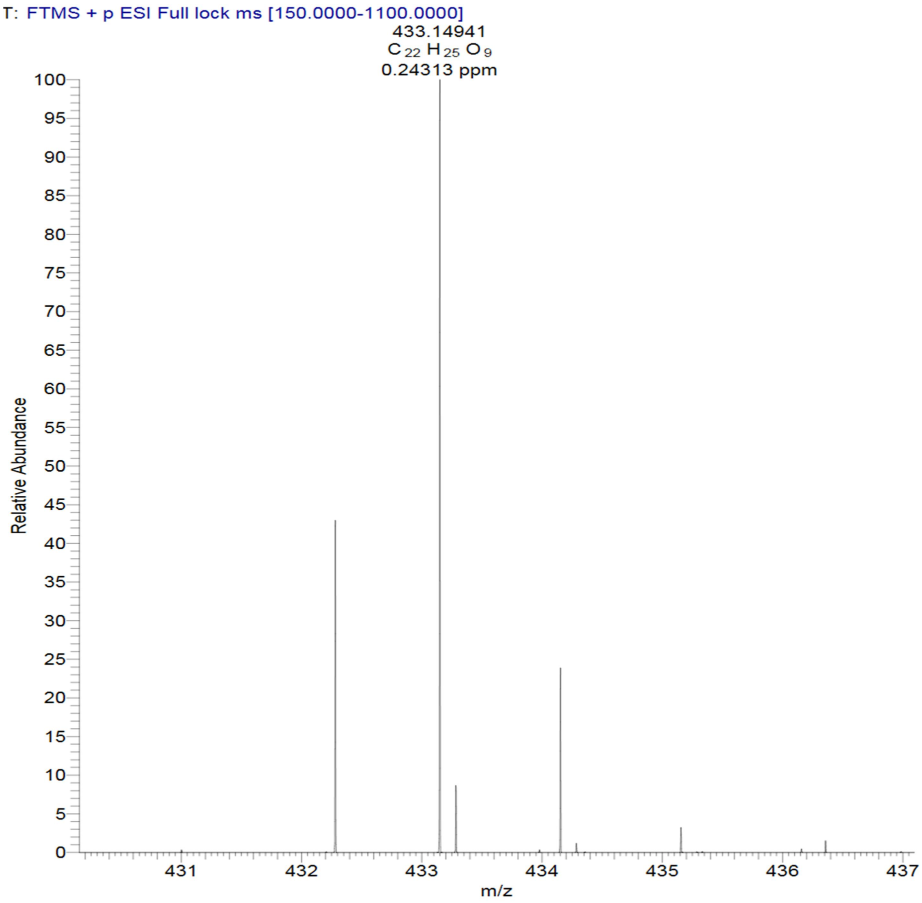

Figure S42: HR-ESI-MS of compound 5

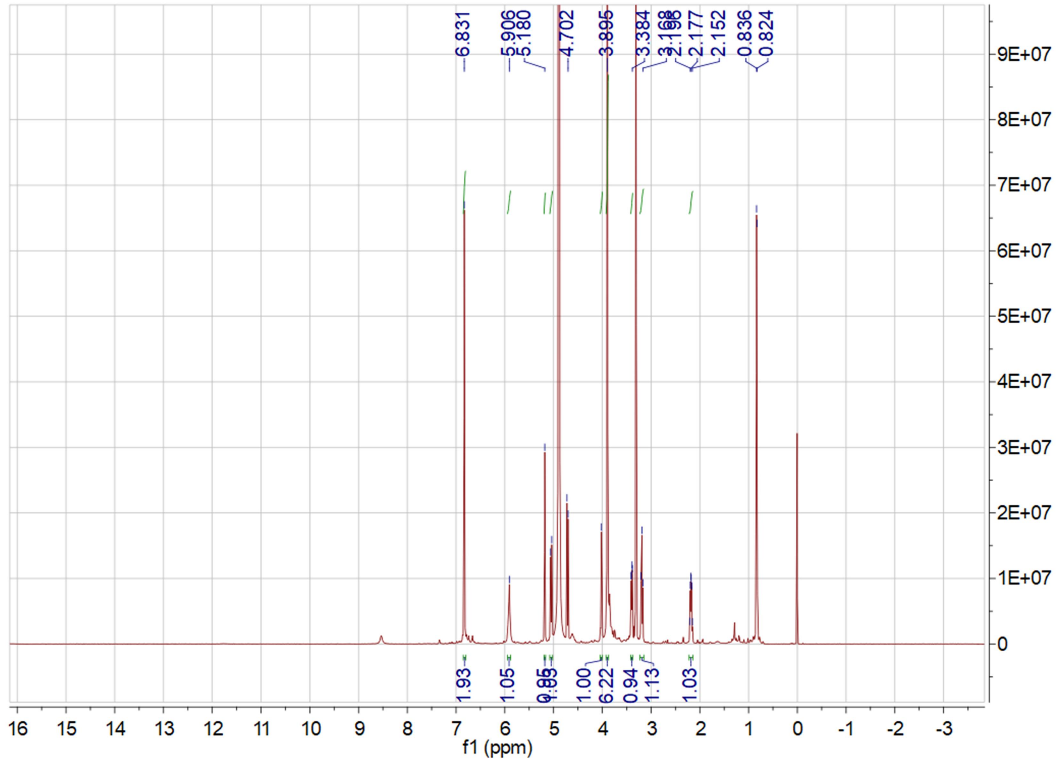

Figure S43: ^{1}H-NMR (500 MHz, CD_3OD) spectrum of compound 6

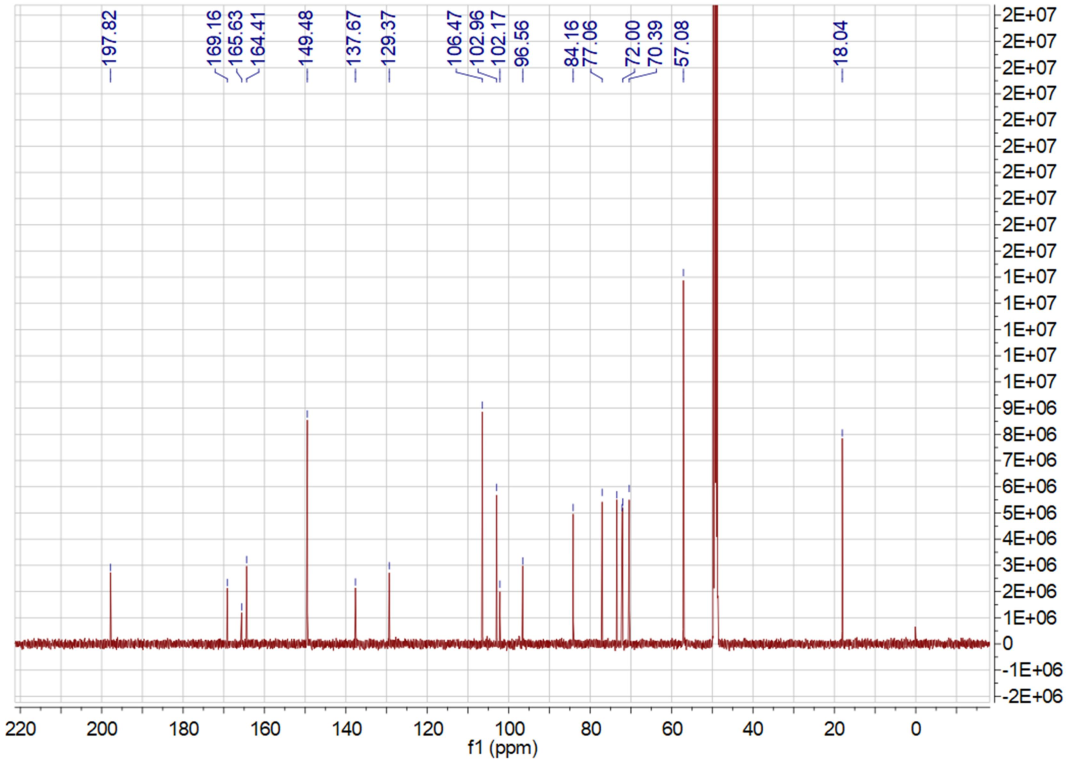

Figure S44: ^{13}C-NMR (125 MHz, CD_3OD) spectrum of compound 6

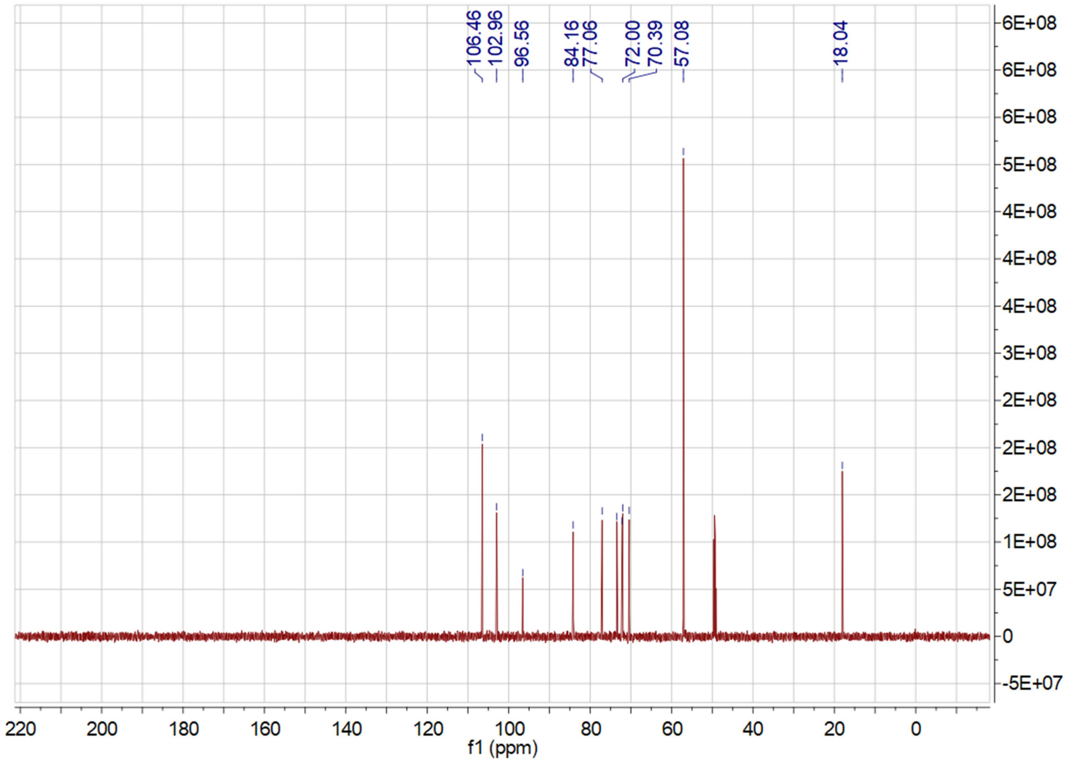

Figure S45: ^{13}C-NMR-DEPT (θ=135°) spectrum of compound 6

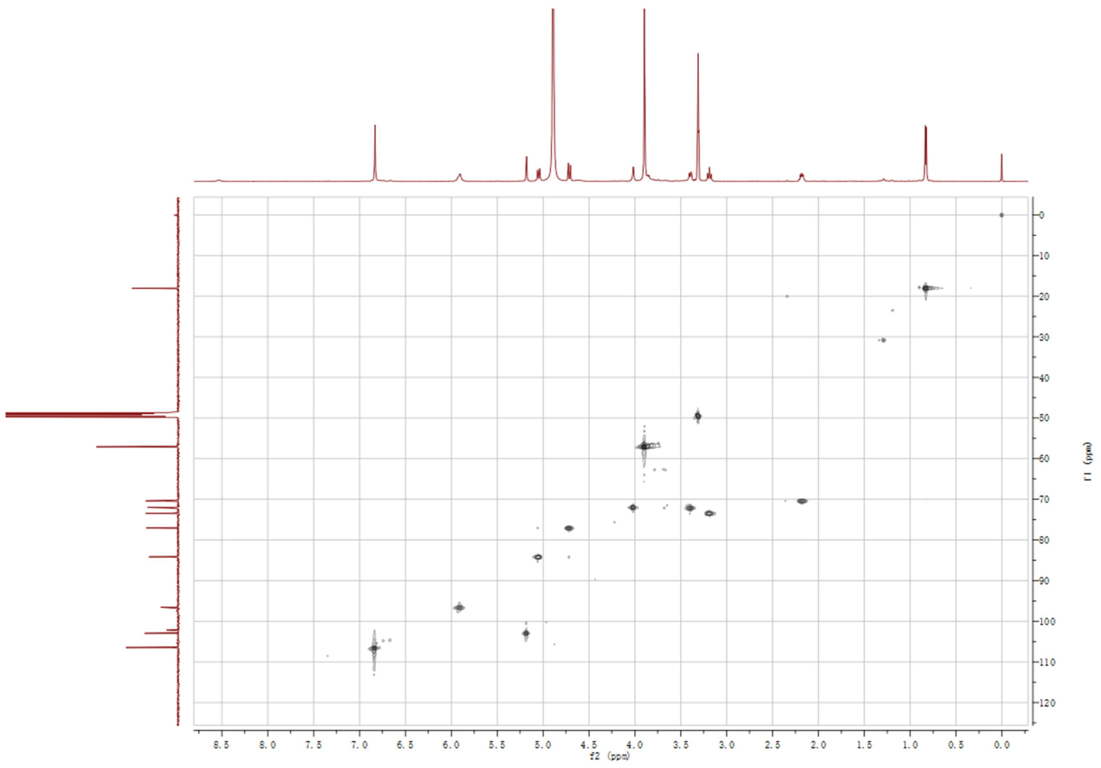

Figure S46: HSQC spectrum of compound 6

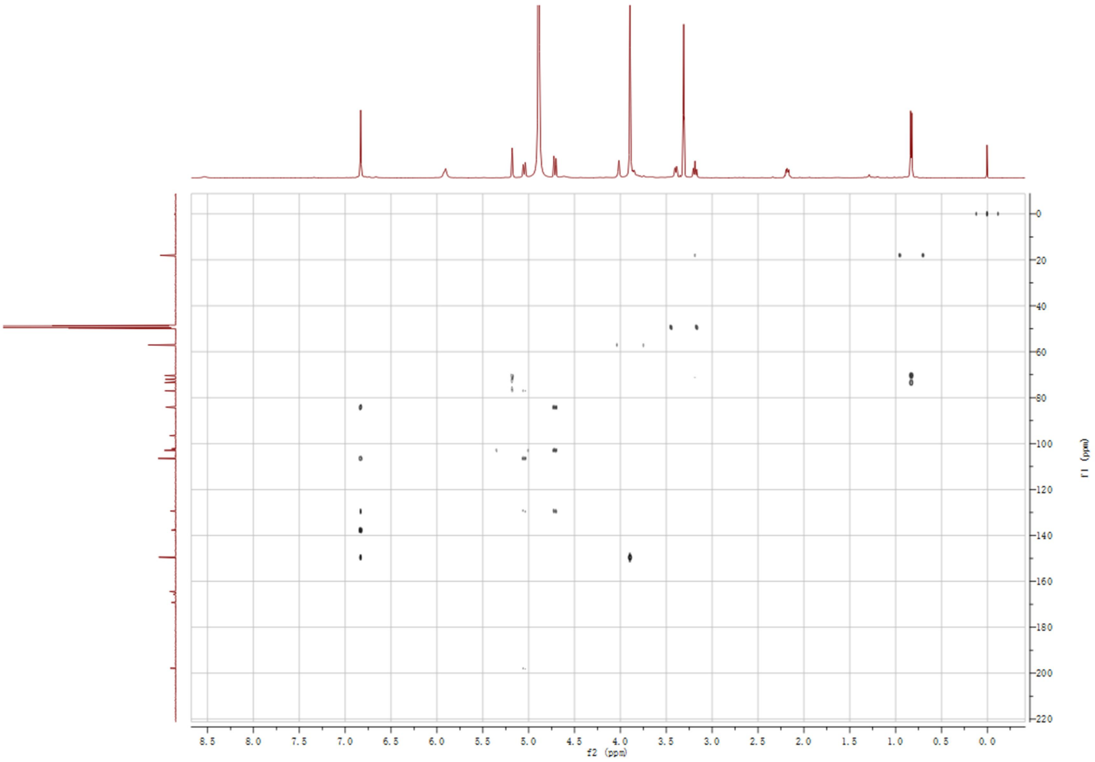

Figure S47: HMBC spectrum of compound 6

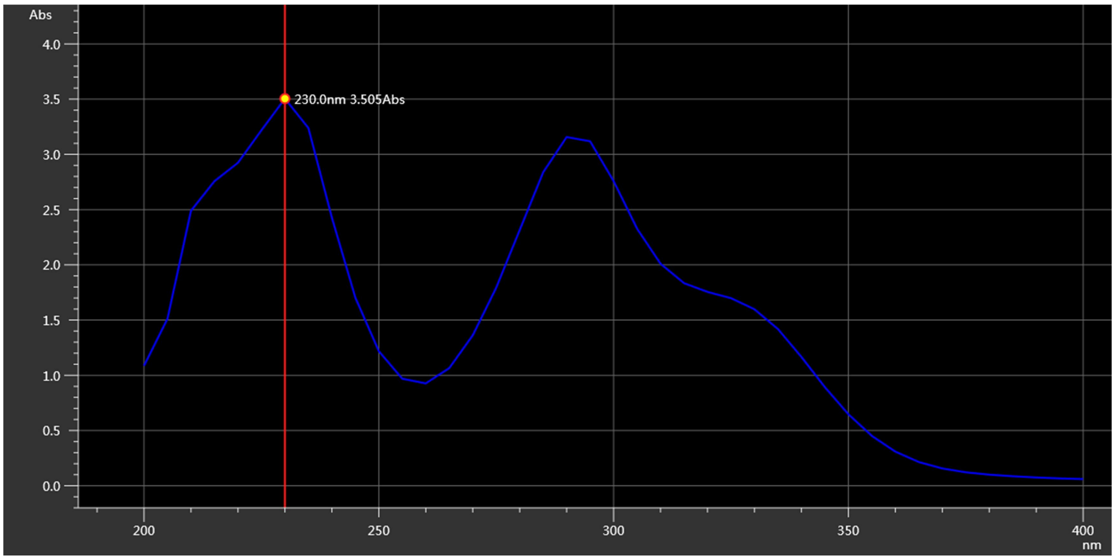

Figure S48: UV spectrum of compound 6

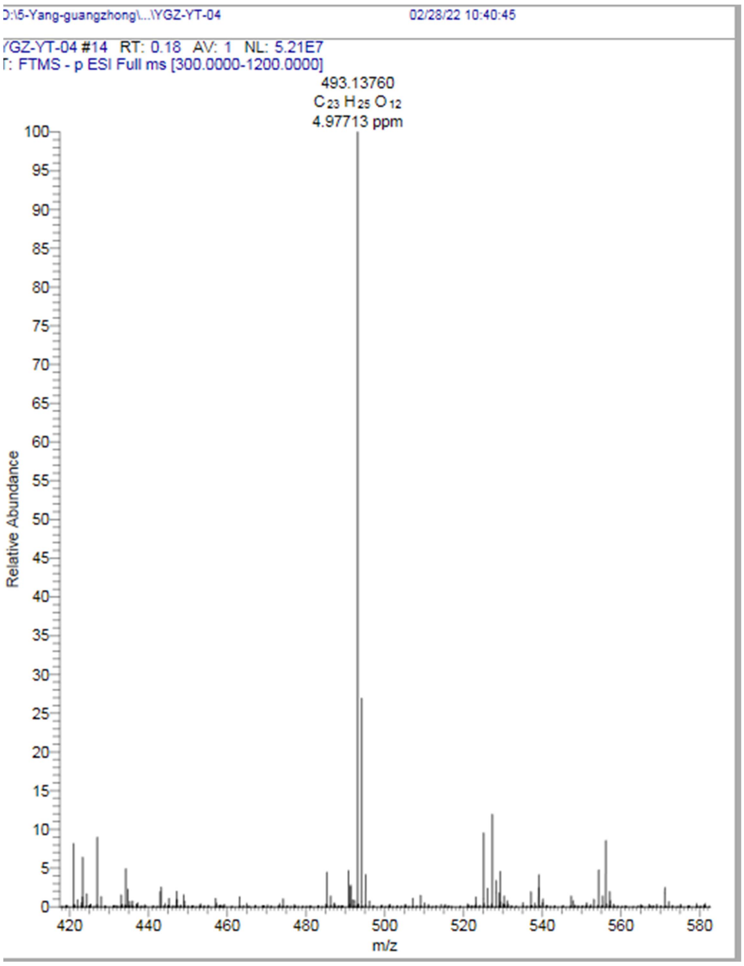

Figure S49: HR-ESI-MS of compound 6

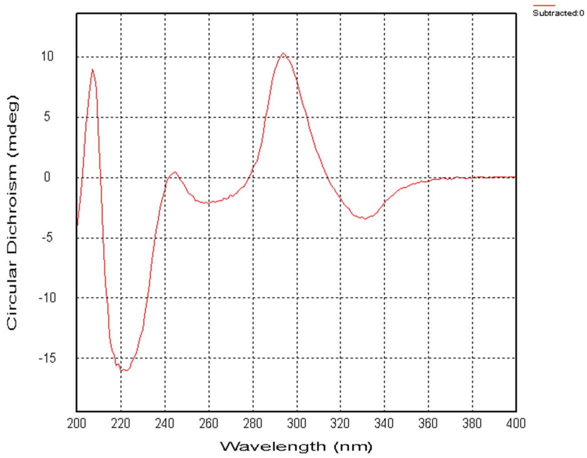

Figure S50: CD spectrum of compound 6

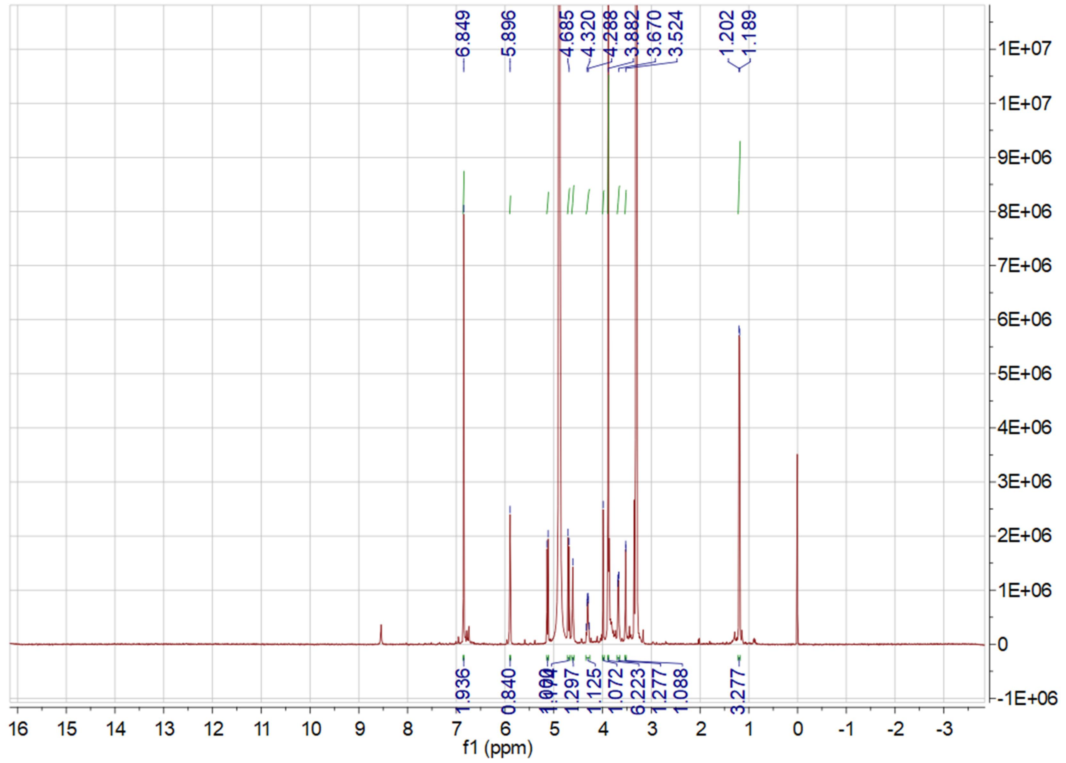

Figure S51: ^{1}H-NMR (500 MHz, CD_3OD) spectrum of compound 7

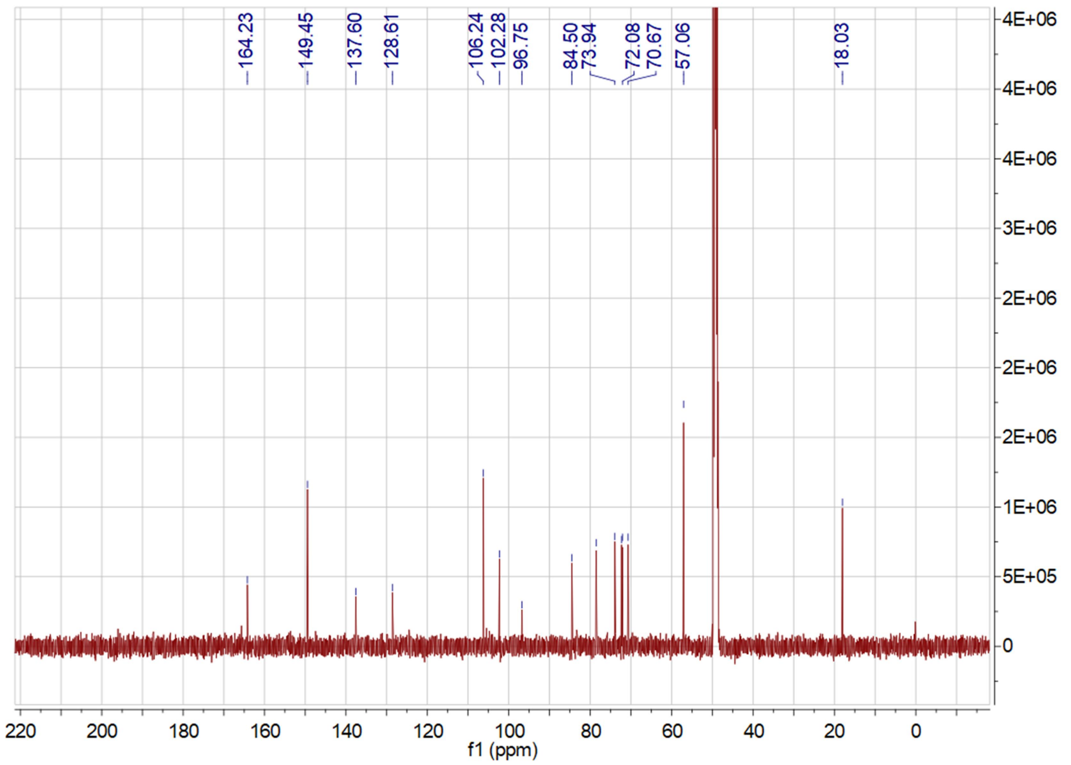

Figure S52: The ^{13}C-NMR (125 MHz, CD_3OD) spectrum of compound 7

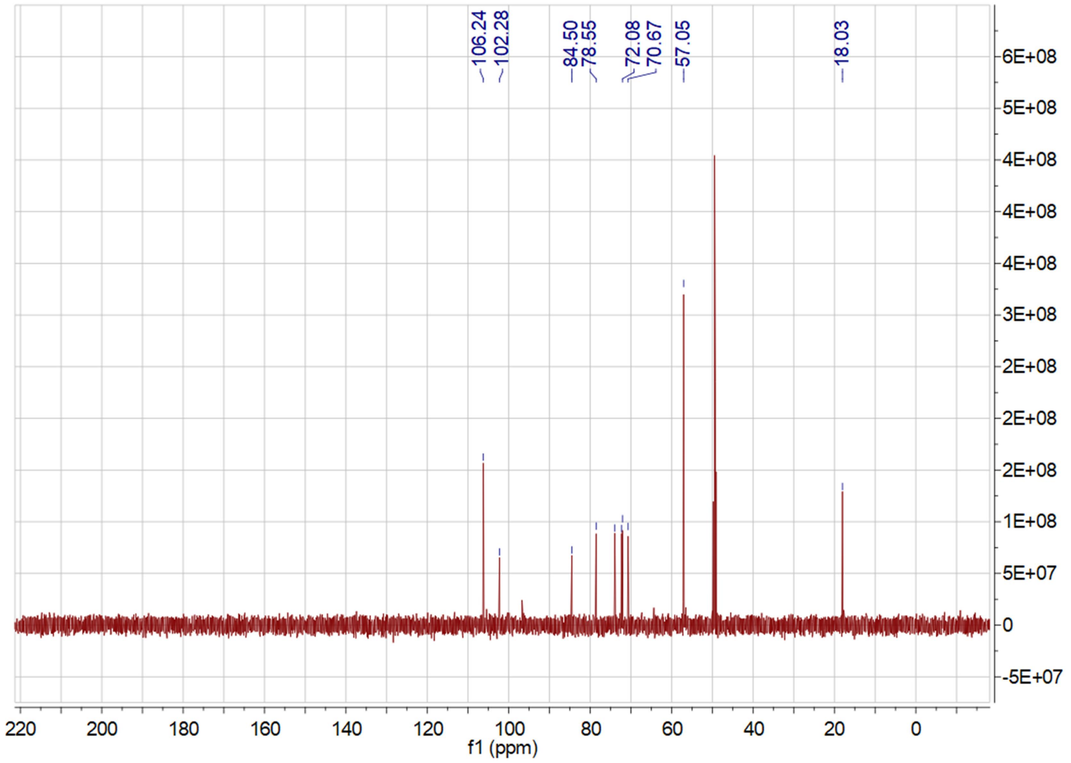

Figure S53: ^{13}C-NMR-DEPT (θ=135°) spectrum of compound 7

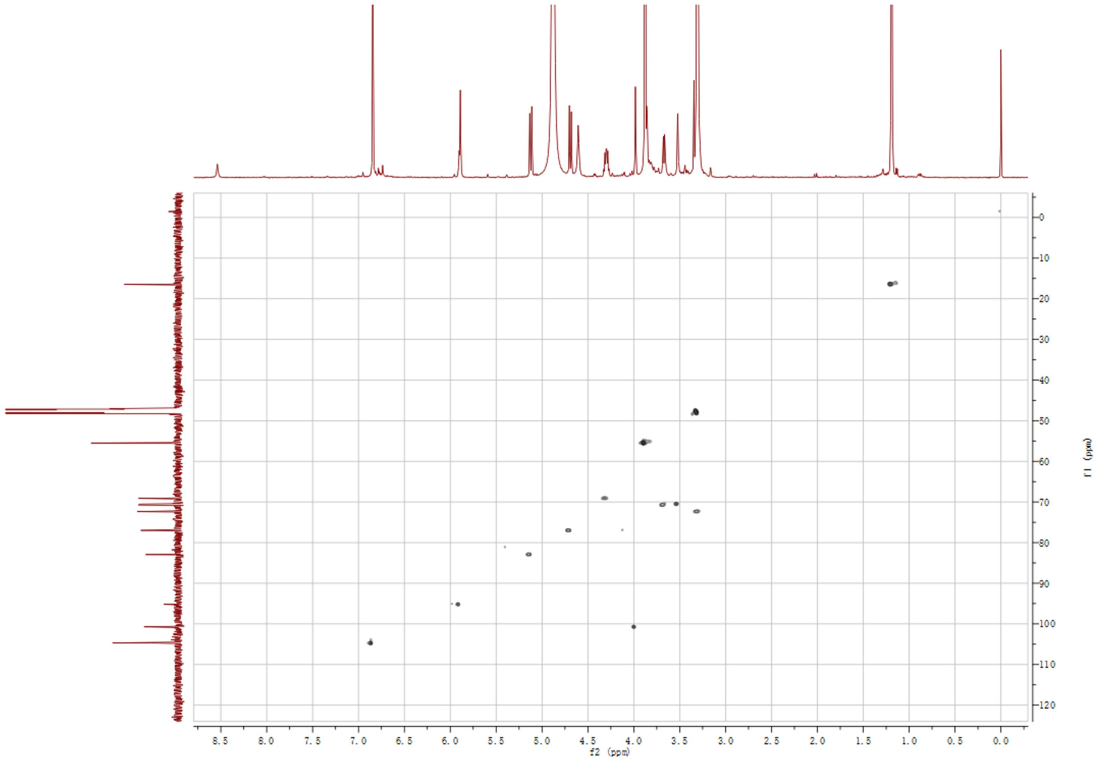

Figure S54: HSQC spectrum of compound 7

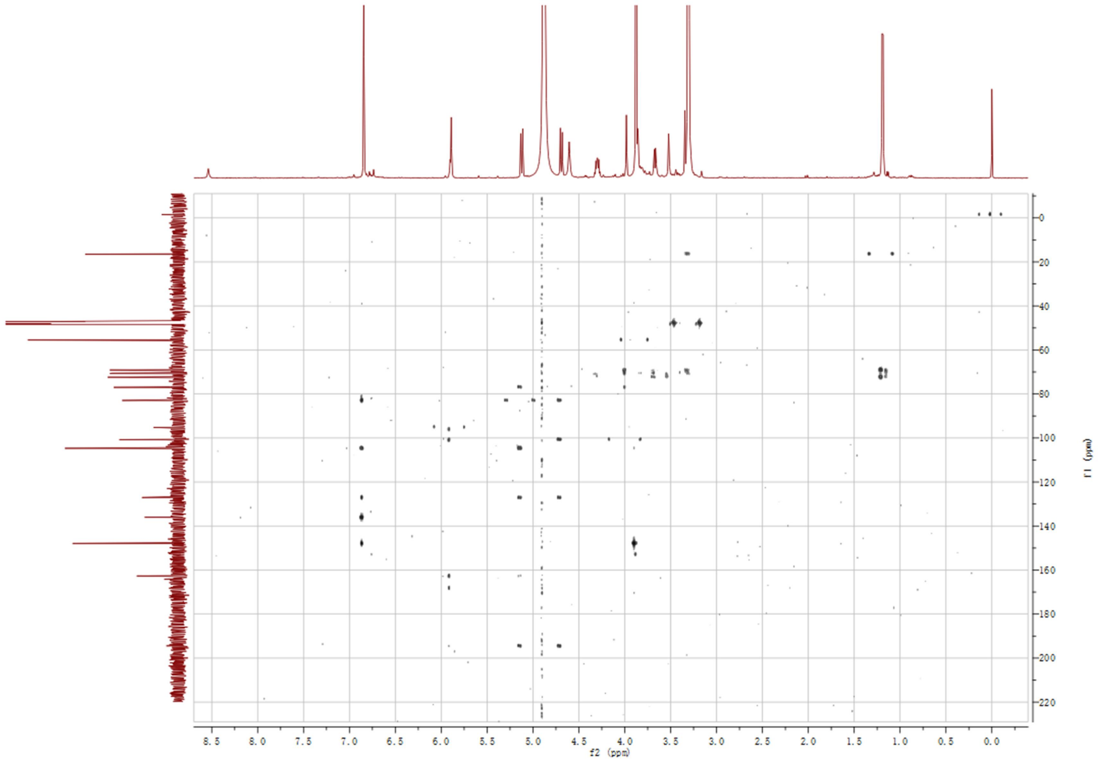

Figure S55: HMBC spectrum of compound 7

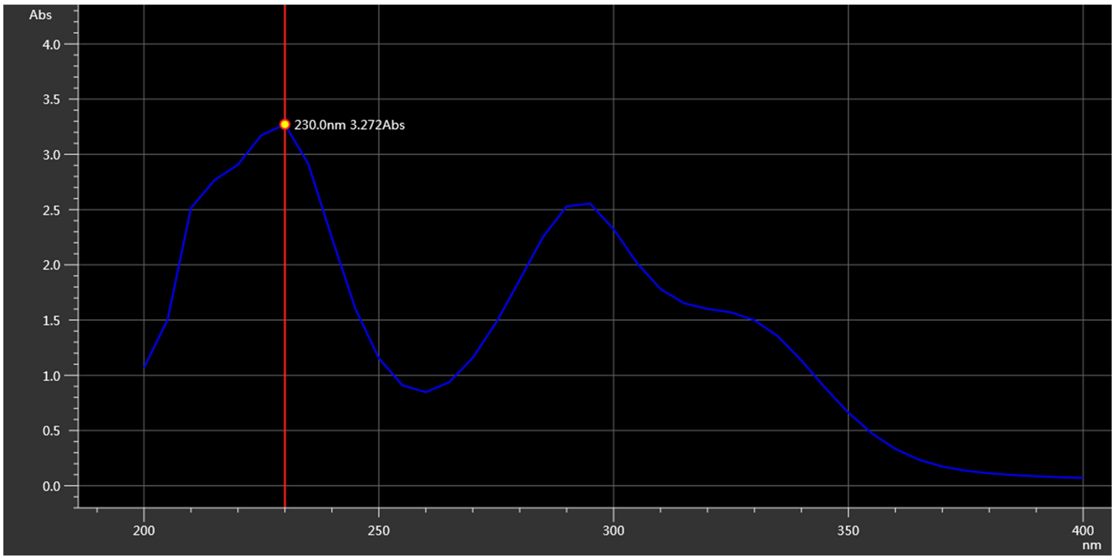

Figure S56: UV spectrum of compound 7

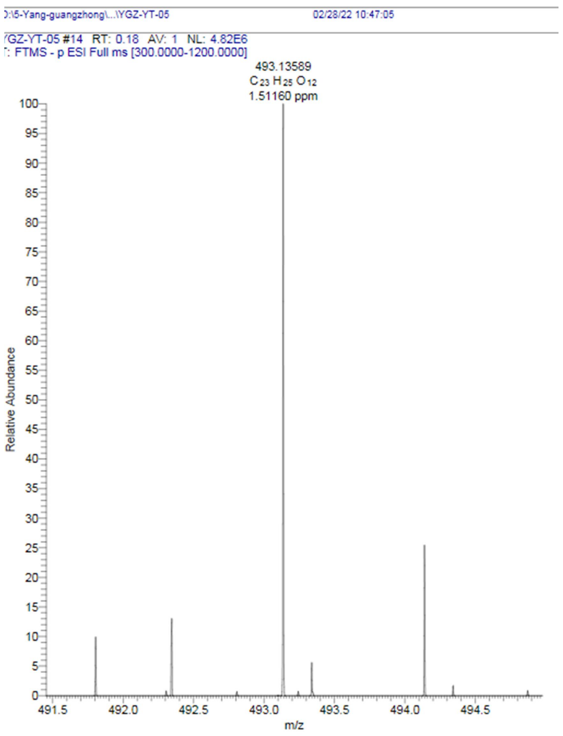

Figure S57: HR-ESI-MS of compound 7

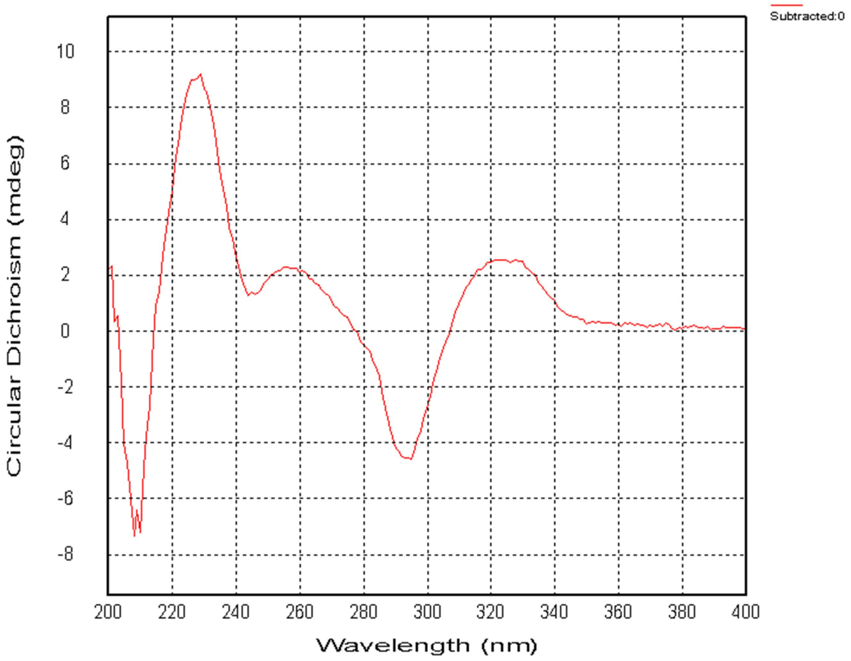

Figure S58: CD spectrum of compound 7

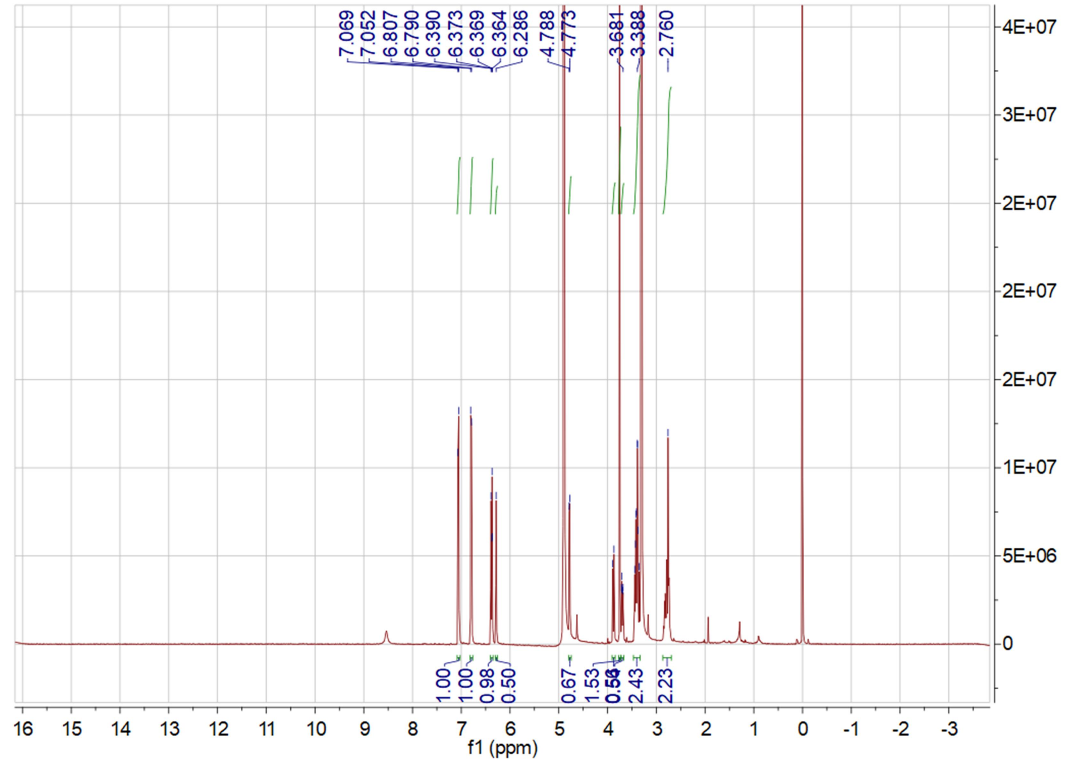

Figure S59: ^{1}H-NMR (500 MHz, CD_3OD) spectrum of compound 8

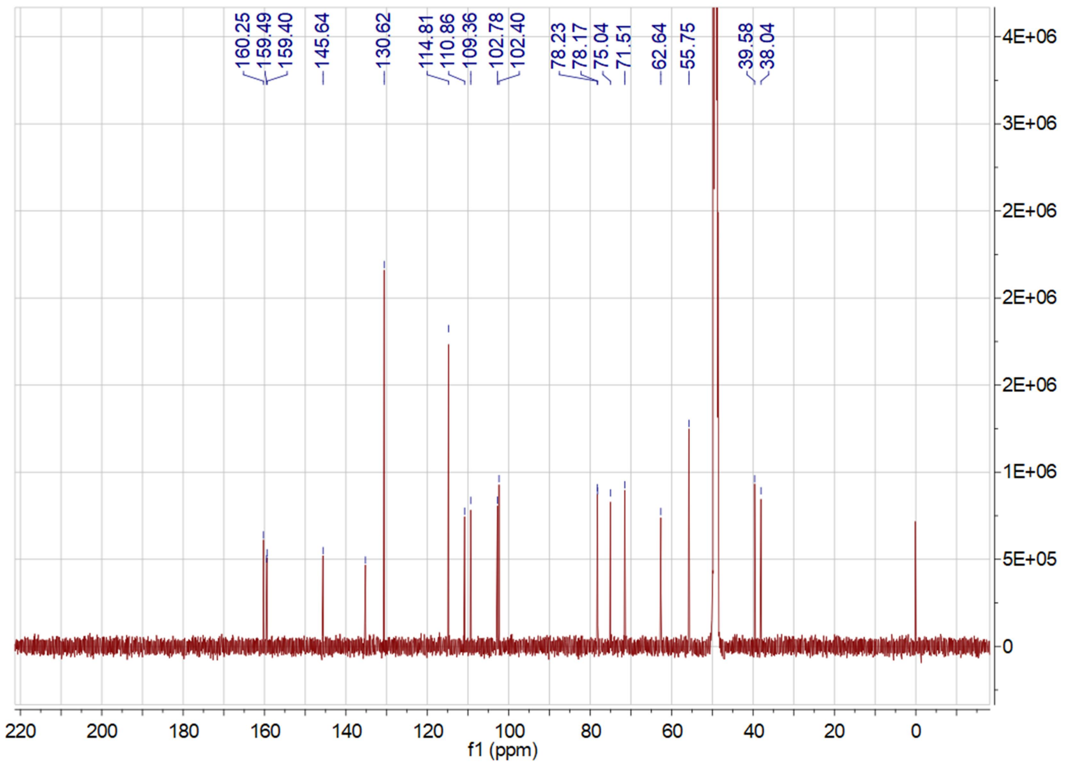

Figure S60: ^{13}C-NMR (125 MHz, CD_3OD) spectrum of compound 8

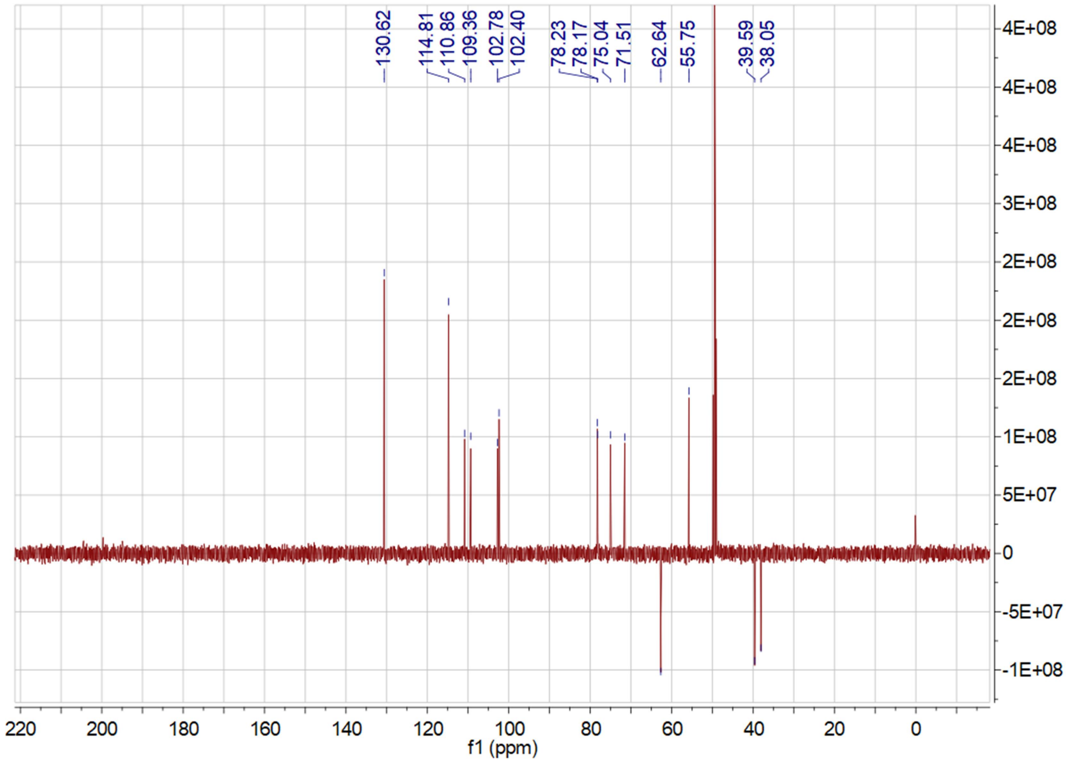

Figure S61: ^{13}C-NMR-DEPT (θ=135°) spectrum of compound 8

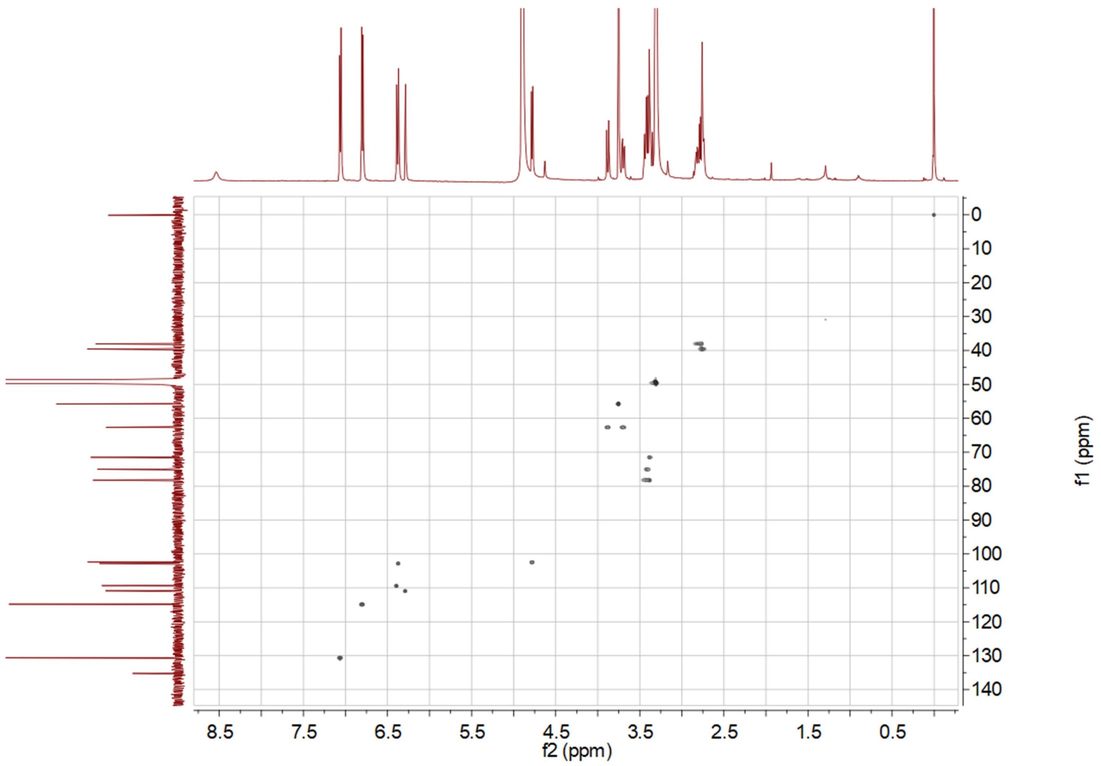

Figure S62: HSQC spectrum of compound 8

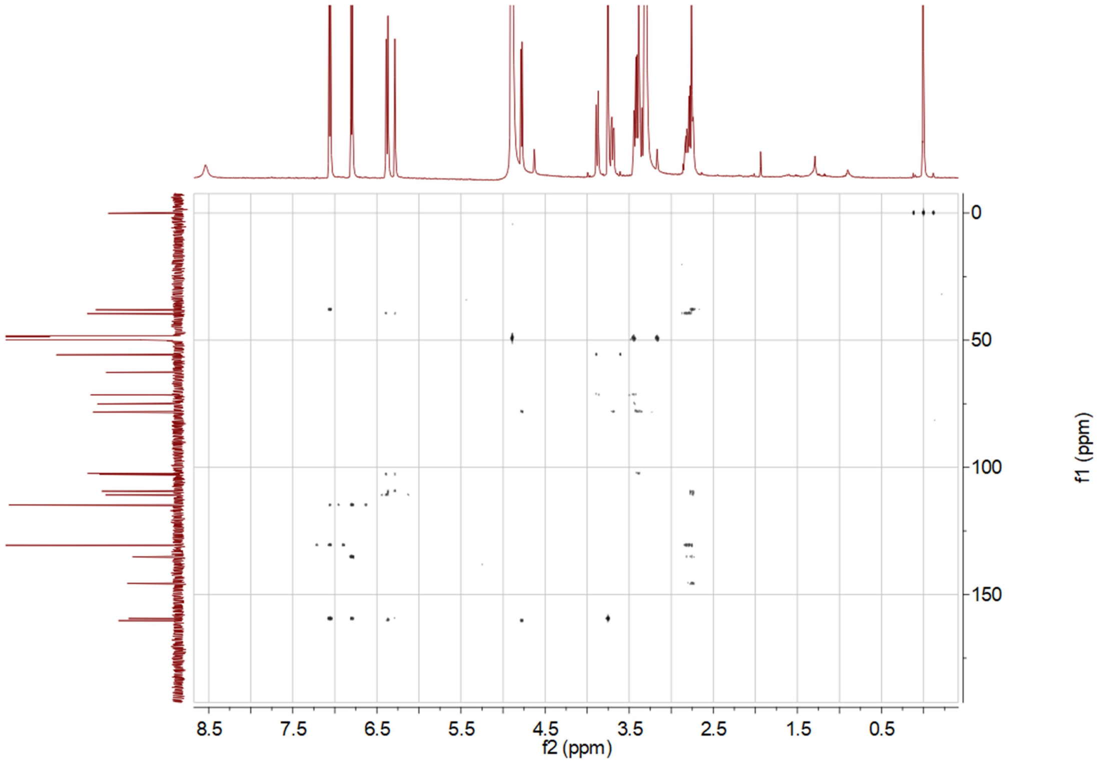

Figure S63: HMBC spectrum of compound 8

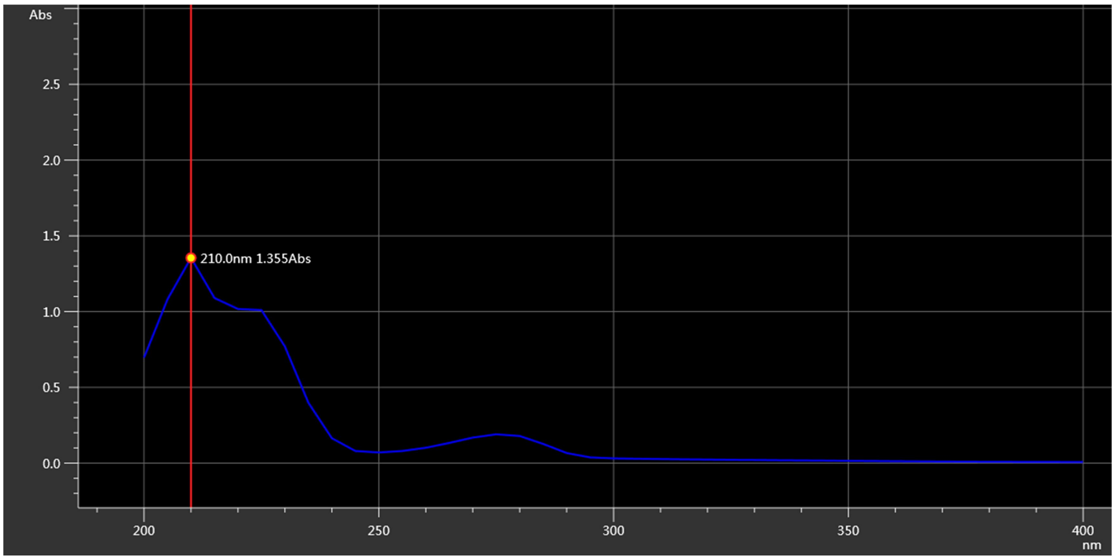

Figure S64: UV spectrum of compound 8

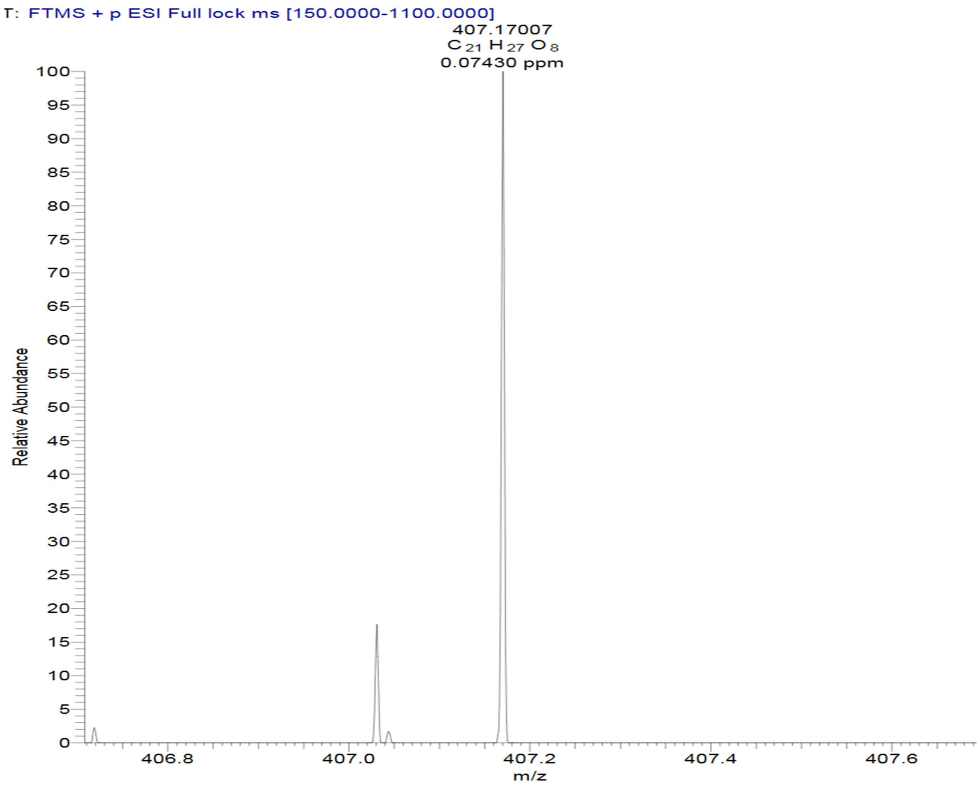

Figure S65: HR-ESI-MS of compound 8

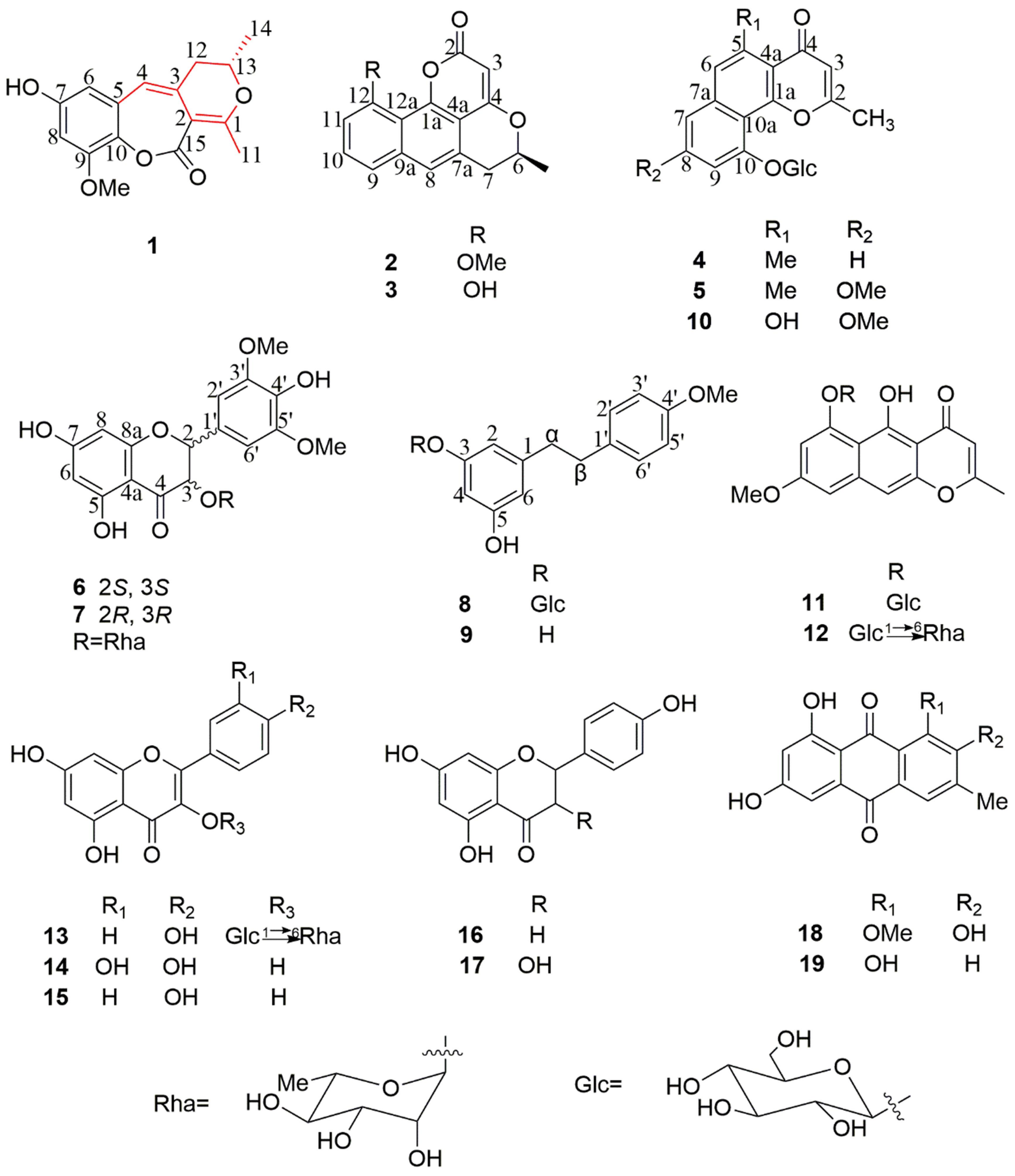

Figure S66 Phenolic compounds from *Berchemia lineata* (L.) DC.

Computational methods

Conformational analysis

Conformational analysis for compound **1-3 (Figure S67-S69**) were performed in Yinfo Cloud Platform (http://cloud.yinfotek.com/) using Stochastic algorithm by Confab [1] at MMFF94 force field with RMSD threshold of 0.5 Å and energy window of 7 kcal/mol.

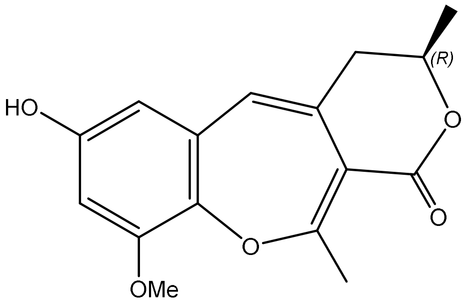

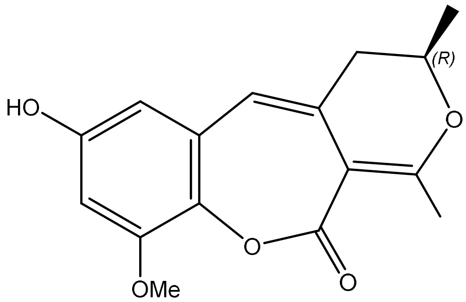

1a **1b**

Figure S67 Chemical structure of compound 1

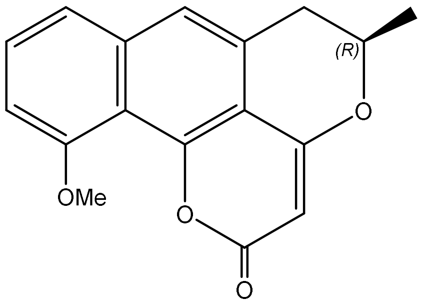

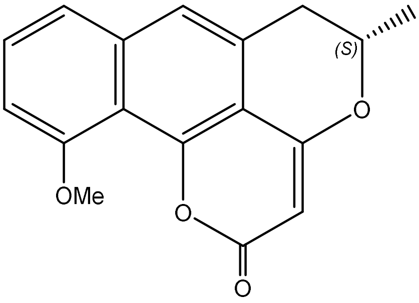

2a **2b**

Figure S68 Chemical structure of compound 2

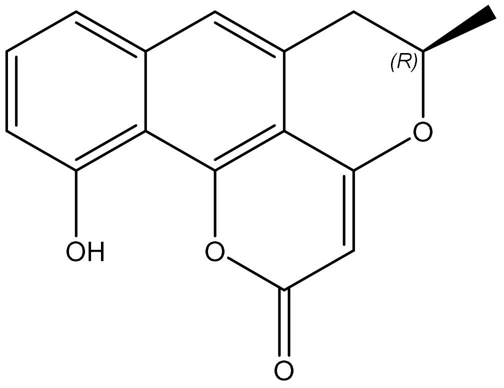

3a

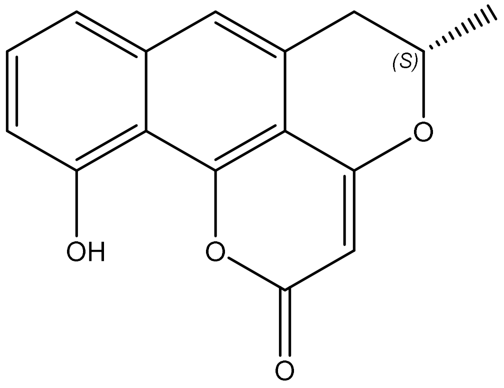

3b

Figure S69 Chemical structure of compound 3

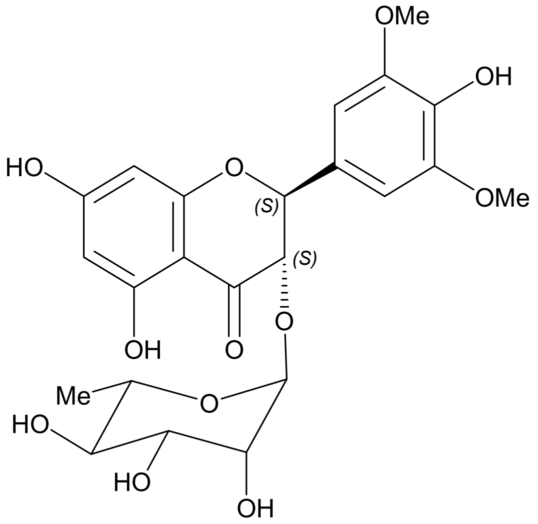

6

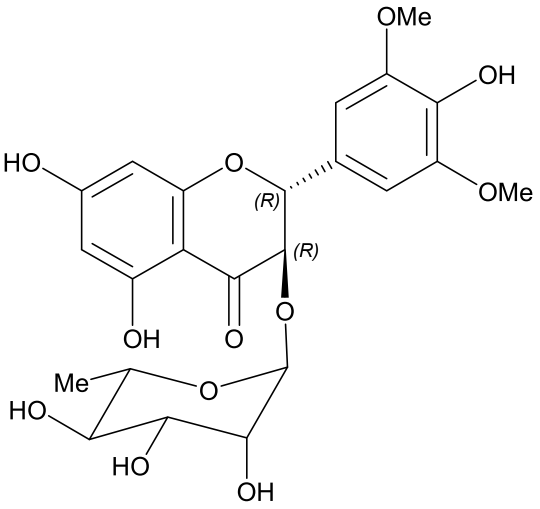

7

Figure S70 Chemical structure of compound 6 and 7

NMR calculation

The theoretical calculations were carried out using Gaussian 09 [2]. At first, all conformers were optimized at PM6. Room-temperature equilibrium populations were calculated according to Boltzmann distribution law, based on which dominative conformers of population over 1% were kept. The chosen conformers were further optimized at B3LYP/6-31G(d,p) in gas phase (**Table S1**). Vibrational frequency analysis confirmed the stable structures. NMR calculations were carried out following the protocol adapted from Michael *et al.* [3] (**Table S2**) using the Gauge-Including Atomic Orbitals (GIAO) method at mPW1PW91/6-311+G(2d,p) level in $CDCl_3$ simulated by the IEFPCM model. The TMS-corrected NMR chemical shift values were averaged according to Boltzmann distribution and fitted to the experimental values by linear regression. The calculated ^{13}C- and ^{1}H-NMR chemical shift values of TMS in $CDCl_3$ were 77.2 and 7.27 ppm respectively. To confirm the conclusions of NMR calculations, DP4+ analysis was also performed.

$$\frac{N_i}{N} = \frac{g_i e^{-\frac{E_i}{k_B T}}}{\sum g_i e^{-\frac{E_i}{k_B T}}} \quad (1)$$

where N_i is the number of conformer i with energy E_i and degeneracy g_i at temperature T, and k_B is Boltzmann constant.

ECD calculation

The structures were directly derived from the previous NMR calculations. ECD calculations were conducted at B3LYP/6-311G(d,p) level in methanol with IEFPCM model using Time-dependent Density functional theory (TD-DFT). Rotatory strengths for 30 excited states were calculated. The ECD spectrum was simulated using the ECD/UV analysis tool in Yinfo Cloud Platform (https://cloud.yinfotek.com/) by overlapping Gaussian functions for each transition according to (2).

$$\Delta\varepsilon(E)=\frac{1}{2.297\times10^{-39}}\times\frac{1}{\sqrt{2\pi\sigma}}\sum_{i}^{A}\Delta E_i R_i e^{-\left(\frac{E-E_i}{2\sigma}\right)^2} \quad \textbf{(2)}$$

where σ represents the width of the band at 1/*e* height, while ΔE_i and R_i are the excitation energies and rotatory strengths for transition *i*, respectively.

The σ and UV-shift values of compound was set 0.25 eV and -13 nm, respectively. The spectrum of the enantiomers were produced directly by mirror inversion about the horizontal axis.

References

1. Noel M OBoyle, Tim V, ermeersch, Christopher J Flynn, Anita R Maguire Maguire, and Geoffey R Hutchison. Confab - systematic generation of diverse low-energy conformers. *Journal of Cheminformatics*, 3:3–8, March 2011.
2. M. J. Frisch, G. W. Trucks, H. B. Schlegel, G. E. Scuseria, M. A. Robb, J. R. Cheeseman, G. Scalmani, V. Barone, B. Mennucci, G. A. Petersson, H. Nakatsuji, M. Caricato, X. Li, H. P. Hratchian, A. F. Izmaylov, J. Bloino, G. Zheng, J. L. Sonnenberg, M. Hada, M. Ehara, K. Toyota, R. Fukuda, J. Hasegawa, M. Ishida, T. Nakajima, Y. Honda, O. Kitao, H. Nakai, T. Vreven, J. A. Montgomery, Jr., J. E. Peralta, F. Ogliaro, M. Bearpark, J. J. Heyd, E. Brothers, K. N. Kudin, V. N. Staroverov, R. Kobayashi, J. Normand, K. Raghavachari, A. Rendell, J. C. Burant, S. S. Iyengar, J. Tomasi, M. Cossi, N. Rega, J. M. Millam, M. Klene, J. E. Knox, J. B. Cross, V. Bakken, C. Adamo, J. Jaramillo, R. Gomperts, R. E. Stratmann, O. Yazyev, A. J. Austin, R. Cammi, C. Pomelli, J. W. Ochterski, R. L. Martin, K. Morokuma, V. G. Zakrzewski, G. A. Voth, P. Salvador, J. J. Dannenberg, S. Dapprich, A. D. Daniels, O. Farkas, J. B. Foresman, J. V. Ortiz, J. Cioslowski, and D. J. Fox. Gaussian 09 Revision D.01. Gaussian Inc. Wallingford CT 2009.
3. Michael W. Lodewyk, Matthew R. Siebert, and Dean J. Tantillo. Computational Prediction of 1H and 13C Chemical Shifts: A Useful Tool for Natural Product, Mechanistic, and Synthetic Organic Chemistry. *Chem. Rev.*, **2012**, *112* (3), pp 1839–1862.

1. Energies and Coordinates

Energies at B3LYP theory level

Table S1 Energies of configurations **1-3**.

Configuration	Conformer	Structure	E (Hartree)	E (kcal/mol)	Population (%)
1a	1	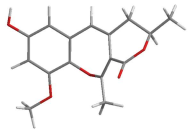	-995.4763653	-624670.8454	74.65
1a	2	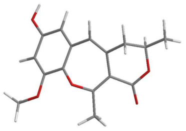	-995.4751866	-624670.1057	21.42
1a	3	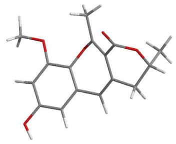	-995.473584	-624669.1001	3.92
1b	1	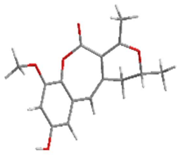	-995.4793471	-624672.7165	73.18
1b	3	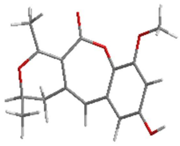	-995.4783995	-624672.1219	26.82
2a	1	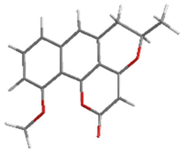	-957.39905260	-600776.97	90.8
2a	2	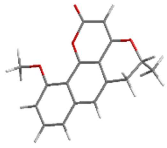	-957.39688230	-600775.61	9.2

3a	1	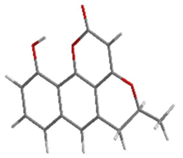	-918.09903860	-576115.84	89.8
3a	2	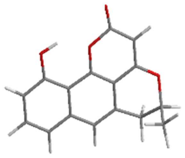	-918.09698460	-576114.55	10.2
6	1	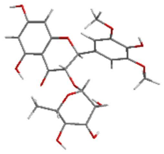	-1795.24537650	-1126533.47	100.0
7	2	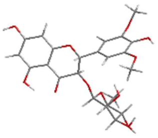	-1795.25151310	-1126537.32	73.0
7	3	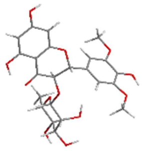	-1795.25039640	-1126536.62	22.4
7	1	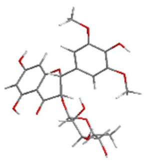	-1795.24889550	-1126535.68	4.6

Coordinates at B3LYP theory level

Table S2 Standard orientations of configurations **1-3,6 and 7**.

Conformer 1a-1

Center	Atomic	Atomic	Coordinates (Angstroms)		
Number	Number	Type	X	Y	Z
1	6	0	1.390855	0.053013	-0.497656
2	6	0	1.016253	-1.270116	-0.263642
3	6	0	1.989212	-2.181354	0.191979
4	6	0	3.289895	-1.749602	0.429096
5	6	0	3.653163	-0.411113	0.226031
6	6	0	2.699134	0.499912	-0.230289
7	8	0	0.495909	0.936333	-1.101710
8	6	0	-0.529780	1.366894	-0.278468
9	6	0	-1.497155	0.484799	0.094479
10	6	0	-1.479960	-0.943530	-0.270817
11	6	0	-0.368098	-1.696316	-0.446997
12	6	0	-0.459457	2.838772	-0.033234
13	8	0	4.278266	-2.586134	0.873144
14	6	0	-2.674360	0.973276	0.881233
15	8	0	-3.862896	0.359959	0.625545
16	6	0	-3.944346	-0.560030	-0.490452
17	6	0	-2.846796	-1.604471	-0.334049
18	8	0	-2.643352	1.847682	1.719778
19	6	0	-5.354012	-1.127124	-0.477663

20	8	0	2.940648	1.817755	-0.464663
21	6	0	4.255425	2.305539	-0.238526
22	1	0	1.708832	-3.215966	0.378589
23	1	0	4.677662	-0.120632	0.420447
24	1	0	-0.510378	-2.759239	-0.640539
25	1	0	-0.323782	3.345543	-0.996667
26	1	0	0.434326	3.060513	0.562935
27	1	0	-1.334745	3.220088	0.487603
28	1	0	3.910112	-3.477247	0.976372
29	1	0	-3.779364	0.013205	-1.413476
30	1	0	-3.045414	-2.168191	0.589283
31	1	0	-2.885505	-2.321025	-1.162815
32	1	0	-5.503087	-1.792135	-1.335316
33	1	0	-6.090641	-0.320040	-0.530636
34	1	0	-5.531872	-1.695629	0.441384
35	1	0	4.223535	3.368090	-0.485195
36	1	0	4.555493	2.183316	0.810205
37	1	0	4.986380	1.803290	-0.885096

Conformer 1a-2

Center	Atomic	Atomic	Coordinates (Angstroms)		
Number	Number	Type	X	Y	Z
1	6	0	-1.405986	0.079340	-0.517097
2	6	0	-0.936711	-1.228467	-0.401897
3	6	0	-1.807123	-2.226690	0.075925

4	6	0	-3.105280	-1.894061	0.449013
5	6	0	-3.564704	-0.572651	0.360217
6	6	0	-2.711845	0.423831	-0.117501
7	8	0	-0.616464	1.050247	-1.129940
8	6	0	0.444526	1.511591	-0.367975
9	6	0	1.488264	0.668238	-0.121765
10	6	0	1.511343	-0.717183	-0.637322
11	6	0	0.445747	-1.542283	-0.753353
12	6	0	0.270075	2.956183	-0.033436
13	8	0	-3.998996	-2.816465	0.922528
14	6	0	2.692532	1.156467	0.628377
15	8	0	3.767535	0.324734	0.738641
16	6	0	3.765282	-1.055813	0.312384
17	6	0	2.900038	-1.224142	-0.931863
18	8	0	2.800708	2.249404	1.144791
19	6	0	5.223718	-1.440333	0.112658
20	8	0	-3.052695	1.733601	-0.249553
21	6	0	-4.375851	2.117785	0.096247
22	1	0	-1.451096	-3.250685	0.168362
23	1	0	-4.583491	-0.361603	0.659300
24	1	0	0.633069	-2.566275	-1.073687
25	1	0	0.026638	3.491116	-0.960114
26	1	0	1.147518	3.382163	0.443477
27	1	0	-0.604083	3.065496	0.620913

28	1	0	-3.571573	-3.686697	0.937207
29	1	0	3.333619	-1.648937	1.130835
30	1	0	3.355065	-0.653111	-1.754483
31	1	0	2.879429	-2.277959	-1.229162
32	1	0	5.666286	-0.858970	-0.703189
33	1	0	5.305965	-2.505625	-0.129838
34	1	0	5.796361	-1.245284	1.024227
35	1	0	-4.432343	3.190536	-0.095589
36	1	0	-5.118700	1.597411	-0.521682
37	1	0	-4.587527	1.925388	1.156015

Conformer 1a-3

Center	Atomic	Atomic	Coordinates (Angstroms)		
Number	Number	Type	X	Y	Z
1	6	0	-1.301606	0.079742	-0.459376
2	6	0	-1.026109	-1.268076	-0.231509
3	6	0	-2.075537	-2.117754	0.170316
4	6	0	-3.353327	-1.602328	0.360602
5	6	0	-3.617838	-0.239933	0.163918
6	6	0	-2.586920	0.610601	-0.239184
7	8	0	-0.325680	0.905680	-1.015724
8	6	0	0.697837	1.259203	-0.153078
9	6	0	1.591185	0.310701	0.243672
10	6	0	1.486493	-1.111167	-0.129318
11	6	0	0.333433	-1.783573	-0.361204

12	6	0	0.716126	2.731942	0.097640
13	8	0	-4.413112	-2.376091	0.750436
14	6	0	2.790538	0.726221	1.040371
15	8	0	3.944961	0.041193	0.813917
16	6	0	4.002827	-0.957967	-0.236174
17	6	0	2.795195	-1.881313	-0.098250
18	8	0	2.805026	1.615856	1.863627
19	6	0	4.171507	-0.306020	-1.607418
20	8	0	-2.729558	1.944249	-0.464114
21	6	0	-4.017823	2.516617	-0.289563
22	1	0	-1.872699	-3.171038	0.352651
23	1	0	-4.628133	0.115975	0.320265
24	1	0	0.411369	-2.853619	-0.551908
25	1	0	0.633632	3.248989	-0.866286
26	1	0	-0.175790	3.007100	0.674277
27	1	0	1.600778	3.055717	0.640617
28	1	0	-4.110507	-3.291583	0.853039
29	1	0	4.913265	-1.515159	0.003442
30	1	0	2.811181	-2.643607	-0.885528
31	1	0	2.888369	-2.411014	0.860350
32	1	0	3.274781	0.240635	-1.913474
33	1	0	4.377110	-1.074210	-2.361659
34	1	0	5.014953	0.390990	-1.588019
35	1	0	-3.904860	3.576681	-0.522683

36	1	0	-4.371139	2.406199	0.743801
37	1	0	-4.751282	2.069468	-0.972688

Conformer 1b-1

Center	Atomic	Atomic	Coordinates (Angstroms)		
Number	Number	Type	X	Y	Z
1	6	0	-1.442856	0.162072	-0.439455
2	6	0	-1.019508	-1.169821	-0.420501
3	6	0	-1.922460	-2.172270	-0.020530
4	6	0	-3.211654	-1.836574	0.371630
5	6	0	-3.631633	-0.499176	0.375724
6	6	0	-2.748828	0.505082	-0.033399
7	8	0	-0.687957	1.183706	-0.989932
8	6	0	0.518452	1.627354	-0.480298
9	6	0	1.530419	0.606987	-0.131679
10	6	0	1.456454	-0.781625	-0.618324
11	6	0	0.348712	-1.539765	-0.764531
12	8	0	0.702800	2.823834	-0.465383
13	8	0	-4.043136	-2.855329	0.756927
14	6	0	2.686111	1.028806	0.487464
15	8	0	3.723614	0.209427	0.752720
16	6	0	3.669722	-1.195061	0.389918
17	6	0	2.821001	-1.381096	-0.865033
18	6	0	3.001596	2.413913	0.975841
19	6	0	5.112590	-1.654065	0.252383

20	8	0	-3.061407	1.825459	-0.085048
21	6	0	-4.370318	2.223970	0.284024
22	1	0	-1.614555	-3.212514	0.000320
23	1	0	-4.642310	-0.253870	0.685937
24	1	0	0.484976	-2.569871	-1.089541
25	1	0	-4.904803	-2.484046	1.000181
26	1	0	3.187241	-1.712201	1.230139
27	1	0	3.321777	-0.882139	-1.707362
28	1	0	2.750679	-2.447641	-1.101901
29	1	0	3.844263	2.357298	1.669334
30	1	0	2.139342	2.875345	1.459052
31	1	0	3.267088	3.066075	0.137456
32	1	0	5.601369	-1.147302	-0.586495
33	1	0	5.148734	-2.734567	0.077088
34	1	0	5.673904	-1.432711	1.165358
35	1	0	-4.589806	1.975180	1.331293
36	1	0	-5.130279	1.766107	-0.363449
37	1	0	-4.395734	3.307693	0.158911

Conformer 1b-3

Center	Atomic	Atomic	Coordinates (Angstroms)		
Number	Number	Type	X	Y	Z
1	6	0	1.342825	0.242344	-0.465431
2	6	0	0.945589	-1.095756	-0.542942
3	6	0	1.854597	-2.105236	-0.175470

4	6	0	3.123470	-1.772171	0.279996
5	6	0	3.516422	-0.430388	0.381180
6	6	0	2.627885	0.581851	0.005615
7	8	0	0.586313	1.283360	-0.975890
8	6	0	-0.643607	1.672590	-0.475850
9	6	0	-1.642476	0.612840	-0.224376
10	6	0	-1.528350	-0.737052	-0.800536
11	6	0	-0.403214	-1.467808	-0.955700
12	8	0	-0.852459	2.861857	-0.389624
13	8	0	3.961923	-2.798101	0.629269
14	6	0	-2.818842	0.964456	0.400780
15	8	0	-3.846352	0.111275	0.582176
16	6	0	-3.779018	-1.255836	0.093664
17	6	0	-2.875200	-1.330647	-1.138754
18	6	0	-3.166649	2.301901	0.990198
19	6	0	-3.357868	-2.168197	1.241007
20	8	0	2.916297	1.908090	0.047130
21	6	0	4.202976	2.305014	0.489050
22	1	0	1.566644	-3.149996	-0.229819
23	1	0	4.511551	-0.187028	0.739481
24	1	0	-0.509129	-2.476014	-1.352615
25	1	0	4.807875	-2.427594	0.923566
26	1	0	-4.813993	-1.475191	-0.187819
27	1	0	-3.346215	-0.765401	-1.954630

28	1	0	-2.785799	-2.372522	-1.463135
29	1	0	-4.009974	2.174598	1.673527
30	1	0	-3.446918	3.007773	0.201366
31	1	0	-2.317114	2.746286	1.510237
32	1	0	-3.440081	-3.216991	0.933254
33	1	0	-4.007059	-2.010586	2.107590
34	1	0	-2.322114	-1.977429	1.537637
35	1	0	4.994375	1.904881	-0.159033
36	1	0	4.390390	1.991010	1.524930
37	1	0	4.210542	3.394926	0.436404

Conformer 2a-1

Center	Atomic	Atomic	Coordinates (Angstroms)		
Number	Number	Type	X	Y	Z
1	6	0	-1.369835	-0.511326	-0.014183
2	6	0	-1.023449	-1.905032	-0.055071
3	6	0	-2.043652	-2.888536	-0.035500
4	6	0	-3.365870	-2.516837	0.020491
5	6	0	-3.732202	-1.157621	0.061440
6	6	0	-2.765289	-0.161892	0.045725
7	6	0	-0.291387	0.432725	-0.034578
8	6	0	1.036720	0.001692	-0.070490
9	6	0	1.356452	-1.383309	-0.141866
10	6	0	0.343554	-2.302570	-0.124761
11	6	0	2.075436	1.005106	-0.064902

12	8	0	3.376698	0.632747	-0.062144
13	6	0	3.687861	-0.695914	0.433740
14	6	0	2.818247	-1.738079	-0.269083
15	8	0	-0.581764	1.751746	-0.027766
16	6	0	0.396331	2.774145	-0.085478
17	6	0	1.766125	2.331183	-0.085062
18	8	0	-0.010853	3.911890	-0.105428
19	6	0	5.178977	-0.887182	0.217688
20	8	0	-3.063163	1.156724	0.089582
21	6	0	-4.423233	1.557401	0.151961
22	1	0	-1.760234	-3.936614	-0.068142
23	1	0	-4.146468	-3.272737	0.033977
24	1	0	-4.781966	-0.896438	0.107328
25	1	0	0.568097	-3.365431	-0.172984
26	1	0	3.459105	-0.701821	1.509087
27	1	0	3.017777	-2.730239	0.151864
28	1	0	3.106616	-1.774640	-1.329986
29	1	0	2.538038	3.090160	-0.082980
30	1	0	5.499597	-1.850378	0.628604
31	1	0	5.419119	-0.865266	-0.850597
32	1	0	5.742507	-0.092161	0.714797
33	1	0	-4.912742	1.178521	1.058555
34	1	0	-4.403055	2.647696	0.176671
35	1	0	-4.982450	1.220764	-0.730578

Conformer 2a-2					
Center	Atomic	Atomic	Coordinates (Angstroms)		
Number	Number	Type	X	Y	Z
1	6	0	1.300097	-0.476213	-0.057425
2	6	0	0.999881	-1.878351	-0.145925
3	6	0	2.047254	-2.831209	-0.087787
4	6	0	3.352137	-2.421472	0.050687
5	6	0	3.673399	-1.053193	0.138677
6	6	0	2.678471	-0.086581	0.087546
7	6	0	0.195348	0.435782	-0.117353
8	6	0	-1.113914	-0.034128	-0.241794
9	6	0	-1.385672	-1.425627	-0.363043
10	6	0	-0.348033	-2.314933	-0.303328
11	6	0	-2.185186	0.935088	-0.256192
12	8	0	-3.473696	0.529124	-0.331850
13	6	0	-3.785953	-0.834021	0.065918
14	6	0	-2.822587	-1.816020	-0.609300
15	8	0	0.443071	1.761790	-0.057658
16	6	0	-0.563989	2.754998	-0.136434
17	6	0	-1.916559	2.270143	-0.217820
18	8	0	-0.192719	3.904694	-0.103177
19	6	0	-3.825149	-0.939214	1.588931
20	8	0	2.932459	1.239159	0.172518
21	6	0	4.273644	1.679538	0.318954

22	1	0	1.798805	-3.886336	-0.157492
23	1	0	4.153752	-3.153920	0.093760
24	1	0	4.710363	-0.761714	0.248528
25	1	0	-0.536291	-3.382496	-0.388450
26	1	0	-4.793481	-0.979642	-0.332721
27	1	0	-3.020924	-2.834014	-0.255459
28	1	0	-3.021839	-1.808838	-1.690098
29	1	0	-2.711549	3.004844	-0.229696
30	1	0	-4.488748	-0.173740	2.002007
31	1	0	-2.830560	-0.810347	2.027868
32	1	0	-4.204453	-1.923327	1.886570
33	1	0	4.891712	1.378213	-0.536716
34	1	0	4.217728	2.767994	0.362688
35	1	0	4.723438	1.297657	1.244667

Conformer 3a-1

Center	Atomic	Atomic	Coordinates (Angstroms)		
Number	Number	Type	X	Y	Z
1	6	0	3.937179	-1.879155	0.049794
2	6	0	4.095796	-0.486257	0.091594
3	6	0	2.991607	0.354957	0.068494
4	6	0	1.673100	-0.206927	0.004094
5	6	0	1.528882	-1.638225	-0.041906
6	6	0	2.681672	-2.453040	-0.016506
7	6	0	0.477309	0.566988	-0.015706

8	6	0	-0.779898	-0.022597	-0.063106
9	6	0	-0.905716	-1.440795	-0.143906
10	6	0	0.223475	-2.213309	-0.122806
11	6	0	-1.935487	0.840117	-0.061206
12	8	0	-3.173794	0.303633	-0.067306
13	6	0	-3.313411	-1.064766	0.411594
14	6	0	-2.308122	-1.980078	-0.288306
15	8	0	0.599526	1.923286	0.004894
16	6	0	-0.513163	2.827500	-0.056706
17	6	0	-1.802771	2.199816	-0.074006
18	6	0	-4.764516	-1.443648	0.175494
19	8	0	-0.234549	4.000796	-0.063706
20	8	0	3.234223	1.683954	0.112994
21	1	0	4.820471	-2.511666	0.068694
22	1	0	5.079802	-0.032269	0.142494
23	1	0	2.564959	-3.532238	-0.051906
24	1	0	0.142961	-3.296308	-0.177206
25	1	0	-3.097511	-1.047768	1.488894
26	1	0	-2.384635	-2.991477	0.126294
27	1	0	-2.579023	-2.047275	-1.352206
28	1	0	-2.670063	2.847327	-0.075906
29	1	0	-4.962828	-2.442245	0.578394
30	1	0	-4.993116	-1.446945	-0.895306
31	1	0	-5.431307	-0.731139	0.669594

32	1	0	2.391429	2.175164	0.081694

Conformer 3a-2

Center	Atomic	Atomic	Coordinates (Angstroms)		
Number	Number	Type	X	Y	Z
1	6	0	-3.970712	-1.613477	0.110912
2	6	0	-4.023204	-0.215577	0.214312
3	6	0	-2.863100	0.545017	0.152512
4	6	0	-1.596503	-0.106890	-0.016788
5	6	0	-1.561211	-1.541391	-0.124588
6	6	0	-2.767216	-2.272384	-0.055988
7	6	0	-0.349900	0.580603	-0.082688
8	6	0	0.854197	-0.094704	-0.234788
9	6	0	0.869789	-1.513304	-0.379788
10	6	0	-0.309215	-2.204398	-0.311788
11	6	0	2.071501	0.679489	-0.255088
12	8	0	3.266297	0.061583	-0.353688
13	6	0	3.344190	-1.348618	0.009212
14	6	0	2.214185	-2.137612	-0.662688
15	8	0	-0.370592	1.939003	0.003512
16	6	0	0.802513	2.762696	-0.080688
17	6	0	2.038609	2.044789	-0.195588
18	6	0	3.392489	-1.490018	1.528512
19	8	0	0.612019	3.951897	-0.020488
20	8	0	-3.003192	1.885017	0.262012

21	1	0	-4.895815	-2.181272	0.162512
22	1	0	-4.966101	0.304928	0.344512
23	1	0	-2.732822	-3.354784	-0.138788
24	1	0	-0.312921	-3.286998	-0.414488
25	1	0	4.304188	-1.653423	-0.414988
26	1	0	2.392485	-2.143913	-1.747088
27	1	0	2.244779	-3.181012	-0.329388
28	1	0	2.950512	2.627784	-0.213088
29	1	0	4.181392	-0.854523	1.941512
30	1	0	2.440790	-1.208513	1.990612
31	1	0	3.608283	-2.529419	1.799812
32	1	0	-2.129490	2.315813	0.198112

Conformer 6-1

Center	Atomic	Atomic	Coordinates (Angstroms)		
Number	Number	Type	X	Y	Z
1	6	0	-1.125630	3.269390	1.829297
2	6	0	-1.533236	3.925586	0.509797
3	6	0	-2.842130	3.289975	0.042397
4	6	0	-2.632417	1.795876	-0.304303
5	6	0	-1.481111	1.178387	0.549497
6	8	0	-1.265017	1.816389	1.766797
7	8	0	-2.384216	1.657879	-1.705603
8	8	0	-3.381137	4.000970	-1.061003
9	8	0	-1.715248	5.311785	0.744697

10	8	0	-0.321011	1.185497	-0.304003
11	6	0	0.774696	0.394407	0.112997
12	6	0	0.793908	-0.982893	-0.598103
13	8	0	1.944115	-1.723283	-0.136803
14	6	0	3.154609	-1.111972	-0.254803
15	6	0	3.252297	0.300829	-0.346303
16	6	0	2.078989	1.133119	-0.214403
17	6	0	4.277316	-1.921462	-0.272803
18	6	0	5.537411	-1.314250	-0.393203
19	6	0	5.683199	0.071351	-0.510503
20	6	0	4.548491	0.881541	-0.490803
21	6	0	-0.433984	-1.828104	-0.368403
22	6	0	-0.552277	-2.616705	0.781797
23	6	0	-1.731471	-3.331415	0.991297
24	6	0	-2.801172	-3.256425	0.084697
25	6	0	-2.678579	-2.457424	-1.064303
26	6	0	-1.485385	-1.759213	-1.288103
27	8	0	2.103578	2.368119	-0.327103
28	8	0	4.702679	2.206842	-0.592703
29	8	0	6.607518	-2.148041	-0.407403
30	8	0	-3.949765	-3.950535	0.303197
31	6	0	0.270567	3.638802	2.303597
32	8	0	-3.762379	-2.436234	-1.883703
33	6	0	-3.716887	-1.592233	-3.028203
34	8	0	-1.988564	-4.157618	2.058897
35	6	0	-0.985662	-4.303909	3.053997
36	1	0	-1.861433	3.585883	2.579097

37	1	0	-0.748834	3.761793	-0.246703
38	1	0	-3.566731	3.377668	0.861697
39	1	0	-3.552112	1.241968	-0.096603
40	1	0	-1.711002	0.145985	0.818797
41	1	0	-1.415915	1.571787	-1.788703
42	1	0	-3.068732	3.511273	-1.847103
43	1	0	-2.253851	5.620680	-0.005903
44	1	0	0.739297	0.240707	1.201197
45	1	0	0.913506	-0.797192	-1.675603
46	1	0	4.187726	-2.999063	-0.215803
47	1	0	6.659794	0.535160	-0.613503
48	1	0	0.274323	-2.682498	1.477897
49	1	0	-1.383091	-1.136612	-2.168103
50	1	0	3.795276	2.609534	-0.545303
51	1	0	7.419214	-1.626134	-0.511303
52	1	0	-3.822261	-4.436934	1.136897
53	1	0	0.320257	4.720903	2.463197
54	1	0	1.021069	3.359509	1.559297
55	1	0	0.495971	3.135904	3.250297
56	1	0	-2.944784	-1.921326	-3.736903
57	1	0	-4.697086	-1.684142	-3.499303
58	1	0	-3.535096	-0.546632	-2.750703
59	1	0	-1.394856	-4.992712	3.794997
60	1	0	-0.065258	-4.725701	2.631497
61	1	0	-0.759071	-3.343507	3.533897

Conformer 7-1

Center	Atomic	Atomic	Coordinates (Angstroms)		

Number	Number	Type	X	Y	Z
1	6	0	-1.988614	-2.863883	1.715796
2	6	0	-3.358814	-2.907076	1.029996
3	6	0	-3.201015	-3.196577	-0.468904
4	6	0	-2.218110	-2.223282	-1.124904
5	6	0	-0.927510	-2.164189	-0.296104
6	8	0	-1.194909	-1.860887	1.057796
7	8	0	-2.858104	-0.949179	-1.184104
8	8	0	-4.473115	-3.163670	-1.109204
9	8	0	-4.153419	-3.911972	1.638996
10	8	0	-0.174205	-1.107593	-0.847604
11	6	0	0.963898	-0.648598	-0.138104
12	6	0	1.245905	0.783300	-0.641404
13	8	0	2.421708	1.293494	0.014196
14	6	0	3.544304	0.526388	-0.037404
15	6	0	3.469297	-0.871411	-0.274904
16	6	0	2.189993	-1.530905	-0.379604
17	6	0	4.757107	1.163082	0.165296
18	6	0	5.931003	0.394876	0.125696
19	6	0	5.908896	-0.981024	-0.124804
20	6	0	4.685093	-1.617018	-0.326504
21	6	0	0.105010	1.743306	-0.398704
22	6	0	-0.895889	1.872811	-1.376104
23	6	0	-1.983885	2.708317	-1.136704
24	6	0	-2.089881	3.433217	0.066396
25	6	0	-1.072682	3.323512	1.022796
26	6	0	0.011514	2.469706	0.785896

27	8	0	2.060187	-2.745104	-0.611904
28	8	0	4.672486	-2.936218	-0.554204
29	8	0	7.094407	1.063470	0.327796
30	8	0	-3.178977	4.223323	0.289796
31	6	0	-2.060112	-2.502383	3.189196
32	8	0	-1.131878	3.965312	2.231696
33	6	0	-1.220371	5.393813	2.200796
34	8	0	-3.039584	2.916822	-1.989504
35	6	0	-3.150888	2.084523	-3.141104
36	1	0	-1.503619	-3.847086	1.594796
37	1	0	-3.822809	-1.913974	1.144296
38	1	0	-2.833521	-4.224179	-0.582204
39	1	0	-1.973312	-2.561283	-2.142104
40	1	0	-0.375815	-3.111792	-0.367604
41	1	0	-2.195800	-0.276782	-0.941904
42	1	0	-4.680210	-2.217969	-1.218304
43	1	0	-4.912420	-4.035668	1.043096
44	1	0	0.760798	-0.617397	0.939896
45	1	0	1.453905	0.720199	-1.719704
46	1	0	4.802713	2.231482	0.336196
47	1	0	6.821693	-1.568429	-0.162304
48	1	0	-0.814692	1.326611	-2.309104
49	1	0	0.780614	2.401302	1.546396
50	1	0	3.720585	-3.203813	-0.656204
51	1	0	7.836303	0.440766	0.265996
52	1	0	-3.752477	4.126926	-0.491904
53	1	0	-2.503907	-1.508980	3.315496

54	1	0	-1.058612	-2.494088	3.630596
55	1	0	-2.678216	-3.232780	3.717996
56	1	0	-1.165769	5.715513	3.243096
57	1	0	-2.160969	5.731418	1.757696
58	1	0	-0.376269	5.822508	1.644696
59	1	0	-2.323587	2.264219	-3.839104
60	1	0	-3.175694	1.025323	-2.859204
61	1	0	-4.091187	2.362528	-3.620204

Conformer 7-2

Center	Atomic	Atomic	Coordinates (Angstroms)		
Number	Number	Type	X	Y	Z
1	6	0	-2.139700	-2.699097	1.706999
2	6	0	-3.513900	-2.670896	1.028899
3	6	0	-3.380600	-2.985696	-0.467101
4	6	0	-2.348799	-2.075397	-1.138401
5	6	0	-1.052499	-2.075999	-0.315901
6	8	0	-1.294898	-1.747298	1.037099
7	8	0	-2.919197	-0.769996	-1.207901
8	8	0	-4.652200	-2.890494	-1.101701
9	8	0	-4.361301	-3.621694	1.653499
10	8	0	-0.250098	-1.064200	-0.882301
11	6	0	0.908903	-0.649401	-0.181001
12	6	0	1.244705	0.768198	-0.691601
13	8	0	2.447405	1.230597	-0.044301
14	6	0	3.534104	0.413496	-0.098901
15	6	0	3.403203	-0.978404	-0.328101
16	6	0	2.095902	-1.583603	-0.424701

17	6	0	4.776405	0.998794	0.094099
18	6	0	5.919704	0.182892	0.052799
19	6	0	5.840602	-1.190807	-0.188701
20	6	0	4.590902	-1.773506	-0.381001
21	6	0	0.146906	1.776100	-0.446701
22	6	0	-0.839794	1.956701	-1.424001
23	6	0	-1.893993	2.838102	-1.175801
24	6	0	-1.967492	3.555602	0.027599
25	6	0	-0.965892	3.380401	0.996799
26	6	0	0.081207	2.482500	0.757299
27	8	0	1.912200	-2.791402	-0.650001
28	8	0	4.519300	-3.092106	-0.601801
29	8	0	7.161505	0.700691	0.231399
30	8	0	-2.992191	4.418304	0.262799
31	6	0	-2.182999	-2.323097	3.177799
32	8	0	-1.114991	4.122801	2.128199
33	6	0	-0.154891	3.965800	3.159799
34	8	0	-2.930292	3.107504	-2.035301
35	6	0	-3.076693	2.287904	-3.191101
36	1	0	-1.711001	-3.708898	1.592199
37	1	0	-3.918898	-1.651395	1.133999
38	1	0	-3.070501	-4.033096	-0.570101
39	1	0	-2.126499	-2.438797	-2.152201
40	1	0	-0.549800	-3.050999	-0.381501
41	1	0	-2.211596	-0.129897	-1.008501
42	1	0	-4.806899	-1.936594	-1.224901
43	1	0	-5.128301	-3.710294	1.061399

44	1	0	0.712903	-0.607201	0.897999
45	1	0	1.444305	0.693198	-1.770401
46	1	0	4.844906	2.070694	0.253199
47	1	0	6.738302	-1.795909	-0.223501
48	1	0	-0.776394	1.421301	-2.364401
49	1	0	0.860907	2.341099	1.494299
50	1	0	3.558000	-3.320605	-0.699201
51	1	0	7.091906	1.655091	0.391999
52	1	0	-3.572491	4.371805	-0.517701
53	1	0	-1.180199	-2.366799	3.614299
54	1	0	-2.571598	-1.305997	3.298299
55	1	0	-2.837500	-3.014196	3.715599
56	1	0	0.851309	4.249099	2.823099
57	1	0	-0.131292	2.934600	3.536799
58	1	0	-0.469090	4.636301	3.961599
59	1	0	-2.238593	2.428603	-3.885401
60	1	0	-3.999593	2.614205	-3.673501
61	1	0	-3.155995	1.229504	-2.915401

Conformer 7-3

Center	Atomic	Atomic	Coordinates (Angstroms)		
Number	Number	Type	X	Y	Z
1	6	0	-2.917596	-2.488109	1.125493
2	6	0	-4.194096	-2.136910	0.350193
3	6	0	-3.947296	-2.234510	-1.162107
4	6	0	-2.723397	-1.422309	-1.585107
5	6	0	-1.538397	-1.770008	-0.675707
6	8	0	-1.869397	-1.602408	0.693693

7	8	0	-3.080398	-0.048209	-1.470407
8	8	0	-5.114697	-1.846010	-1.877807
9	8	0	-5.226496	-3.030810	0.732493
10	8	0	-0.520297	-0.855508	-1.009107
11	6	0	0.634103	-0.831807	-0.184007
12	6	0	1.349402	0.507793	-0.403007
13	8	0	2.515002	0.544594	0.470293
14	6	0	3.390203	-0.488505	0.361193
15	6	0	2.987503	-1.739906	-0.167707
16	6	0	1.605303	-1.973006	-0.510207
17	6	0	4.689002	-0.277405	0.801293
18	6	0	5.611803	-1.332204	0.707593
19	6	0	5.265004	-2.575404	0.171293
20	6	0	3.960604	-2.783405	-0.267107
21	6	0	0.554901	1.766693	-0.146107
22	6	0	-0.657099	1.770192	0.552993
23	6	0	-1.316299	2.983592	0.759893
24	6	0	-0.777700	4.194892	0.303193
25	6	0	0.445200	4.187593	-0.388607
26	6	0	1.095201	2.971493	-0.616007
27	8	0	1.188504	-3.036207	-0.999707
28	8	0	3.629105	-3.980705	-0.768007
29	8	0	6.896103	-1.184003	1.121393
30	8	0	-1.431201	5.368992	0.507993
31	6	0	-3.063396	-2.340309	2.629993
32	8	0	0.890199	5.407493	-0.800707
33	6	0	2.113899	5.463294	-1.513507

34	8	0	-2.521499	3.135391	1.401493
35	6	0	-3.247599	1.959591	1.745293
36	1	0	-2.636296	-3.525709	0.880893
37	1	0	-4.466497	-1.097410	0.594593
38	1	0	-3.780096	-3.290909	-1.406607
39	1	0	-2.453397	-1.661209	-2.624707
40	1	0	-1.203496	-2.801608	-0.848907
41	1	0	-2.252298	0.462891	-1.495307
42	1	0	-5.095997	-0.872810	-1.904407
43	1	0	-5.931996	-2.907911	0.073693
44	1	0	0.351403	-0.921807	0.872893
45	1	0	1.726302	0.525794	-1.436307
46	1	0	4.970102	0.695196	1.193893
47	1	0	5.999204	-3.368904	0.102993
48	1	0	-1.095798	0.847692	0.910693
49	1	0	2.037601	2.951594	-1.151207
50	1	0	2.666405	-3.941506	-1.005507
51	1	0	7.015902	-0.291203	1.482093
52	1	0	-2.252601	5.143791	0.980293
53	1	0	-3.315797	-1.306109	2.890093
54	1	0	-2.129796	-2.607609	3.134593
55	1	0	-3.863696	-2.993409	2.987993
56	1	0	2.278899	6.518394	-1.738707
57	1	0	2.062200	4.896094	-2.452907
58	1	0	2.951099	5.085594	-0.911007
59	1	0	-2.724398	1.385091	2.520193
60	1	0	-3.404998	1.323491	0.866393

61	1	0	-4.206899	2.303590	2.135893

2. Experimental and Computed NMR Chemical Shifts

^{13}C-NMR chemical shifts

The TMS-corrected computed ^{13}C-NMR chemical shifts of compounds **1** were fitted to the experimental values by Ordinary Least Squares (OLS) Linear Regression method in order to remove systematic error that results from the conformational search and random error from experimental conditions (**Table S3**).

Table S3 Experimental and computed chemical shifts of **1a** and **1b**.

Position	Exp.**1**	**1a**	Residue	**1b**	Residue
1	173.1	174.7255548	-1.625554789	175.8554613	-2.755461298
2	105.1	116.920939	-11.82093898	105.4891705	-0.389170475
3	132	137.4083222	-5.40832223	135.4627254	-3.462725408
4	122.2	126.3955808	-4.195580765	122.4494645	-0.24946448
5	132.4	132.6455671	-0.245567077	132.6181887	-0.218188691
6	106.5	100.6459063	5.854093689	103.4345241	3.065475915
7	156.2	153.7004265	2.499573489	154.9537144	1.246285648
8	100.8	97.39681796	3.403182043	96.53117989	4.268820108
9	153.6	151.5022128	2.097787232	152.7241366	0.875863439
10	132.8	132.974323	-0.174323029	132.0754579	0.724542128
11	21.9	21.82035253	0.079647468	25.14355442	-3.243554424
12	39.1	40.62481165	-1.524811653	39.51365811	-0.413658111
13	77.3	72.83520733	4.464792669	76.75772643	0.542273574
14	20.7	20.82599359	-0.125993594	20.41780457	0.282195431
15	171.9	165.1779845	6.722015525	172.1732334	-0.273233355
16	56.7	54.1049642	2.595035803	54.05956102	2.640438982

Table S4 Statistics of Ordinary Least Squares Linear Regression (OLS-LR) of experimental and computed ^{13}C-NMR chemical shifts.

Compound	CMAD[a]	CLAD[b]	R^2	*RMSE*	*F*	*p* value
**1a**	3.37	11.96	0.9916	4.7889	1646.83	< 0.01

**1b**	1.57	4.04	0.9982	2.2282	7658	< 0.01

[a]CMAD = corrected mean absolute deviation, computed as $(1/n)\sum_{i}^{n}|\delta_{\mathrm{calc}}-\delta_{\mathrm{exp}}|$, where δ_{calc} and δ_{exp} refer to the calculated and experimental chemical shifts. [b]CLAD = corrected largest absolute deviation, computed as $\max(|\delta_{\mathrm{calc}}-\delta_{\mathrm{exp}}|)$.

DP4+ analysis of compound 1 with two possible structures of 1a and 1b.

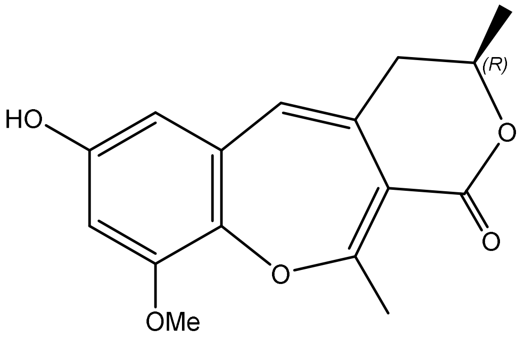

1a

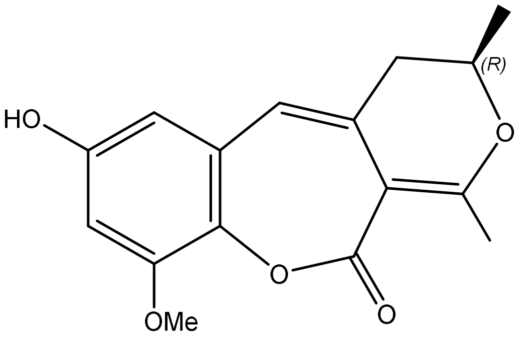

1b

(DP4^{+}:0.00**%)-1a**

(DP4^{+}:100.00**%)-1b**

Figure S71 DP4+ probability for compounds 1a and 1b based on its ^{1}H and ^{13}C-NMR date.

Functional		Solvent?		Basis Set		Type of Data	
mPW1PW91		PCM		6-311G(d,p)		Unscaled Shifts	
	Isomer 1	Isomer 2	Isomer 3	Isomer 4	Isomer 5	Isomer 6	
sDP4+ (H data)	4.33%	95.67%	-	-	-	-	
sDP4+ (C data)	0.00%	100.00%	-	-	-	-	
sDP4+ (all data)	0.00%	100.00%	-	-	-	-	
uDP4+ (H data)	0.78%	99.22%	-	-	-	-	
uDP4+ (C data)	0.00%	100.00%	-	-	-	-	
uDP4+ (all data)	0.00%	100.00%	-	-	-	-	
DP4+ (H data)	0.04%	99.96%	-	-	-	-	
DP4+ (C data)	0.00%	100.00%	-	-	-	-	
DP4+ (all data)	0.00%	100.00%	-	-	-	-	

Functional		Solvent?		Basis Set		Type of Data	
mPW1PW91		PCM		6-311G(d,p)		Unscaled Shifts	
		DP4+	0.00%	100.00%	-	-	-
Nuclei	sp2?	xperimenta	Isomer 1	Isomer 2	Isomer 3	Isomer 4	Isomer 5
C	x	173.1	183.486613	182.130877			
C	x	105.1	122.614285	109.304815			
C	x	132	144.188941	140.326145			
C	x	122.2	132.591749	126.857984			
C	x	132.4	139.173425	137.382173			
C	x	106.5	105.47553	107.178345			
C	x	156.2	161.345673	160.498474			
C	x	100.8	102.054011	100.033683			
C	x	153.6	159.030799	158.190958			
C	x	132.8	139.519628	136.82047			
C		21.9	22.4666754	26.1505838			
C		39.1	42.269094	41.0230184			
C		77.3	76.1889071	79.5690152			
C		20.7	21.4195454	21.2596375			
C	x	171.9	173.432349	178.31993			
C		56.7	56.0385359	55.6525851			
H	x	6.07	6.78766009	6.29850578			
H	x	6.15	6.15461994	6.38138584			
H	x	6.4	6.61153293	6.35092184			
H		2.31	2.5336059	2.33926308			
H		2.64	2.76201665	2.41079418			
H		2.35	2.54010058	2.4433635			
H		4.45	4.41509475	4.46862224			
H		1.34	1.30383081	1.31272611			

Experimental and calculated ECD spectra

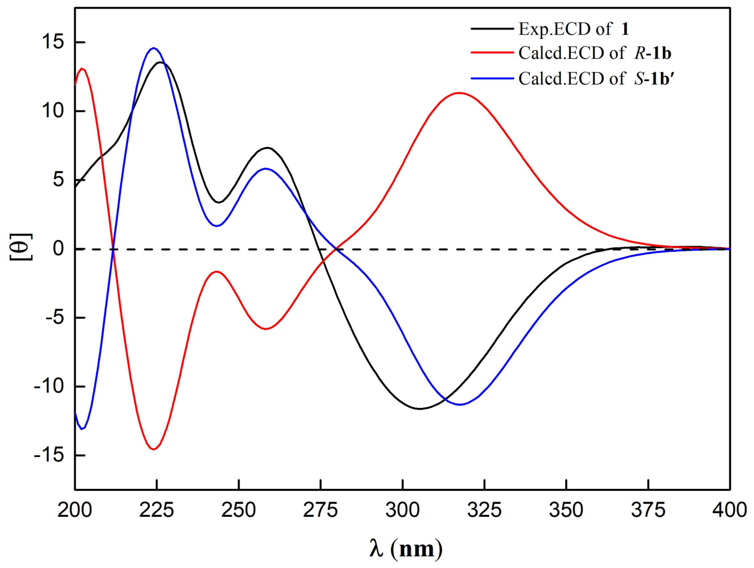

Figure S72 Calculated ECD spectra of compounds 1 was compared with the experimental.

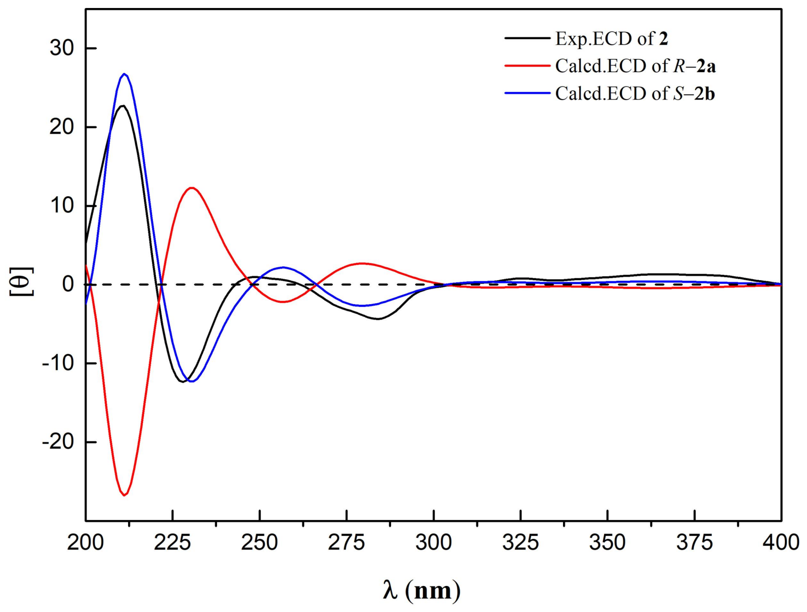

Figure S73 Calculated ECD spectra of compounds 2 was compared with the experimental.

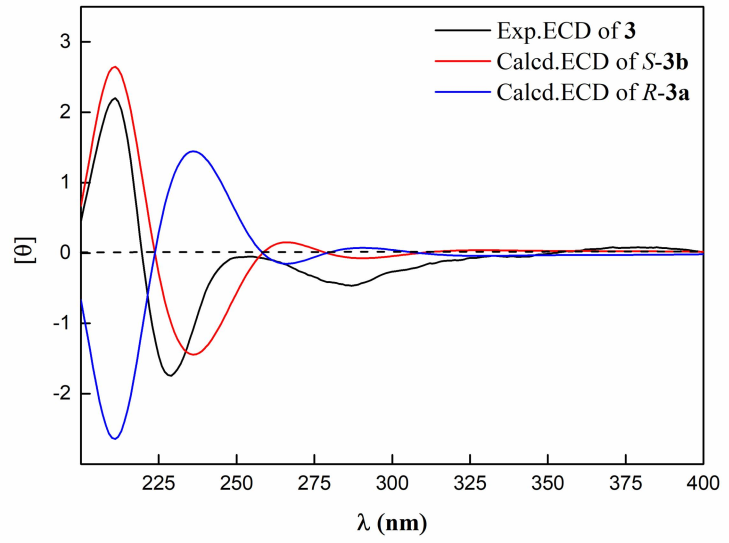

Figure S74 Calculated ECD spectra of compounds 3 was compared with the experimental.

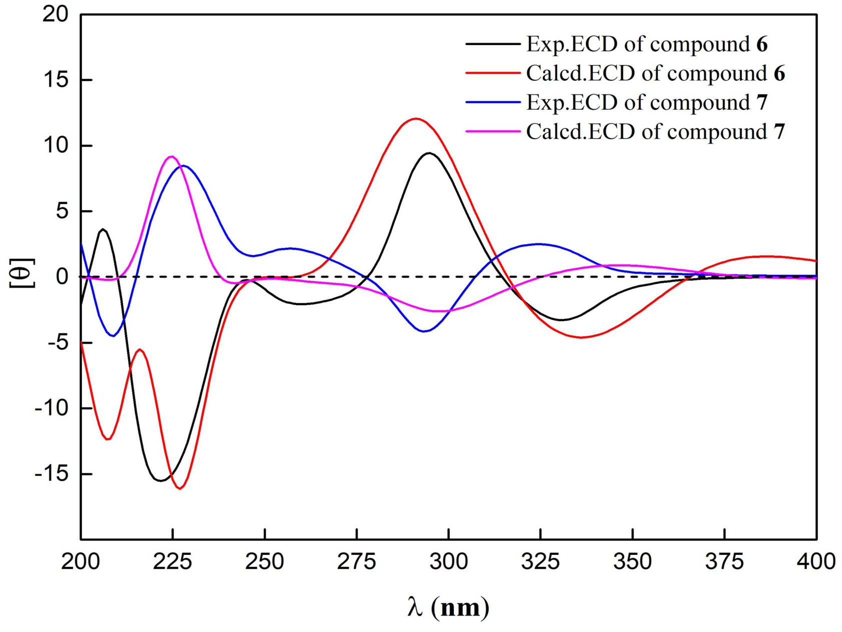

Figure S75 Calculated ECD spectra of compounds 6 and 7 was compared with the experimental.
